# Synthesis and Characterization of Novel 2-Alkyl-1,3,4-Oxadiazoles Containing a Phenylazo Group

**DOI:** 10.3390/molecules29184316

**Published:** 2024-09-11

**Authors:** Sebastian Górecki, Agnieszka Kudelko

**Affiliations:** Department of Chemical Organic Technology and Petrochemistry, The Silesian University of Technology, Krzywoustego 4, PL-44100 Gliwice, Poland; sg301135@student.polsl.pl

**Keywords:** heterocycles, 1,3,4-oxadiazole, reduction, diazotization, coupling, phenylazo moiety

## Abstract

An efficient method for the synthesis of novel phenylazo-containing moieties is described. The derivatives of 5-(4-(phenyldiazenyl)phenyl)-1,3,4-oxadiazole, substituted at position 2 of the heterocyclic scaffold with alkyl groups of different chain lengths, were prepared. The titled compounds were obtained using the appropriate 4-(5-alkyl-1,3,4-oxadiazol-2-yl)anilines, which were directed to diazotization and subsequently coupled to phenol, resorcinol, and *N*,*N*-dimethylaniline. Additionally, we report a mild and effective procedure for the preparation of 4-(5-alkyl-1,3,4-oxadiazol-2-yl)anilines via the selective reduction of the corresponding 2-alkyl-5-(4-nitrophenyl)-1,3,4-oxadiazoles using sodium borohydride-tin(II) chloride dihydrate as the reducing system. The chemical structures of the prepared compounds were confirmed by ^1^H- and ^13^C-NMR, IR, and UV-Vis spectroscopy.

## 1. Introduction

1,3,4-Oxadiazoles are a group of non-naturally occurring five-membered heterocyclic arrangements composed of two neighboring nitrogen atoms and one oxygen atom with carbon atoms. They have gained interest among researchers due to their broad spectrum of biological activity, such as anti-inflammatory, analgesic, anticancer, antibacterial, antifungal, antiviral, or blood-pressure-lowering interactions [1,2,3,4,5,6]. Except for medicine, 1,3,4-oxadiazoles have also been applied to agriculture in crop protection as pesticides fighting against weeds, insects, bacteria, and fungi [7,8,9]. Conjugated arrangements composed of such a core show interesting electron transfer or luminescent properties and can be used in the production of different types of conducting systems, including organic light-emitting diodes (OLEDs), laser dyes, scintillators, or optical brighteners [10,11,12,13]. These compounds have also been applied in materials science, where they act as anti-corrosion or blowing agents, or monomers for the production of heat-resistant polymers [14,15,16,17]. The extensive applicability of 1,3,4-oxadiazoles is the main reason for the exploration of efficient preparation methods. The most popular procedure employs *N*,*N′*-diacylhydrazines, which undergo cyclodehydration under the influence of sulfuric acid [18], thionyl chloride [19], polyphosphoric acid [20], phosphoryl chloride [21], trifluoromethanesulfonic anhydride [22], phosphorus pentoxide [23], or triphenylphosphine derivatives [24]. Another approach uses *N*-acylhydrazones in the synthesis of 1,3,4-oxadiazoles, where they undergo oxidative cyclization in the presence of 2,3-dichloro-5,6-dicyano-1,4-benzoquinone (DDQ) [25], cerium ammonium nitrate (CAN) [26], potassium permanganate (KMnO_4_) [27], iron(III) chloride (FeCl_3_) [28], tetravalent lead reagents [29], iodine (I_2_/HgO), [30], bromine (Br_2_/NaOAc) [31], chloramine T [32], and hypervalent iodine reagents [33]. Other methods for 1,3,4-oxadiazole formation include the reaction of acid hydrazides with carboxylic acids [34], aromatic aldehydes [26], or triethyl orthoesters [35], isomerization of 1,2,4-oxadiazoles [36], as well as the transformation of tetrazoles [37].

Azo dyes constitute the largest and most important class of synthetic dyes, in terms of production volume, consumption, and their possible applications [38,39,40,41]. They are characterized by the presence of one, two, or more chromophoric azo groups −N=N− connecting individual organic fragments, usually aromatic, into the dye molecule. Some such dyes, in addition to an azo group, contain other chromophores or functional groups that deepen the color (auxochromes, e.g., hydroxyl or amino groups and their substituted alkyl or aryl derivatives), modify the affinity to the different materials, and have solubility (e.g., carboxyl or sulfonic groups) [42]. The industrial process for the production of azo compounds is a two-step reaction sequence comprised of diazotization of primary aromatic amines and then coupling with an activated aromatic compound [43]. In general, the azo dyes are highly colorant and exhibit vivid colors, starting from yellows and oranges, through reds and violets, and ending even in blues or greens. A wide range of colors, strong coloring properties, affinity to various materials, and easy preparation promote their broad application in the dyestuff industry, food, cosmetics, photography, and pharmacy [43].

**Figure 1:**
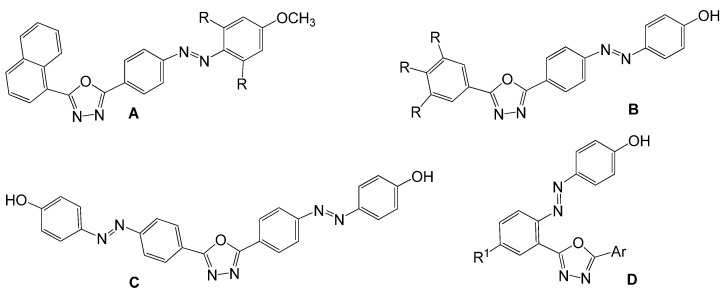
Literature examples of 1,3,4-oxadiazoles decorated with azobenzene for optoelectronic and medical applications.

An interesting but relatively rarely investigated group of azo compounds are derivatives composed of 1,3,4-oxadiazole and azobenzene cores, which, in addition to intense color, exhibit other useful properties. 1,3,4-Oxadiazole-decorated azobenzenes have been used successfully in irradiation experiments to study their photoswitching potential (**A**, Figure 1) [44], while new highly fluorescent gelators based on 2-[(4-hydroxyphenyl)azophenyl]-1,3,4-oxadiazole (**B**, Figure 1) are promising precursors for the construction of photo-controllable luminescence molecular devices [45,46]. Reversibly switchable azomacrocycles derived from the symmetrically substituted 1,3,4-oxadiazole scaffold (**C**, Figure 1) were investigated by Zheng et al. [47]. In addition, Chen et al. synthesized a few groups of 2,5-disubstituted 1,3,4-oxadiazoles bearing azophenols and studied their anticancer activity (**D**, Figure 1) [48].

**Scheme 1:**
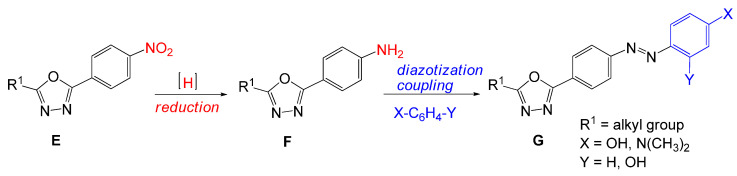
Key reagents and basic reactions for the synthesis of 2-alkyl-5-(4-(phenyldiazenyl)phenyl)-1,3,4-oxadiazoles: 2-alkyl-5-(4-nitrophenyl)-1,3,4-oxadiazoles and the primary aromatic amines—4-(5-alkyl-1,3,4-oxadiazol-2-yl)anilines.

Here, we synthesized and examined another group of conjugated 1,3,4-oxadiazoles. The synthesis of novel azo compounds was studied, in particular 5-(4-(phenyldiazenyl)phenyl)-1,3,4-oxadiazole derivatives, substituted at position 2 of the heterocyclic scaffold with alkyl groups of a different chain length (**G**, Scheme 1). We obtained such compounds based on the classic diazotization reaction of 4-(5-alkyl-1,3,4-oxadiazol-2-yl)anilines (**F**) and the subsequent coupling of the resulting diazonium salts with selected activated aromatics: phenol, resorcinol, and *N*,*N*-dimethylaniline (Scheme 1).

The corresponding conjugated nitro derivatives—2-alkyl-5-(4-nitrophenyl)-1,3,4-oxadiazoles (**E**), obtained from acyclic reagents, served as precursors for the synthesis of 4-(5-alkyl-1,3,4-oxadiazol-2-yl)anilines (**F**) in the preceding reduction reaction.

## 2. Results and Discussion

**Scheme 2:**
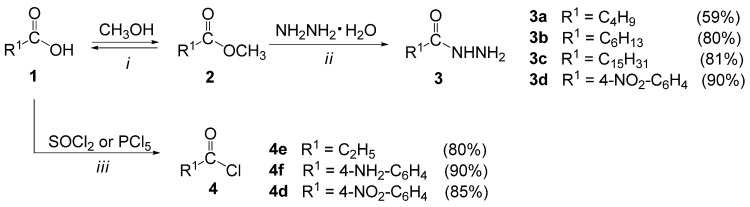
The synthesis of acid hydrazides (**3a**–**d**) and acid chlorides (**4d**–**f**). Reaction conditions: (*i*) conc. H_2_SO_4_, reflux, 4–12 h; (*ii*) CH_3_OH, reflux, 3 h; (*iii*) reflux, 2–20 h.

The new azo dyes containing 2-alkyl-1,3,4-oxadiazole scaffolds were prepared using derivatives of selected aliphatic and aromatic carboxylic acids, including hydrazides (**3a**–**d**) and acid chlorides (**4d**–**f**, Scheme 2). Acid hydrazides (**3a**–**d**) were generated via a two-step reaction sequence involving the esterification of carboxylic acids (**1**) with methanol in the presence of catalytic amounts of sulfuric acid, and subsequent substitution in the ester molecule (**2**) in the presence of hydrazine hydrate. Acid chlorides, including propanoyl chloride (**4e**) and 4-aminobenzoyl chloride (**4f**), were obtained by treating the appropriate carboxylic acid with thionyl chloride (SOCl_2_). 4-Nitrobenzoyl chloride (**4d**) was synthesized from 4-nitrobenzoic acid, where phosphorus pentachloride (PCl_5_) was used as the chlorinating agent (Scheme 2).

**Scheme 3:**
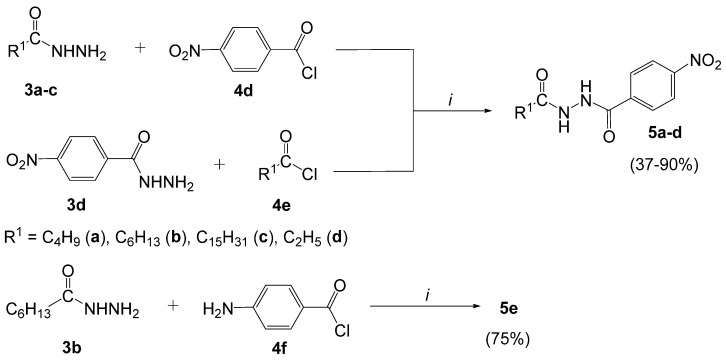
Preparation of selected *N,N′*-diacylhydrazines (**5a**–**e**)—reagents for cyclodehydration. Reaction conditions: (*i*) (C_2_H_5_)_2_O, Na_2_CO_3_ aq., 1 h, rt.

Then, hydrazides (**3a**–**d**) and acid chlorides (**4e**–**d**) were used in the preparation of acyclic *N,N′*-diacylhydrazines (**5a**–**d**)—precursors of 1,3,4-oxadiazoles containing alkyl groups (Scheme 3). The substitution reaction of 4-nitrobenzoyl chloride (**4d**) with acid hydrazides (**3a**–**c**) was carried out in dry diethyl ether at room temperature. The crude products (**5a**–**c**) precipitated after the addition of 4-nitrobenzoyl chloride (**4d**) into the reaction mixture, which was removed by filtration and washed with an aqueous solution of sodium carbonate (Na_2_CO_3_) to eliminate the hydrogen chloride (HCl) residue. Issues arose due to the effective removal of water from propionic acid hydrazide after the reaction of hydrazinolysis. Thus, for the synthesis of *N′*-propionyl-4′-nitrobenzohydrazide (**5d**), the reverse variant was used, in which 4-nitrobenzoic acid hydrazide (**3d**) and propanoyl chloride (**4e**) were applied in an analogous procedure. As a result of the reactions performed, five *N*′-alkanoyl-4-nitrobenzohydrazides were obtained with varying yields (**5a**–**e**, 37–90%, Table 1).

The investigation of the cyclization of *N*,*N′*-diacylhydrazines (**5a**–**d**) to the corresponding 2-alkyl-1,3,4-oxadiazoles (**6a**–**d**) was conducted by first using *N′*-heptanoyl-4-nitrobenzohydrazide (**5b**) and phosphorus oxychloride (POCl_3_) as they have been previously successfully applied to the synthesis of 2,5-diaryl derivatives of 1,3,4-oxadiazole [49].

**Scheme 4:**
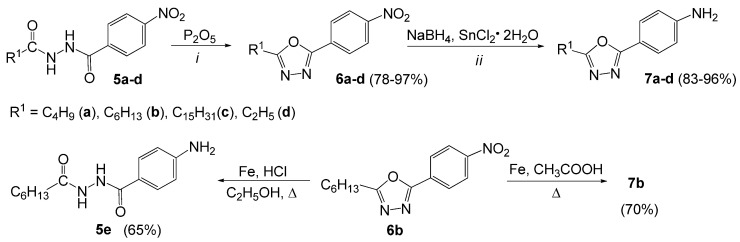
Preparation of 2-alkyl-5-(4-nitrophenyl)-1,3,4-oxadiazoles (**6a**–**d**) and their subsequent reduction to 4-(5-alkyl-1,3,4-oxadiazol-2-yl)anilines (**7a**–**d**) and 4-amino-*N′*-heptanoylbenzohydrazide (**5e**). Reaction conditions: (*i*) toluene, oil bath 120 °C, 1–3 h; (*ii*) C_2_H_5_OH, rt, 1 h.

The procedures were tested using an inert hydrocarbon solvent such as toluene (Table 2, Entries 1–2) and solvent-free (Table 2, Entry 3). When compound **5b** was heated in excess POCl_3_, acting both as a cyclodehydrating agent and solvent, the desired product **6b** was generated in a moderate yield of 47% (Table 2, Entry 3). Subsequently, thionyl chloride (SOCl_2_) was examined, providing better results (78%); however, the reaction time was longer (Table 2, Entry 6). Among the studied cyclodehydrating agents, diphosphorus pentoxide (P_2_O_5_) in toluene gave the best results, generating product **6b** in a high yield of 80% after a short reaction time (Table 2, Entry 9). The elaborated conditions were used in the cyclization of *N,N′*-diacylhydrazines (**5a**–**d**, Scheme 4). Heating compounds **5a**–**d** in excess P_2_O_5_ and dry toluene, followed by processing of the post-reaction mixture, gave 2-alkyl-5-(4-nitrophenyl)-1,3,4-oxadiazoles **6a**–**d** in high yields (78–97%, Table 3).

Next, the synthesized aromatic nitro derivatives were reduced. Since compounds **6a**–**d** bear two units sensitive to reducing agents, the 1,3,4-oxadiazole ring, and nitro group (NO_2_), the reducing agent, and the environment had to be specifically tailored to achieve selective reduction. Literature reports show the reduction of 2-(4-nitrophenyl)-1,3,4-oxadiazole derivatives by catalytic hydrogenation over Pd/C [50], or using SnCl_2_ in methanol [51], Na_2_S in water [52], or Fe/NH_4_Cl in ethanol [53]. However, the latter methods require long reaction times and usually harsh conditions. The first reduction reaction studied involved a typical reduction system: iron filings in a concentrated HCl solution (Scheme 4). Heating 2-hexyl-5-(4-nitrophenyl)-1,3,4-oxadiazole (**6b**) in ethanol for 2 h reduced the nitro group on the benzene ring, and at the same time, the 1,3,4-oxadiazole core underwent ring opening. Hence, the main reaction product was a reduced acyclic 4-amino-*N′*-heptanoylbenzohydrazide (**5e**, 65%, Scheme 4). To confirm the identity of product **5e**, it was also prepared by a previously presented method using heptane hydrazide **3b** and 4-aminobenzoyl chloride (**4f**, Scheme 3), and both products were compared using spectroscopic analyses. Another reduction attempt utilized glacial acetic acid, representing a group of lower-strength organic acids. The heating of compound **6b** with iron filings in glacial acetic acid for several hours promoted the reduction of the nitro group, producing derivative 4-(5-hexyl-1,3,4-oxadiazol-2-yl)aniline (**7b**) in a good yield (70%, Scheme 4). A literature report [54] examined the selective reduction of 2-(4-nitrophenyl)thiophene using sodium borohydride (NaBH_4_) and hydrated tin chloride (SnCl_2_·2H_2_O). This approach was examined using 2-phenyl-1,3,4-oxadiazole derivatives substituted with the nitro group. To our delight, the reduction of derivative **6b** occurred selectively, where reaction occurred without destruction of the aromatic benzene and 1,3,4-oxadiazole rings at room temperature within 1 h (83%, Table 3, Entry 2). Owing to the mild reaction conditions, short reaction time, high yield, and relatively simple method of isolating the final product, this procedure was used for the synthesis of the remaining reduced derivatives **7a**, **7c**–**d**. As a result, we obtained a series of 4-(1,3,4-oxadiazol-2-yl)anilines substituted with alkyl groups of different lengths at position 2 of the 1,3,4-oxadiazole ring (**7a**–**d**) in high yields of 83–96% (Table 3).

**Scheme 5:**
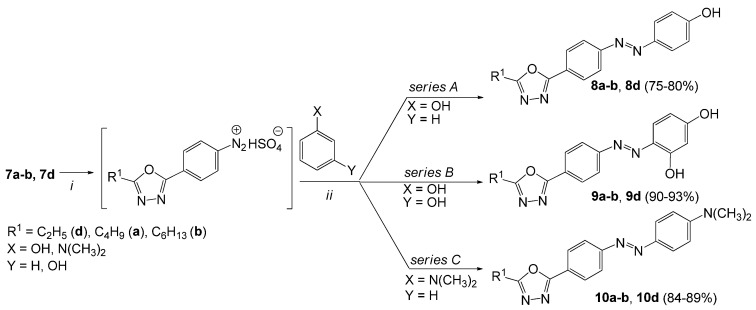
Preparation of 2-alkyl-5-(4-phenylazo)-1,3,4-oxadiazole dyes (**8a**–**b**, **8d**; **9a**–**b**, **9d**; **10a**–**b**, **10d**). Reaction conditions: (*i*) conc. H_2_SO_4_, NaNO_2aq_, 0–5 °C, 20 min; (*ii*) NaOH_aq_ (*series A* and *B*) or C_2_H_5_OH (*series C*), 0–5 °C, 1 h, and NaOH_aq_ to pH = 7.0.

The prepared extended primary aromatic amines were used to study the synthesis of new azo moieties via the diazotization of the selected 4-(1,3,4-oxadiazol-2-yl)anilines (**7a**–**b**, **7d**), and the subsequent coupling of the resulted diazonium salts with phenol, resorcinol, and *N,N*-dimethylaniline (Scheme 5).

The first step involved the diazotization of the aniline derivative (**7a**–**b**, **7d**) dissolved in concentrated H_2_SO_4_ with a nitrosyl cation, which was generated in situ from NaNO_2_ in such a medium, at temperatures of 0–5 °C. Urea was introduced in the final stage to eliminate excess nitrous acid in the post-reaction mixture. The obtained diazonium salts were used in the coupling sequence with three benzene derivatives substituted with electron-donating groups: phenol (X = OH, Y = H, *series A*), resorcinol (X = OH, Y = OH, *series B*), and *N,N*-dimethylaniline (X = N(CH_3_)_2_, Y = H, *series C*). The reactions with acidic coupling agents (phenol, resorcinol) were conducted using 20% aqueous NaOH, while for *N,N*-dimethylaniline, aqueous ethanol served as the reaction medium. The coupling reaction was conducted at a low temperature (0–5 °C) to avoid the decomposition of the unstable diazonium salts. The resulting azo compounds were precipitated from the post-reaction mixture via neutralization with aqueous NaOH solution, which was then removed by filtration, washed with water, and air-dried. This approach generated a series of new 2-alkyl-5-(4-(phenyldiazenyl)phenyl)-1,3,4-oxadiazoles (**8a**–**b**, **8d**; **9a**–**b**, **9d**; **10a**–**b**, **10d**) in satisfactory yields (75–93%, Table 4). However, among them, the simplest isolation methodology that provided the highest yields involved resorcinol (90–93%, Entries 4–6, Table 4). Generally, 2-alkyl-5-(4-(phenyldiazenyl)phenyl)-1,3,4-oxadiazoles are red-brownish substances except for one derivative: 4-{[4-(5-butyl-1,3,4-oxadiazol-2-yl)phenyl]diazenyl}-*N,N*-dimethylaniline (**10b**), which was a dark green solid with a high melting point of 270 °C. They were highly soluble in acetone, DMSO, Et_2_O, isopropanol, and toluene, and insoluble in water and hexane. Importantly, all nine synthesized derivatives were new compounds, not yet described in the literature.

The new intermediates and the final phenylazo compounds (**8a**–**b**, **8d**; **9a**–**b**, **9d**; **10a**–**b**, **10d**) were identified using classical spectroscopic methods (^1^H-NMR, ^13^C-NMR, UV-Vis, FT-IR, and HRMS; see Supplementary Materials). All ^1^H-NMR spectra of the synthesized azo dyes showed the presence of four signals (*series A*, *series C*) or five signals (*series B*) in the aromatic range of 6.35−8.16 ppm, attributed to the protons of the two benzene rings situated at the azo linkage −N=N−. The proton signals related to the alkyl groups located at position 2 of the 1,3,4-oxadiazole were found more upfield at 0.87−2.98 ppm. The number of signals depended on the length of the alkyl chain. For ethyl substituted derivatives (R^1^ = C_2_H_5_, **8d**, **9d**, **10d**), there were two signals: a triplet at ca. 1.35 ppm and a quartet at ca. 2.97 ppm. For butyl substituted derivatives (R^1^ = C_4_H_9_, **8a**, **9a**, **10a**), four signals were observed: a triplet representing the terminal -CH_3_ group at 0.94 ppm, a quintet at 1.77 ppm, a sextet at 1.41 ppm, and a triplet at 2.95 ppm of internal -CH_2_- groups. Finally, for hexyl substituted derivatives (R^1^ = C_6_H_13_, **8b**, **9b**, **10b**), four signals were observed: a triplet representing the terminal -CH_3_ group at 0.87 ppm, a multiplet of three -CH_2_- groups at 1.30−1.39 ppm, and a quintet and triplet representing the internal -CH_2_- groups at ca. 1.78 ppm and 2.95 ppm, respectively. The characteristic hydroxyl group in *series A* (X = OH, **8a**–**b**, **8d**) from the introduced 4-hydroxyphenyl group appeared as a singlet at ca. 10.45 ppm. In the spectra of *series B* (X = OH, Y = OH, **9a**–**b**, **9d**), two characteristic hydroxyl groups were visible as two singlets at 10.71 ppm and 12.24 ppm, respectively, while in *series C* (X = N(CH_3_)_2_, **10a**–**b**, **10d**) that represented the *N,N*-dimethylamino group, they appeared in the range of 2.91–3.36 ppm. In the ^13^C-NMR spectra of the synthesized azo dyes, the characteristic peaks from 1,3,4-oxadiazole carbon atoms C-2 and C-5 were observed at 163.26−163.87 ppm and 166.79−167.55 ppm, respectively. The carbon atoms from two additional benzene rings were found at 102.98−164.63 ppm, while the alkyl groups appeared at 10.4–30.5 ppm. The signal related to the *N,N*-dimethylamino group from *series C* was observed at ca. 48.5 ppm. The formation of 2-alkyl-5-(4-(phenyldiazenyl)phenyl)-1,3,4-oxadiazoles (**8a**–**b**, **8d**; **9a**–**b**, **9d**; **10a**–**b**, **10d**) was also confirmed by HRMS analysis (see Materials and Methods). The FT-IR spectra of intermediates **5a**–**e**, **6a**–**d**, and **7a−d** and the three series of phenylazo derivatives (**8a**–**b**, **8d**; **9a**–**b**, **9d**; **10a**–**b**, **10d**) were found between 4000 and 480 cm^−1^ and further confirmed the changes occurring within the individual functional groups during the reaction sequences. The characteristic strong stretching vibrations of the carbonyl groups in *N′*-alkanoyl-4-nitrobenzohydrazides (**5a**–**d**) visible at 1589–1592 cm^−1^ disappeared during the cyclization to 2-alkyl-5-(4-nitrophenyl)-1,3,4-oxadiazoles (**6a**–**d**). The two sharp stretching bands of the nitro group connected directly to the benzene ring occurred both in acyclic precursors **5a**–**d** at 1510–1520 cm^−1^ and 1343–1349 cm^−1^ and in its cyclic derivatives **6a**–**d** at 1515–1519 cm^−1^ and 1338–1348 cm^−1^, respectively. During the reduction of 2-alkyl-5-(4-nitrophenyl)-1,3,4-oxadiazoles (**6a**–**b**, **6d**) to the corresponding 4-(5-alkyl-1,3,4-oxadiazol-2-yl)anilines (**7a**–**b**, **7d**), the bands mentioned above disappeared, and characteristic stretching modes for the primary aromatic amine appeared at 3446–3413 cm^−1^, 3327–3338 cm^−1^, and 3213–3218 cm^−1^. The latter one showed the possible presence of hydrogen bonding in compounds **7a**–**b**, **7d**. The subsequent diazotization/coupling sequence led to the disappearance of the stretching vibrations of the amino group in the spectra of all azo compounds (*series A*–*C*). Instead of three representative bands, we observed a broad band of variable intensity in the range of 3100–3500 cm^−1^, resulting from stretching vibrations of the hydroxyl groups in two series of azo compounds (**8a**–**b**, **8d**, *series A* and **9a**–**b**, **9d**, *series B*).

**Figure 2:**
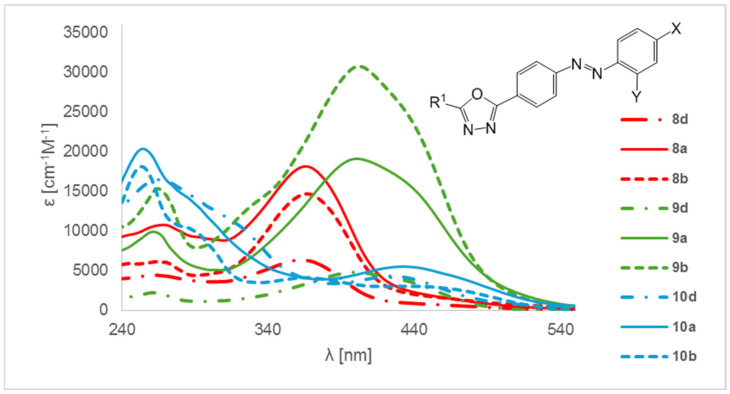
UV-Vis absorption spectra of the synthesized 2-alkyl-5-(4-(phenyldiazenyl)phenyl)-1,3,4-oxadiazoles in aqueous methanol at a concentration of 2.0 × 10^−5^ M at room temperature. (**a**) R^1^ = C_4_H_9_, (**b**) R^1^ = C_6_H_13_, (**d**) R^1^ = C_2_H_5_; *series A* (**8a**–**b**, **8d**; X = OH, Y = H), *series B* (**9a**–**b**, **9d**, X = OH, Y = OH), *series C* (**10a**–**b**, **10d**, X = N(CH_3_)_2_, Y = H).

The UV-Vis spectra of 2-alkyl-5-(4-(phenyldiazenyl)phenyl)-1,3,4-oxadiazoles (**8a**–**b**, **8d**; **9a**–**b**, **9d**; **10a**–**b**, **10d**) registered in aqueous methanol within the range of 240 nm to 600 nm showed the presence of two or even three absorption bands resulting from n→π* and π→π* electronic transitions (Figure 2).

**Figure 3:**
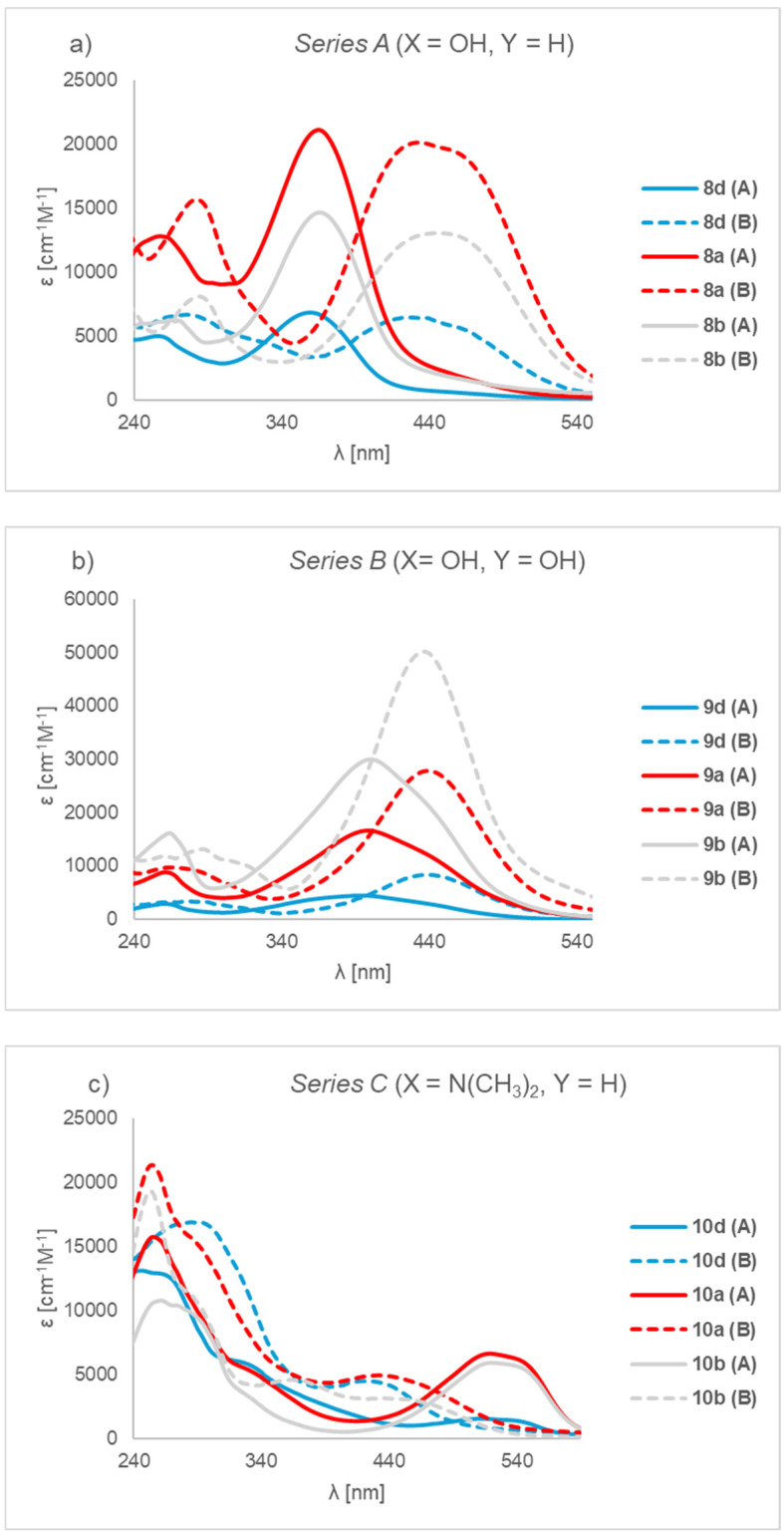
UV-Vis absorption spectra of the three series of azo compounds in aqueous methanol at a concentration of 2.0 × 10^−5^ M at room temperature in acidic and basic solutions: (**a**) absorption spectra of azo compounds **8a**–**b**, **8d** (*series A*) derived from phenol; (**b**) absorption spectra of azo compounds **9a**–**b**, **9d** (*series B*) derived from resorcinol; (**c**) absorption spectra of azo compounds **10a**–**b**, **10d** (*series C*) derived from *N,N*-dimethylaniline. (**A**) acidic media (CH_3_OH, H_2_O, 0.1M HCl; 9:1 *v*/*v*), (**B**) basic media (CH_3_OH, H_2_O, 0.1 M NaOH; 9:1, *v*/*v*); (**a**) C_4_H_9_, (**b**) C_6_H_13_, (**c**) C_2_H_5_.

The influence of the alkyl chain’s length at position 2 of 5-(4-(phenyldiazenyl)phenyl)-1,3,4-oxadiazole dyes within *series A–C* on the location of the absorption maxima was rather small and varied from 2 to 8 nm for the corresponding absorption bands. In addition, Figure 3 shows the UV-Vis absorption spectra of the three series of azo dyes (Figure 3a–c) measured both in acidic aqueous methanol (A) and basic aqueous methanol (B) solutions. The presence of hydroxyl and dimethylamino groups in the studied azo compounds rendered the compounds susceptible to acid–base interactions.

Considerable changes in the spectra of the ionized form in basic media (NaOH, aqueous methanol, —) and acidic media (HCl, aqueous methanol, —) were observed, especially for the long-wavelength bands. In the case of *series A* derived from phenol (Figure 3a, **8a**–**b**, **8d**), alkalization resulted in a batochromic shift of the n→π* band by 66−80 nm, which was accompanied by a slight hypochromic effect. A weaker batochromic shift (36−44 nm) of the n→π* band was also observed in *series B* (Figure 3b, **9a**–**b**, **9d**) containing a 2,4-dihydroxyphenyl group. However, the spectra of the ionized forms of the studied compounds **9a**–**b**, and **9d** registered in basic media, showed a considerable hyperchromic effect compared to the neutral forms in acidic media. Finally, in the case of *series C* derived from *N,N*-dimethylaniline (Figure 3c, **10a**–**b**, **10d**), acidification caused a marked batochromic shift of the n→π* band by 84−88 nm, which was accompanied by a slight hyperchromic effect.

## 3. Materials and Methods

### 3.1. General Information

All reagents were purchased from commercial sources and used without further purification. Melting points were measured using a Stuart SMP3 melting point apparatus (Staffordshire, UK). NMR spectra were recorded at 25 °C using an Agilent spectrometer (Agilent Technologies, Waldbronn, Germany) at 400 MHz for ^1^H and 100 MHz for ^13^C, and a Varian spectrometer (Palo Alto, CA, USA) at 600 MHz for ^1^H and 151 MHz for ^13^C, respectively, with CDCl_3_ or DMSO as the solvent and TMS as the internal standard. UV-Vis spectra were recorded in methanol and dichloromethane solutions (2.0 × 10^−5^ M) with a Jasco V-650 spectrophotometer (Jasco International CO., Tokyo, Japan). High-resolution mass spectra were acquired using a Waters ACQUITY UPLC/Xevo G2QT instrument (Waters Corporation, Milford, MA, USA). Thin-layer chromatography (TLC) was performed using silica gel 60 F254 (Merck, Merck KGaA, Darmstadt, Germany) on thin-layer chromatography plates, with Benzene:AcOEt (1:3), MeOH:CHCl_3_ (1:4), or Hexane:AcOEt (1:2) as the mobile phase.

### 3.2. Synthesis and Characterization

#### 3.2.1. General Procedure for the Synthesis of Methyl Esters (**2a**–**d**)

A solution of carboxylic acid (**1a**–**d**, 0.25 mol), methanol (100 mL, 2.5 mol), and concentrated sulfuric acid (5 mL) was refluxed until the acid was fully consumed (monitored by TLC, 4–12 h). After cooling, the mixture was neutralized with NaHCO_3_ and extracted with 3 × 20 mL of dichloromethane. The organic phase was dried over MgSO_4_ and concentrated on a rotary evaporator. The crude methyl esters (**2a**–**d**) were used for subsequent reactions without additional purification.

*Methyl valerate* (**2a**). The product was obtained as a colorless liquid (24.40 g, 84%) [55].
*Methyl heptanoate* (**2b**). The product was obtained as a colorless liquid (34.20 g, 95%) [56].
*Methyl palmitate* (**2c**). The product was obtained as a white solid (54.50 g, 85%); mp 29–30 °C (28–30 °C [57]).
*Methyl 4-nitrobenzoate* (**2d**). The product was obtained as yellow solid (39.80 g, 88%); mp 101–102 °C (99–100 °C [58]).

#### 3.2.2. General Procedure for the Synthesis of Acid Hydrazides (**3a**–**d**)

A solution of methyl ester (**2a**–**d**, 0.20 mol), hydrazine hydrate (28 mL, 0.55 mol), and methanol (100 mL) was refluxed until the ester was fully consumed (monitored by TLC, 4–19 h). After cooling, the mixture was concentrated on a rotary evaporator. The crude hydrazides were crystallized from ethanol or methanol.

*Valeryl hydrazide* (**3a**). The product was obtained as a white solid (13.70 g, 59%); mp 63–64 °C (50.5–51.5 °C [59]).
*Heptanehydrazide (***3b**). The product was obtained as a white solid (23.00 g, 80%); mp 87–88 °C (82–84 °C [60]).
*Palmitohydrazide* (**3c**). The product was obtained as a white solid (43.80 g, 81%); mp 112–114 °C (112–113 °C [61]).
*4-Nitrobenzohydrazide* (**3d**). The product was obtained as a yellow solid (30.80 g, 85%); mp 220–221 °C (217–218 °C [62]).

#### 3.2.3. General Procedure for the Synthesis of Acid Chlorides (**4d**–**f**)

A mixture of carboxylic acid (**1d**–**f**, 0.20 mol) and PCl_5_ (41.65 g, 0.20 mol) or SOCl_2_ (29 mL, 0.40 mol) was refluxed until the acid was fully consumed (monitored by TLC, 2–20 h). After cooling, the mixture was concentrated using a rotary evaporator. The crude acid chlorides (**4d**–**f**) were distilled under reduced pressure.

*Propionyl chloride* (**4e**). The product was obtained as a colorless liquid (14.80 g, 80%); bp 78–80 °C (bp 77–79 °C [63]).
*4-Aminobenzoyl chloride* (**4f**). The product was obtained as a yellow solid (27.30 g, 90%); bp 168–170 °C/33 mm Hg, mp 35–36 °C (bp 168–170 °C/33 mm Hg, mp 31–39 °C [64]).
*4-Nitrobenzoyl chloride* (**4d**). The product was obtained as a yellow solid (31.50 g, 85%); bp 175–180 °C/25 mm Hg, mp 71–74 °C (bp 194–196 °C/25 mm Hg, mp 71–73 °C [65]).

#### 3.2.4. General Procedure for the Synthesis of *N*,*N*′-Diacylhydrazines (**5a**–**e**)

Hydrazide (**3a**–**d**, 0.02 mol) was suspended in dry diethyl ether (30 mL); then, acid chloride (**4d**–**f**, 0.02 mol) dissolved in dry diethyl ether (10 mL) was carefully added at room temperature. The reaction mixture was agitated at room temperature for approx. 1 h, followed by the addition of a Na_2_CO_3_ solution (2.12 g, 0.02 mol) in water (15 mL). The mixture was agitated for 30 min, and the white precipitate was filtered, washed with diethyl ether (20 mL), and air dried.

*4-Nitro-N′-pentanoylbenzohydrazide* (**5a**). The product was obtained as a white solid (4.70 g, 90%); mp 187–188 °C (188–190 °C [52]). UV-Vis (CH_3_OH) λ_max_ (logε) 202 (4.19), 262 (4.12) nm.
*N′-Heptanoyl-4-nitrobenzohydrazide* (**5b**). The product was obtained as awhite solid (5.30 g, 88%); mp 181–182 °C. ^1^H-NMR (400 MHz, DMSO-d_6_): δ 10.63 (s, 1H, NH), 9.96 (s, 1H, NH), 8.34 (d, 2H, *J* = 8.8 Hz, Ar), 8.09 (d, 2H, *J* = 8.8 Hz, Ar), 2.20 (t, 2H, *J* = 7.2 Hz, CH_2_), 1.54 (qui, 2H, *J* = 7.2 Hz, CH_2_), 1.29−1.34 (m, 6H, CH_2_), 0.88 (t, 3H, *J* = 6.8 Hz, CH_3_); ^13^C-NMR (100 MHz, DMSO-d_6_): δ 171.5, 163.9, 149.3, 138.2, 128.9, 123.6, 33.2, 31.0, 28.2, 25.0, 22.0, 13.9; IR (ATR) ν_max_: 3194 (N-H), 3030, 2960, 2926, 2854, 1609 (N-H), 1592 (N-H), 1509 (N-O), 1349 (C-N), 1212, 867, 847 (N-O), 716 cm^−1^; HRMS calcd for (C_14_H_19_N_3_O_4_+H^+^): 294.1454; found: 294.1458. UV-Vis (CH_3_OH) λ_max_ (logε) 214 (3.98), 266 (4.09) nm.
*4-Nitro-N′-hexadecanoylbenzohydrazide* (**5c**). The product was obtained as a white solid (7.10 g, 85%); mp 160–161 °C). IR (ATR) ν_max_: 3211 (N-H), 2917, 2848, 1609 (N-H), 1592 (N-H), 1574, 1522 (N-O), 1464, 1349 (C-N), 1210, 872, 846 (N-O), 719 cm^−1^; HRMS calcd for (C_23_H_36_N_3_O_4_+H^+^): 420.2862; found: 420.2843.
*N′-Propionyl-4′-nitrobenzohydrazide* (**5d**). The product was obtained as a white solid (1.80 g, 37%); mp 192–193 °C (203–205 °C [52]). UV-Vis (CH_3_OH) λ_max_ (logε) 262 (4.11) nm.
*4-Amino-N′-heptanoylbenzohydrazide* (**5e**). The product was obtained as a white solid (3.90 g, 75%); mp 188–189 °C. ^1^H-NMR (400 MHz, DMSO-d_6_): δ 9.75 (s, 1H, NH), 9.60 (s, 1H, NH), 7.59 (d, 2H, *J* = 8.4 Hz, Ar), 6.53 (d, 2H, *J* = 8.4 Hz, Ar), 5.69 (s, 2H, NH_2_), 2.14 (t, 2H, *J* = 7.6 Hz, CH_2_), 1.51 (qui, 2H, *J* = 7.6 Hz, CH_2_), 1.21–1.33 (m, 6H, CH_2_), 0.87 (t, 3H, *J* = 6.8 Hz, CH_3_), ^13^C NMR (100 MHz, DMSO-d_6_): δ 171.6, 165.5, 152.1, 129.0, 119.0, 112.5, 33.3, 31.0, 28.2, 25.1, 22.0, 13.4; IR (ATR) ν_max_: 3463 (N-H), 3355 (N-H), 3209 (N-H), 2924, 1695, 1598 (N-H), 1486, 1307, 1180, 724 cm^−1^; HRMS calcd for (C_14_H_21_N_3_O_2_+H^+^): 264.1712; found: 264.1722. UV-Vis (CH_3_OH) λ_max_ (logε) 288 (4.38) nm.

#### 3.2.5. General Procedure for the Synthesis of 2-Alkyl-5-(4-nitrophenyl)-1,3,4-oxadiazoles (**6a**–**d**)

*N,N′*-Diacylhydrazine (**5a**–**d**, 0.01 mol), phosphorus pentaoxide (6.9 g), and dry toluene (70 mL) were refluxed in an oil bath until the *N,N′*-diacylhydrazine was fully consumed (monitored by TLC, 1–3 h). Then, the solvent was evaporated on a rotary evaporator, and the resulting precipitate was dissolved in water (70 mL) and extracted with ethyl acetate or chloroform (3 × 30 mL). The organic phase was dried over anhydrous MgSO_4_, and the solvent was evaporated on a rotary evaporator. The crude products (**6a**–**d**) were used without additional purification.

*2-Butyl-5-(4-nitrophenyl)-1,3,4-oxadiazole* (**6a**). The product was obtained as a white solid (1.98 g, 80%); mp 66–67 °C (130–131 °C [66]). ^1^H-NMR (400 MHz, CDCl_3_): δ 8.36 (d, 2H, *J* = 8.8 Hz, Ar), 8.23 (d, 2H, *J* = 8.8 Hz, Ar), 2.98 (t, 2H, *J* = 7.6 Hz, CH_2_), 1.85 (qui, 2H, *J* = 7.6 Hz, CH_2_), 1.46 (sextet, 2H, *J* = 7.2 Hz, CH_2_), 1.00 (t, 3H, *J* = 7.2 Hz, CH_3_); ^13^C-NMR (100 MHz, CDCl_3_): δ 168.1, 163.0, 149.4, 129.6, 127.6, 124.0, 28.5, 25.2, 22.1, 13.5; IR (ATR) ν_max_: 3109, 2933, 2872, 1738 (N=C), 1607, 1562, 1517 (N-O), 1348, 1233 (-O-), 1106, 867 (N-O), 714cm^−1^; HRMS calcd for (C_12_H_13_N_3_O_3_+H^+^): 248.1035; found: 248.1007; UV-Vis (CH_3_OH) λ_max_ (logε) 204 (4.68), 286 (4.40) nm.
*2-Hexyl-5-(4-nitrophenyl)-1,3,4-oxadiazole* (**6b**). The product was obtained as a white solid (2.26 g, 82%); mp 60–61 °C. ^1^H-NMR (400 MHz, DMSO-d_6_): δ, 8.41 (d, 2H, *J* = 8.8 Hz, Ar), 8.23 (d, 2H, *J* = 8.8 Hz, Ar), 2.97 (t, 2H, *J* = 7.6 Hz, CH_2_), 1.76 (qui, 2H, *J* = 7.6 Hz, CH_2_), 1.30−1.38 (m, 6H, CH_2_), 0.87 (t, 3H, *J* = 7.6 Hz, CH_3_); ^13^C-NMR (100 MHz, DMSO-d_6_): δ 167.9, 162.5, 149.0, 129.0, 127.7, 124.6, 30.7, 27.9, 25.7, 24.6, 21.9, 13.8; IR (ATR) ν_max_: 3110, 2916, 2851, 1739 (N=C), 1606, 1567, 1518 (N-O), 1338, 1231 (-O-), 1106, 867 (N-O), 710 cm^−1^; HRMS calcd for (C_14_H_17_N_3_O_3_+H^+^): 276.1348; found: 276.1351; UV-Vis (CH_3_OH) λ_max_ (logε) 218 (4.02), 288 (4.26) nm.
*2-Pentadecyl-5-(4-nitrophenyl)-1,3,4-oxadiazole* (**6c**). The product was obtained as a white solid (3.10 g, 78%); mp 89–90 °C). ^1^H-NMR (400 MHz, CDCl_3_): δ 8.36 (d, 2H, *J* = 8.8 Hz, Ar), 8.22 (d, 2H, *J* = 8.8 Hz, Ar), 2.96 (t, 2H, *J* = 7.2 Hz, CH_2_), 1.87 (qui, 2H, *J* = 7.2 Hz, CH_2_), 1.25–1.47 (m, 24H, CH_2_), 0.88 (t, 3H, *J* = 7.2 Hz, CH_3_); ^13^C-NMR (100 MHz, CDCl_3_): δ 168.2, 163.0, 149.4, 129.6, 127.6, 124.3, 31.9, 29.65, 29.64, 29.62, 29.61, 29.6, 29.5, 29.4, 29.3, 29.1, 29.0, 26.5, 25.5, 22.7, 14.1; IR (ATR) ν_max_: 3120, 2914, 2848, 1738 (N=C), 1607, 1567, 1519 (N-O), 1342, 1218 (-O-), 1107, 868 (N-O), 710 cm^−1^; HRMS calcd for (C_23_H_35_N_3_O_3_+H^+^): 402.2757; found: 402.2755. UV-Vis (CH_2_Cl_2_) λ_max_ (logε) 294 (4.25) nm.
*2-Ethyl-5-(4-nitrophenyl)-1,3,4-oxadiazole* (**6d**). The product was obtained as a white solid (2.10 g, 97%); mp 132–133 °C (131–135 °C [52]). UV-Vis (CH_3_OH) λ_max_ (logε) 286 (4.47) nm.

#### 3.2.6. General Procedure for the Synthesis of 4-(5-Alkyl-1,3,4-oxadiazol-2-yl)anilines (**7a**–**d**)

5-Alkyl-2-(4-nitrophenyl)-1,3,4-oxadiazole (**6a**–**d**, 0.01 mol), SnCl_2_∙2H_2_O (11.3 g, 0.05 mol), and ethanol (40 mL) were heated until a homogenous solution was achieved. The mixture was allowed to cool and a suspension of NaBH_4_ (0.5 g, 13 mmol) in ethanol (25 mL) was added dropwise. It was agitated at room temperature for 1–3 h (monitored by TLC). Then, the mixture was alkalized with 20% NaOH, the precipitate was removed by filtration under reduced pressure, and the filtrate was concentrated on a rotary evaporator. It was extracted with chloroform (3 × 25 mL), dried over MgSO_4_, filtered, and the solvent was evaporated, yielding the crude products (**7a**–**d**).

*4-(5-Butyl-1,3,4-oxadiazol-2-yl)aniline* (**7a**). The product was obtained as a brown solid (1.98 g, 91%); mp 103–104 °C (119–122 °C [52]). ^1^H-NMR (400 MHz, CDCl_3_): δ 7.80 (d, 2H, *J* = 8.4 Hz, Ar), 6.71 (d, 2H, *J* = 8.8 Hz, Ar), 4.06 (s, 2H, NH_2_), 2.88 (t, 2H, *J* = 7.6 Hz, CH_2_), 1.77 (qui, 2H, *J* = 7.2 Hz, CH_2_), 1.41 (sextet, 2H, *J* = 7.2 Hz, CH_2_), 0.97 (t, 3H, *J* = 7.2 Hz, CH_3_); ^13^C-NMR (100 MHz, CDCl_3_): δ 166.0, 165.0, 149.4, 128.4, 114.6, 112.4, 28.7, 25.1, 22.1, 13.6; IR (ATR) ν_max_: 3413 (N-H), 3328 (N-H), 3215, 2960, 2960, 2960, 1606 (N-H), 1497, 1315(C-N), 1173 (C-O), 827 cm^−1^; HRMS calcd for (C_12_H_15_N_3_O+H^+^): 218.1293; found: 218.1275; UV-Vis (CH_3_OH) λ_max_ (logε) 202 (4.13), 218 (4.01), 306 (4.36) nm.
*4-(5-Hexyl-1,3,4-oxadiazol-2-yl)aniline* (**7b**). The product was obtained as a brown solid (2.20 g, 83%); mp 109–110 °C. ^1^H-NMR (400 MHz, DMSO-d_6_): δ 7.61 (d, 2H, *J* = 8.4 Hz, Ar), 6.66 (d, 2H, *J* = 8.4 Hz, Ar), 5.87 (s, 2H, NH_2_), 2.85 (t, 2H, *J* = 7.2 Hz, CH_2_), 1.70 (qui, 2H, *J* = 7.2 Hz, CH_2_), 1.29−1.36 (m, 6H, CH_2_), 0.86 (t, 3H, *J* = 7.2 Hz, CH_3_); ^13^C-NMR (100 MHz, DMSO-d_6_): δ 165.6, 164.9, 152.5, 128.0, 114.0, 110.5, 31.2, 28.4, 26.3, 25.0, 22.3, 14.2; IR (ATR) ν_max_: 3446 (N-H), 3339 (N-H), 3219, 2940, 1738, 1588, 1497, 1319 (C-N), 1176 (C-O), 835 cm^−1^; HRMS calcd for (C_14_H_19_N_3_O+H^+^): 246.1606; found: 246.1623. UV-Vis (CH_3_OH) λ_max_ (logε) 302 (4.43) nm.
*4-(5-Pentadecyl-1,3,4-oxadiazol-2-yl)aniline* (**7c**). The product was obtained as a yellow solid (3.30 g, 88%); mp 103–104 °C). ^1^H-NMR (600 MHz, CDCl_3_): δ 7.81 (d, 2H, *J* = 8.4 Hz, Ar), 6.72 (d, 2H, *J* = 8.4 Hz, Ar), 4.04 (s, 2H, NH_2_), 2.87 (t, 2H, *J* = 7.8 Hz, CH_2_), 1.82 (qui, 2H, *J* = 7.8 Hz, CH_2_), 1.20−1.42 (m, 24H, CH_2_), 0.88 (t, 3H, *J* = 7.8 Hz, CH_3_); ^13^C-NMR (151 MHz, CDCl_3_): δ 166.0, 165.0, 149.4, 128.4, 114.6, 112.5, 31.9, 29.66, 29.65, 29.64, 29.62, 29.61, 29.6, 29.4, 29.3, 29.1, 29.0, 26.7, 25.4, 22.7, 14.1; IR (ATR) ν_max_: 3337 (N-H), 2916, 2849, 1738, 1607, 1557, 1500, 1378 (C-N), 1178 (C-O), 842 cm^−1^; HRMS calcd for (C_23_H_37_N_3_O+H^+^): 372.3015; found: 372.3009. UV-Vis (CH_2_Cl_2_) λ_max_ (logε) 292 (4.55) nm.
*4-(5-Ethyl-1,3,4-oxadiazol-2-yl)aniline* (**7d**). The product was obtained as a yellow solid (1.80 g, 96%); mp 140–141 °C (138–142 °C [52]). UV-Vis (CH_3_OH) λ_max_ (logε) 302 (4.48) nm.

#### 3.2.7. Reduction of 2-Hexyl-5-(4-nitrophenyl)-1,3,4-oxadiazole (**7b**) with Iron Filings in Concentrated HCl

5-Hexyl-2-(4-nitrophenyl)-1,3,4-oxadiazole (**6b**, 3.6 mmol), iron filings (1.23 g; 0.022 mol), and concentrated HCl (4 mL) were heated in ethanol (40 mL) under reflux for 2 h (monitored by TLC). The mixture was alkalized with 20% NaOH, the precipitate was removed by filtration under reduced pressure, and the filtrate was concentrated on a rotary evaporator. Then, it was extracted with chloroform (3 × 25 mL), dried over MgSO_4_, filtered, and the solvent was evaporated on a rotary evaporator, yielding crude product **5e** as a white solid (0.62 g, 65%); mp 188–189 °C.

#### 3.2.8. Reduction of 2-Hexyl-5-(4-nitrophenyl)-1,3,4-oxadiazole (**7b**) with Iron Filings in Glacial Acetic Acid

5-Hexyl-2-(4-nitrophenyl)-1,3,4-oxadiazole (**6b**, 3.6 mmol), iron filings (1.23 g, 0.022 mol), and glacial acetic acid (4 mL) were heated under reflux for 6 h (monitored by TLC). Then, the mixture was alkalized with 20% NaOH, the precipitate was removed by filtration under reduced pressure, and the filtrate was concentrated on a rotary evaporator. It was extracted with chloroform (3 × 25 mL), dried over MgSO_4_, filtered, and the solvent was evaporated on a rotary evaporator, yielding crude product **7b** as a brown solid (0.62 g, 70%); mp 109–110 °C.

#### 3.2.9. General Procedure for the Synthesis of 4-{[4-(5-Alkyl-1,3,4-oxadiazol-2-yl)phenyl]diazenyl} phenol (**8a**–**b**, **8d**) 

The appropriate 5-alkyl-2-(4-aminophenyl)-1,3,4-oxadiazole (**7a**–**b**, **7d**, 4.61 mmol) was added to concentrated H_2_SO_4_ (7 mL), and the suspension was heated to 50 °C in a water bath and agitated until complete dissolution. Then, the mixture was cooled in an ice-salt bath to 0 °C, and NaNO_2_ solution (5.1 mmol, 0.35 g) in water (20 mL) was slowly added while maintaining the temperature at 0−5 °C. The mixture was stirred for 1 h, and urea was added to deactivate the excessive nitrous acid. Separately, phenol (4.61 mmol, 0.43 g) was dissolved in 20% NaOH (20 mL) and added dropwise to the diazonium salt solution. The mixture was agitated for 1 h at 0−5 °C. It was neutralized using 20% NaOH solution. The colored solid was washed with water (2 × 20 mL) and air-dried. The crude product (**8a–b**, **8d**) was additionally purified by column chromatography on silica gel using the appropriate mobile phase.

*4-{[4-(5-Butyl-1,3,4-oxadiazol-2-yl)phenyl]diazenyl}phenol* (**8a**). The product was obtained as a dark brown solid (1.19 g, 80%). It was purified by column chromatography (MeOH:CHCl_3_, 1:4, *v*/*v*); R_f_ = 0.71; mp 95–98 °C. ^1^H-NMR (400 MHz, DMSO-d_6_): δ 10.46 (s, 1H, OH), 8.14 (d, 2H, *J* = 8.4 Hz, Ar), 7.97 (d, 2H, *J* = 8.4 Hz, Ar), 7.85 (d, 2H, *J* = 8.8 Hz, Ar), 6.97 (d, 2H, *J* = 8.8 Hz, Ar), 2.95 (t, 2H, *J* = 7.2 Hz, CH_2_), 1.77 (qui, 2H, *J* = 7.2 Hz, CH_2_), 1.41 (sextet, 2H, *J* = 7.2 Hz, CH_2_), 0.94 (t, 3H, *J* = 7.2 Hz, CH_3_); ^13^C-NMR (100 MHz, DMSO-d_6_): δ 167.1, 163.4, 161.6, 153.6, 145.3, 129.3, 127.5, 125.3, 124.6, 116.1, 27.9, 24.3, 21.5, 13.4; IR (ATR) ν_max_: 3106 (O-H), 2958, 2873, 1586, 1466 (N=N), 1226 (C-OH), 1192, 1138, 849, 746 cm^−1^; HRMS calcd for (C_18_H_18_N_4_O_2_+H^+^): 323.1508; found: 323.1513; UV-Vis (CH_3_OH) λ_max_ (logε) 268 (4.03), 366 (4.26) nm.
*4-{[4-(5-Hexyl-1,3,4-oxadiazol-2-yl)phenyl]diazenyl}phenol* (**8b**). The product was obtained as a light brown solid (1.26 g, 78%). It was purified by column chromatography (Benzene:AcOEt, 1:3, *v*/*v*); R_f_ = 0.63; mp 125–126 °C. ^1^H-NMR (400 MHz, DMSO-d_6_): δ 10.45 (s, 1H, OH), 8.14 (d, 2H, *J* = 8.4 Hz, Ar), 7.98 (d, 2H, *J* = 8.4 Hz, Ar), 7.85 (d, 2H, *J* = 8.8 Hz, Ar), 6.97 (d, 2H, *J* = 8.8 Hz, Ar), 2.95 (t, 2H, *J* = 7.6 Hz, CH_2_), 1.78 (qui, 2H, *J* = 7.6 Hz, CH_2_), 1.30–1.39 (m, 6H, CH_2_), 0.87 (t, 3H, *J* = 6.8 Hz, CH_3_); ^13^C-NMR (100 MHz, DMSO-d_6_): δ 167.1, 163.4, 161.6, 153.6, 145.3, 129.3, 127.5, 125.3, 124.6, 116.1, 30.7, 28.0, 25.7, 24.6, 21.9, 13.8; IR (ATR) ν_max_: 3214 (O-H), 3054, 2924, 2854, 1586, 1464 (N=N), 1220 (C-OH), 1190, 1190, 1138, 850, 749 cm^−1^; HRMS calcd for (C_20_H_22_N_4_O_2_+H^+^): 351.1821; found: 351.1819; UV-Vis (CH_3_OH) λ_max_ (logε) 264 (3.80), 366 (4.17) nm.
*4-{[4-(5-Ethyl-1,3,4-oxadiazol-2-yl)phenyl]diazenyl}phenol* (**8d**). The product was obtained as a brown solid (1.01 g, 75%). It was purified by column chromatography (Benzene: AcOEt, 1:3, *v*/*v*); R_f_ = 0,52; mp 185–188 °C. ^1^H-NMR (400 MHz, DMSO-d_6_): δ 10.46 (s, 1H, OH), 8.14 (d, 2H, *J* = 8.4 Hz, Ar), 7.97 (d, 2H, *J* = 8.4 Hz, Ar), 7.85 (d, 2H, *J* = 8.8 Hz, Ar), 6.97 (d, 2H, *J* = 8.8 Hz, Ar), 2.98 (q, 2H, *J* = 7.6 Hz, CH_2_), 1.35 (t, 3H, *J* = 7.6 Hz, CH_3_); ^13^C-NMR (100 MHz, DMSO-d_6_): δ 168.0, 163.4, 161.6, 153.6, 145.3, 129.3, 127.5, 125.3, 122.9, 116.0, 18.4, 10.4; IR (ATR) ν_max_: 3187 (O-H), 2985, 1683, 1593, 1472 (N=N), 1227 (C-OH), 1099, 844, 755 cm^−1^; HRMS calcd for (C_16_H_14_N_4_O_2_+H^+^): 295.1195; found: 295.1182; UV-Vis (CH_3_OH) λ_max_ (logε) 262 (3.65), 362 (3.80) nm.

#### 3.2.10. General Procedure for the Synthesis of 4-{[4-(5-Alkyl-1,3,4-oxadiazol-2-yl)phenyl]diazenyl}benzene-1,3-diol (**9a**–**b**, **9d**) 

The appropriate 5-alkyl-2-(4-aminophenyl)-1,3,4-oxadiazole (**7a**–**b**, **7d**, 4.61 mmol) was added to concentrated H_2_SO_4_ (7 mL), and the suspension was heated to 50 °C in a water bath and agitated until complete dissolution. Then, the mixture was cooled in an ice-salt bath to 0 °C, and NaNO_2_ solution (5.1 mmol, 0.35 g) in water (20 mL) was slowly added, maintaining the temperature at 0−5 °C. The mixture was stirred for 1 h, and urea was added to deactivate the excessive nitrous acid. Separately, resorcinol (4.61 mmol, 0.51 g) was dissolved in 20% NaOH (20 mL) and added dropwise to the diazonium salt solution. The mixture was agitated for 1 h at 0−5 °C. It was neutralized using 20% NaOH solution. The colored solid was filtered under reduced pressure, washed with water (2 × 20 mL), and air-dried. The crude product (**9a**–**b**, **9d**) was purified by column chromatography on silica gel using the appropriate mobile phase.

*4-{[4-(5-Butyl-1,3,4-oxadiazol-2-yl)phenyl]diazenyl}benzene-1,3-diol* (**9a**). The product was obtained as a brown solid (1.45 g, 93%). It was purified by column chromatography (AcOEt:Hexane, 2:1, *v*/*v*); R_f_ = 0.42; mp 150–154 °C. ^1^H-NMR (400 MHz, DMSO-d_6_): δ 12.23 (s, 1H, OH), 10.69 (s, 1H, OH), 8.07 (d, 2H, *J* = 8.4 Hz, Ar), 7.99 (d, 2H, *J* = 8.4 Hz, Ar), 7.66 (d, 1H, *J* = 8.8 Hz, Ar), 6.47 (d, 1H, *J* = 8.8 Hz, Ar), 6.35 (s, 1H, Ar), 2.91 (t, 2H, *J* = 7.2 Hz, CH_2_), 1.73 (qui, 2H, *J* = 7.2 Hz, CH_2_), 1.37 (sextet, 2H, *J* = 7.2 Hz, CH_2_), 0.90 (t, 3H, *J* = 7.2 Hz, CH_3_); ^13^C-NMR (100 MHz, DMSO-d_6_): δ 167.5, 164.4, 163.9, 157.8, 152.9, 133.2, 129.5, 128.0, 124.5, 123.0, 110.0, 103.5, 28.3, 24.7, 22.00, 13.9; IR (ATR) ν_max_: 2956 (O-H), 1601, 1472 (N=N), 1232 (C-OH), 737 cm^−1^; HRMS calcd for (C_18_H_18_N_4_O_3_+H^+^): 339.1457; found: 339.1454; UV-Vis (CH_3_OH) λ_max_ (logε) 262 (4.00), 400 (4.28) nm.
*4-{[4-(5-Hexyl-1,3,4-oxadiazol-2-yl)phenyl]diazenyl}benzene-1,3-diol* (**9b**). The product was obtained as a red solid (1.54 g, 91%). It was purified by column chromatography (Benzene: AcOEt, 1:3, *v*/*v*); R_f_ = 0.55; mp 179–180 °C. ^1^H-NMR (400 MHz, DMSO-d_6_): δ 12.28 (s, 1H, OH), 10.72 (s, 1H, OH), 8.11 (d, 2H, *J* = 8.8 Hz, Ar), 8.02 (d, 2H, *J* = 8.8 Hz, Ar), 7.69 (d, 1H, *J* = 8.4 Hz, Ar), 6.51 (d, 1H, *J* = 8.4 Hz, Ar), 6.38 (d, 1H, *J* = 2.4 Hz, Ar), 2.94 (t, 2H, *J* = 7.2 Hz, CH_2_), 1.77 (qui, 2H, *J* = 7.2 Hz, CH_2_), 1.29–1.39 (m, 6H, CH_2_), 0.87 (t, 3H, *J* = 7.2 Hz, CH_3_); ^13^C-NMR (100 MHz, DMSO-d_6_): δ 167.0, 163.9, 163.4, 157.3, 152.4, 132.8, 129.1, 127.5, 124.1, 122.5, 109.6, 103.0, 30.7, 28.0, 25.7, 24.6, 21.9, 13.8; IR (ATR) ν_max_: 3094 (O-H), 2951, 2927, 2853, 1591, 1472 (N=N), 1317, 1228 (C-OH), 846, 744 cm^−1^; HRMS calcd for (C_20_H_22_N_4_O_3_+H^+^): 367.1770; found: 367.1747; UV-Vis (CH_3_OH) λ_max_ (logε) 264 (4.19), 402 (4.49) nm.
*4-{[4-(5-Ethyl-1,3,4-oxadiazol-2-yl)phenyl]diazenyl}benzene-1,3-diol* (**9d**). The product was obtained as a thick, dark red solid (1.27 g, 90%). It was purified by column chromatography (Hexane:AcOEt, 1:2, *v*/*v*); R_f_ = 0.38; mp 210–214 °C. ^1^H-NMR (400 MHz, DMSO-d_6_): δ 12.24 (s, 1H, OH), 10.71 (s, 1H, OH), 8.12 (d, 2H, *J* = 8.4 Hz, Ar), 8.03 (d, 2H, *J* = 8.4 Hz, Ar), 7.70 (d, 1H, *J* = 8.8 Hz, Ar), 6.51 (d, 1H, *J* = 8.8 Hz, Ar), 6.39 (s, 1H, Ar), 2.94 (q, 2H, *J* = 7.6 Hz, CH_2_), 1.35 (t, 3H, *J* = 7.6 Hz, CH_3_); ^13^C-NMR (100 MHz, DMSO-d_6_): δ 167.4, 164.6, 163.9, 157.3, 152.3, 132.8, 128.95, 127.5, 124.1, 122.5, 109.6, 103.0, 18.4, 10.4; IR (ATR) ν_max_: 3340 (O-H), 1602, 1477 (N=N), 1242, 1235 (C-OH), 1108, 839 cm^−1^; HRMS calcd for (C_16_H_14_N_4_O_3_+H^+^): 311.1144; found: 311.1145; UV-Vis (CH_3_OH) λ_max_ (logε) 262 (3.35), 398 (3.68) nm.

#### 3.2.11. General Procedure for the Synthesis of 4-{[4-(5-Alkyl-1,3,4-oxadiazol-2-yl)phenyl]diazenyl}-N,N-dimethylaniline (**10a**–**b**, **10d**) 

The appropriate 5-alkyl-2-(4-aminophenyl)-1,3,4-oxadiazole (**7a**–**b**, **7d**, 4.61 mmol) was added to concentrated H_2_SO_4_ (7 mL), and the suspension was heated to 50 °C in a water bath and agitated until complete dissolution. Then, the mixture was cooled in an ice-salt bath to 0 °C, and NaNO_2_ solution (5.1 mmol, 0.35 g) in water (20 mL) was slowly added, maintaining the temperature at 0−5 °C. The mixture was stirred for 1 h, and urea was added to deactivate the excessive nitrous acid. Separately, *N,N*-dimethylaniline solution (4.61 mmol, 0.56 g) in ethanol (10 mL) was prepared and added dropwise to the agitated diazonium salt solution. The mixture was agitated for 1 h at 0−5 °C. It was neutralized by the addition of 20% NaOH solution. The colored solid was filtered under reduced pressure, washed with water (2 × 20 mL), and air-dried. The crude product (**10a**–**b**, **10d**) was additionally purified by column chromatography on silica gel with the appropriate mobile phase.

*4-{[4-(5-Butyl-1,3,4-oxadiazol-2-yl)phenyl]diazenyl}-N,N-dimethylaniline* (**10a**). The product was obtained as a dark green solid (1.43 g, 89%). It was purified by column chromatography (Hexane: AcOEt, 7:3, *v*/*v*) R_f_ = 0.22. mp 270–272 °C. ^1^H-NMR (400 MHz, DMSO-d_6_): δ 8.10 (d, 2H, *J* = 7.6 Hz, Ar), 7.92 (d, 2H, *J* = 6.8 Hz, Ar), 7.72 (d, 2H, *J* = 6.8 Hz, Ar), 6.85 (d, 2H, *J* = 7.6 Hz, Ar), 3.04 (s, 6H, CH_3_), 2.85 (t, 2H, *J* = 6.4 Hz, CH_2_), 1.75 (qui, 2H, *J* = 6.4 Hz, CH_2_), 1.41 (sextet, 2H, *J* = 6.4 Hz, CH_2_), 0.92 (t, 3H, *J* = 6.4 Hz, CH_3_); ^13^C-NMR (100 MHz, DMSO-d_6_): δ 167.0, 163.1, 154.1, 152.9, 142.3, 128.6, 127.7, 127.4, 125.1, 111.9, 48.4, 28.7, 24.3, 21.5, 13.4; IR (ATR) ν_max_: 2956 (CH_3_), 2931, 2871, 1681, 1600, 1507, 1446 (N=N), 1363, 1136 (C-N), 1008, 822, 751, 692 cm^−1^; HRMS calcd for (C_20_H_23_N_5_O+H^+^): 350.1981; found: 350.1985; UV-Vis (CH_3_OH) λ_max_ (logε) 254 (4.31), 432 (3.74) nm.
*4-{[4-(5-Hexyl-1,3,4-oxadiazol-2-yl)phenyl]diazenyl}-N,N-dimethylaniline* (**10b**). The product was obtained as a dark red liquid (1.46 g, 84%). It was purified by column chromatography (Benzene:AcOEt, 1:3, *v*/*v*); R_f_ = 0.62. ^1^H-NMR (400 MHz, DMSO-d_6_): δ 8.00 (d, 2H, *J* = 8.0 Hz, Ar), 7.59 (d, 2H, *J* = 7.2 Hz, Ar), 7.31 (d, 2H, *J* = 7.2 Hz, Ar), 6.70 (d, 2H, *J* = 8.0 Hz, Ar), 3.32 (s, 6H, CH_3_), 2.92 (t, 2H, *J* = 7.2 Hz, CH_2_), 1.76 (qui, 2H, *J* = 7.2 Hz, CH_2_), 1.25–1.36 (m, 6H, CH_2_), 0.87 (t, 3H, *J* = 7.6 Hz, CH_3_); ^13^C-NMR (100 MHz, DMSO-d_6_): δ 166.7, 163.2, 154.9, 150.4, 142.7, 128.8, 128.1, 126.3, 125.9, 112.3, 49.3, 30.7, 27.9, 25.7, 24.5, 21.9, 13.8; IR (ATR) ν_max_: 2927 (CH_3_), 2857, 1682, 1599, 1495 (N=N), 1362, 1133 (C-N), 1011, 843, 751, 691 cm^−1^; HRMS calcd for (C_22_H_27_N_5_O+H^+^): 378.2294; found: 378.2271; UV-Vis (CH_3_OH) λ_max_ (logε) 254 (4.26), 368 (3.62), 436 (3.49) nm.
*4-{[4-(5-Ethyl-1,3,4-oxadiazol-2-yl)phenyl]diazenyl}-N,N-dimethylaniline* (**10d**). The product was obtained as a dark red liquid (1.29 g, 87%). It was purified by column chromatography (Benzene:AcOEt, 1:3, *v*/*v*); R_f_ = 0.25. ^1^H-NMR (400 MHz, DMSO-d_6_): δ 7.98 (d, 2H, *J* = 8.8 Hz, Ar), 7.64 (d, 2H, *J* = 8.4 Hz, Ar), 7.30 (d, 2H, *J* = 8.4 Hz, Ar), 6.66 (d, 2H, *J* = 8.8 Hz, Ar), 3.36 (s, 6H, CH_3_), 2.95 (q, 2H, *J* = 4.8 Hz, CH_2_), 1.32 (t, 3H, *J* = 4.8 Hz, CH_3_); ^13^C-NMR (100 MHz, DMSO-d_6_): δ 167.6, 163.3, 154.3, 151.1, 142.7, 128.1, 127.7, 126.3, 125.7, 111.9, 48.5, 19.7, 10.3; IR (ATR) ν_max_: 3224, 2974 (CH_3_), 1647, 1600, 1490 (N=N), 1363, 1170 (C-N), 1012, 820, 704 cm^−1^; HRMS calcd for (C_18_H_19_N_5_O+H^+^): 322.1668; found: 322.1622; UV-Vis (CH_3_OH) λ_max_ (logε) 266 (4.22), 424 (3.63) nm.

## 4. Conclusions

In summary, we developed a multistep reaction procedure for the preparation of three series of novel azo compounds, which were the derivatives of 2-alkyl-5-(4-(phenyldiazenyl)phenyl)-1,3,4-oxadiazole. The key intermediate *N′*-alkanecarbonyl-4-nitrobenzohydrazides, obtained from the reaction of the corresponding acid chlorides and hydrazides, were successfully cyclized using phosphorus pentoxide in toluene to 2-alkyl-5-(4-nitrophenyl)-1,3,4-oxadiazoles, in high yields of 80–90%. The subsequent selective reduction of the nitro group in 2-(4-nitrophenyl)-1,3,4-oxadiazole reactions required the selection of appropriate reagents and conditions. High yields, short reaction times, and easy isolation of products were achieved using the sodium borohydride and hydrated tin(II) chloride reducing system, which was not previously tested in the reduction of this type of 1,3,4-oxadiazole derivative. The synthesis of the titled azo dyes, containing a 5-(4-(phenyldiazenyl)phenyl)-1,3,4-oxadiazole scaffold substituted at position 2 of the heterocyclic core with alkyl groups, was conducted using a typical methodology of diazotization of the previously prepared 4-(5-alkyl-1,3,4-oxadiazol-2-yl)anilines, and then coupling with activated benzene derivatives: phenol, resorcinol and *N,N*-dimethylaniline. The final products were obtained in high yields (75–93%) and in a relatively short time. The newly synthesized compounds, demonstrating a broad color range spanning orange, red, violet, and even green, and good solubility in some polar solvents, may serve as potential candidates for the dye industry.

## Figures and Tables

**Table 1:** Derivatives of *N′*-alkanecarbonyl-4-nitrobenzohydrazide (**5a**–**d**) and 4-amino-*N′*-heptanoylbenzohydrazide (**5e**) derived from hydrazides and acid chlorides.

Entry	R ^1^	Acid Chloride	Product	Yield ^a^ [%]
1	C_4_H_9_	4-NO_2_-C_6_H_4_-COCl	**5a**	90
2	C_6_H_13_	4-NO_2_-C_6_H_4_-COCl	**5b**	88
3	C_15_H_31_	4-NO_2_-C_6_H_4_-COCl	**5c**	85
4	C_2_H_5_	4-NO_2_-C_6_H_4_-COCl	**5d**	37
5	C_6_H_13_	4-NH_2_-C_6_H_4_-COCl	**5e**	75

^a^ Isolated yields in respect to hydrazide **3**.

**Table 2:** Optimization of the cyclization reaction of *N′*-heptanoyl-4-nitrobenzohydrazide (**5b**) to afford 2-hexyl-5-(4-nitrophenyl)-1,3,4-oxadiazole (**6b**).

Entry	Cyclization Reagent	Solvent	Temperature ^a^ [°C]	Time [h]	Yield ^b^ [%]
1	POCl_3_	toluene	80	8	30
2	POCl_3_	toluene	reflux	8	40
3	POCl_3_	-	reflux	24	47
4	SOCl_2_	toluene	80	8	55
5	SOCl_2_	-	reflux	8	70
6	SOCl_2_	-	reflux	24	78
7	P_2_O_5_	-	reflux	1	65
8	P_2_O_5_	toluene	reflux	1	70
9	P_2_O_5_	toluene	reflux	1.5	80

^a^ Conventional heating in oil bath. ^b^ Isolated yields of **6b** in respect to compound **5b**.

**Table 3:** 2-Alkyl-5-(4-nitrophenyl)-1,3,4-oxadiazoles (**6a**–**d**) and their reduced products—4-(5-alkyl-1,3,4-oxadiazol-2-yl)anilines (**7a**–**d**).

Entry	R^1^	Product 6	Product 7
		Yield ^a^ [%]	Yield ^b^ [%]
1	C_4_H_9_ (**a**)	80	91
2	C_6_H_13_ (**b**)	82	83
3	C_15_H_31_ (**c**)	78	88
4	C_2_H_5_ (**d**)	97	96

^a^ Isolated yields in respect to compound **5**. ^b^ Isolated yields in respect to compound **6**.

**Table 4:** Three series of 2-alkyl-5-(4-(phenyldiazenyl)phenyl)-1,3,4-oxadiazoles (**8a**–**b**, **8d**; **9a**–**b**, **9d**; **10a**–**b**, **10d**) derived from 4-(5-alkyl-1,3,4-oxadiazol-2-yl)anilines (**7a**–**b**, **7d**).

Entry	Product	R^1^	X	Y	Yield ^a^ [%]
1	**8a**	C_4_H_9_	OH	H	80
2	**8b**	C_6_H_13_	OH	H	78
3	**8d**	C_2_H_5_	OH	H	75
4	**9a**	C_4_H_9_	OH	OH	93
5	**9b**	C_6_H_13_	OH	OH	91
6	**9d**	C_2_H_5_	OH	OH	90
7	**10a**	C_4_H_9_	N(CH_3_)_2_	H	89
8	**10b**	C_6_H_13_	N(CH_3_)_2_	H	84
9	**10d**	C_2_H_5_	N(CH_3_)_2_	H	87

^a^ Isolated yields with respect to the appropriate reagent (**7a–b**, **7d**).

## Data Availability

The data presented in this study are available on request from the corresponding author.

## Supplementary Materials

The following supporting information can be downloaded at https://www.mdpi.com/article/10.3390/molecules29184316/s1. Supporting information includes: Figure S1. ^1^H-NMR spectra (400 MHz, DMSO) of *Valeryl hydrazide* (**3a**); Figure S2. ^13^C-NMR spectra (100 MHz, DMSO) of *Valeryl hydrazide* (**3a**); Figure S3. ^1^H-NMR spectra (400 MHz, DMSO) of *Heptanehydrazide (***3b**); Figure S4. ^13^C-NMR spectra (100 MHz, DMSO) of *Heptanehydrazide (***3b**); Figure S5. ^1^H-NMR spectra (400 MHz, DMSO) of *4-Nitrobenzohydrazide* (**3d**); Figure S6. ^13^C-NMR spectra (100 MHz, DMSO) of *4-Nitrobenzohydrazide* (**3d**); Figure S7. IR spectra of *4-Nitro-N′-pentanoylbenzohydrazide* (**5a**); Figure S8. UV-Vis spectra (CH_3_OH) of *4-Nitro-N′-pentanoylbenzohydrazide* (**5a**); Figure S9. ^1^H-NMR spectra (400 MHz, DMSO) of *4-Nitro-N′-pentanoylbenzohydrazide* (**5a**); Figure S10. ^13^C-NMR spectra (100 MHz, DMSO) of *4-Nitro-N′-pentanoylbenzohydrazide* (**5a**); Figure S11. IR spectra of *N′-Heptanoyl-4-nitrobenzohydrazide* (**5b**); Figure S12. UV-Vis spectra (CH_3_OH) of *N′-Heptanoyl-4-nitrobenzohydrazide* (**5b**); Figure S13. ^1^H-NMR spectra (400 MHz, DMSO) of *N′-Heptanoyl-4-nitrobenzohydrazide* (**5b**); Figure S14. ^13^C-NMR spectra (100 MHz, DMSO) of *N′-Heptanoyl-4-nitrobenzohydrazide* (**5b**); Figure S15. IR spectra of *4-Nitro-N′-hexadecanoylbenzohydrazide* (**5c**); Figure S16. IR spectra of *N′-Propionyl-4′-nitrobenzohydrazide* (**5d**); Figure S17. UV-Vis spectra (CH_3_OH) of *N′-Propionyl-4′-nitrobenzohydrazide* (**5d**); Figure S18. ^1^H-NMR spectra (400 MHz, DMSO) of *N′-Propionyl-4′-nitrobenzohydrazide* (**5d**); Figure S19. ^13^C-NMR spectra (100 MHz, DMSO) of *N′-Propionyl-4′-nitrobenzohydrazide* (**5d**); Figure S20. IR spectra of *4-Amino-N′-heptanoylbenzohydrazide* (**5e**); Figure S21. UV-Vis spectra (CH_3_OH) of *4-Amino-N′-heptanoylbenzohydrazide* (**5e**); Figure S22. ^1^H-NMR spectra (400 MHz, DMSO) of *4-Amino-N′-heptanoylbenzohydrazide* (**5e**); Figure S23. ^13^C-NMR spectra (100 MHz, DMSO) of *4-Amino-N′-heptanoylbenzohydrazide* (**5e**); Figure S24. IR spectra of *2-Butyl-5-(4-nitrophenyl)-1,3,4-oxadiazole* (**6a**); Figure S25. UV-Vis spectra (CH_3_OH) of *2-Butyl-5-(4-nitrophenyl)-1,3,4-oxadiazole* (**6a**); Figure S26. ^1^H-NMR spectra (400 MHz, CDCl_3_) of *2-Butyl-5-(4-nitrophenyl)-1,3,4-oxadiazole* (**6a**); Figure S27. ^13^C-NMR spectra (100 MHz, CDCl_3_) of *2-Butyl-5-(4-nitrophenyl)-1,3,4-oxadiazole* (**6a**); Figure S28. IR spectra of *2-Hexyl-5-(4-nitrophenyl)-1,3,4-oxadiazole* (**6b**); Figure S29. UV-Vis spectra (CH_3_OH) of *2-Hexyl-5-(4-nitrophenyl)-1,3,4-oxadiazole* (**6b**); Figure S30. ^1^H-NMR spectra (400 MHz, DMSO) of *2-Hexyl-5-(4-nitrophenyl)-1,3,4-oxadiazole* (**6b**); Figure S31. ^13^C-NMR spectra (100 MHz, DMSO) of *2-Hexyl-5-(4-nitrophenyl)-1,3,4-oxadiazole* (**6b**); Figure S32. IR spectra of *2-Pentadecyl-5-(4-nitrophenyl)-1,3,4-oxadiazole* (**6c**); Figure S33. UV-Vis spectra (CH_2_Cl_2_) of *2-Pentadecyl-5-(4-nitrophenyl)-1,3,4-oxadiazole* (**6c**); Figure S34. ^1^H-NMR spectra (400 MHz, CDCl_3_) of *2-Pentadecyl-5-(4-nitrophenyl)-1,3,4-oxadiazole* (**6c**); Figure S35. ^13^C-NMR spectra (100 MHz, CDCl_3_) of *2-Pentadecyl-5-(4-nitrophenyl)-1,3,4-oxadiazole* (**6c**); Figure S36. IR spectra of *2-Ethyl-5-(4-nitrophenyl)-1,3,4-oxadiazole* (**6d**); Figure S37. UV-Vis spectra (CH_3_OH) of *2-Ethyl-5-(4-nitrophenyl)-1,3,4-oxadiazole* (**6d**); Figure S38. ^1^H-NMR spectra (400 MHz, CDCl_3_) of *2-Ethyl-5-(4-nitrophenyl)-1,3,4-oxadiazole* (**6d**); Figure S39. ^13^C-NMR spectra (100 MHz, CDCl_3_) of *2-Ethyl-5-(4-nitrophenyl)-1,3,4-oxadiazole* (**6d**); Figure S40. IR spectra of *4-(5-Butyl-1,3,4-oxadiazol-2-yl)aniline* (**7a**); Figure S41. UV-Vis spectra (CH_3_OH) of *4-(5-Butyl-1,3,4-oxadiazol-2-yl)aniline* (**7a**); Figure S42. ^1^H-NMR spectra (400 MHz, CDCl_3_) of *4-(5-Butyl-1,3,4-oxadiazol-2-yl)aniline* (**7a**); Figure S43. ^13^C-NMR spectra (100 MHz, CDCl_3_) of *4-(5-Butyl-1,3,4-oxadiazol-2-yl)aniline* (**7a**); Figure S44. IR spectra of *4-(5-Hexyl-1,3,4-oxadiazol-2-yl)aniline* (**7b**); Figure S45. UV-Vis spectra (CH_3_OH) of *4-(5-Hexyl-1,3,4-oxadiazol-2-yl)aniline* (**7b**); Figure S46. ^1^H-NMR spectra (400 MHz, DMSO) of *4-(5-Hexyl-1,3,4-oxadiazol-2-yl)aniline* (**7b**); Figure S47. ^13^C-NMR spectra (100 MHz, DMSO) of *4-(5-Hexyl-1,3,4-oxadiazol-2-yl)aniline* (**7b**); Figure S48. IR spectra of *4-(5-Pentadecyl-1,3,4-oxadiazol-2-yl)aniline* (**7c**); Figure S49. UV-Vis spectra (CH_2_Cl_2_) of *4-(5-Pentadecyl-1,3,4-oxadiazol-2-yl)aniline* (**7c**); Figure S50. ^1^H-NMR spectra (600 MHz, CDCl_3_) of *4-(5-Pentadecyl-1,3,4-oxadiazol-2-yl)aniline* (**7c**); Figure S51. ^13^C-NMR spectra (151 MHz, CDCl_3_) of *4-(5-Pentadecyl-1,3,4-oxadiazol-2-yl)aniline* (**7c**); Figure S52. IR spectra of *4-(5-Ethyl-1,3,4-oxadiazol-2-yl)aniline* (**7d**); Figure S53. UV-Vis spectra (CH_3_OH) of *4-(5-Ethyl-1,3,4-oxadiazol-2-yl)aniline* (**7d**); Figure S54. ^1^H-NMR spectra (400 MHz, CDCl_3_) of *4-(5-Ethyl-1,3,4-oxadiazol-2-yl)aniline* (**7d**); Figure S55. ^13^C-NMR spectra (100 MHz, CDCl_3_) of *4-(5-Ethyl-1,3,4-oxadiazol-2-yl)aniline* (**7d**); Figure S56. IR spectra of *4-{[4-(5-Butyl-1,3,4-oxadiazol-2-yl)phenyl]diazenyl}phenol* (**8a**); Figure S57. UV-Vis spectra (CH_3_OH) of *4-{[4-(5-Butyl-1,3,4-oxadiazol-2-yl)phenyl]diazenyl}phenol* (**8a**); Figure S58. ^1^H-NMR spectra (400 MHz, DMSO) of *4-{[4-(5-Butyl-1,3,4-oxadiazol-2-yl)phenyl]diazenyl}phenol* (**8a**); Figure S59. ^13^C-NMR spectra (100 MHz, DMSO) of *4-{[4-(5-Butyl-1,3,4-oxadiazol-2-yl)phenyl]diazenyl}phenol* (**8a**); Figure S60. IR spectra of *4-{[4-(5-Hexyl-1,3,4-oxadiazol-2-yl)phenyl]diazenyl}phenol* (**8b**); Figure S61. UV-Vis spectra (CH_3_OH) of *4-{[4-(5-Hexyl-1,3,4-oxadiazol-2-yl)phenyl]diazenyl}phenol* (**8b**); Figure S62. ^1^H-NMR spectra (400 MHz, DMSO) of *4-{[4-(5-Heksyl-1,3,4-oxadiazol-2-yl)phenyl]diazenyl}phenol* (**8b**); Figure S63. ^13^C-NMR spectra (100 MHz, DMSO) of *4-{[4-(5-Heksyl-1,3,4-oxadiazol-2-yl)phenyl]diazenyl}phenol* (**8b**); Figure S64. IR spectra of *4-{[4-(5-Ethyl-1,3,4-oxadiazol-2-yl)phenyl]diazenyl}phenol* (**8d**); Figure S65. UV-Vis spectra (CH_3_OH) of *4-{[4-(5-Ethyl-1,3,4-oxadiazol-2-yl)phenyl]diazenyl}phenol* (**8d**); Figure S66. ^1^H-NMR spectra (400 MHz, DMSO) of *4-{[4-(5-Ethyl-1,3,4-oxadiazol-2-yl)phenyl]diazenyl}phenol* (**8d**); Figure S67. ^13^C-NMR spectra (100 MHz, DMSO) of *4-{[4-(5-Ethyl-1,3,4-oxadiazol-2-yl)phenyl]diazenyl}phenol* (**8d**); Figure S68. IR spectra of *4-{[4-(5-Butyl-1,3,4-oxadiazol-2-yl)phenyl]diazenyl}benzene-1,3-diol* (**9a**); Figure S69. UV-Vis spectra (CH_3_OH) of *4-{[4-(5-Butyl-1,3,4-oxadiazol-2-yl)phenyl]diazenyl}benzene-1,3-diol* (**9a**); Figure S70. ^1^H-NMR spectra (400 MHz, DMSO) of *4-{[4-(5-Butyl-1,3,4-oxadiazol-2-yl)phenyl]diazenyl}benzene-1,3-diol* (**9a**); Figure S71. ^13^C-NMR spectra (100 MHz, DMSO) of *4-{[4-(5-Butyl-1,3,4-oxadiazol-2-yl)phenyl]diazenyl}benzene-1,3-diol* (**9a**); Figure S72. IR spectra of *4-{[4-(5-Hexyl-1,3,4-oxadiazol-2-yl)phenyl]diazenyl}benzene-1,3-diol* (**9b**); Figure S73. UV-Vis spectra (CH_3_OH) of *4-{[4-(5-Hexyl-1,3,4-oxadiazol-2-yl)phenyl]diazenyl}benzene-1,3-diol* (**9b**); Figure S74. ^1^H-NMR spectra (400 MHz, DMSO) of *4-{[4-(5-Heksyl-1,3,4-oxadiazol-2-yl)phenyl]diazenyl}benzene-1,3-diol* (**9b**); Figure S75. ^13^C-NMR spectra (100 MHz, DMSO) of *4-{[4-(5-Heksyl-1,3,4-oxadiazol-2-yl)phenyl]diazenyl}benzene-1,3-diol* (**9b**); Figure S76. IR spectra of *4-{[4-(5-Ethyl-1,3,4-oxadiazol-2-yl)phenyl]diazenyl}benzene-1,3-diol* (**9d**); Figure S77. UV-Vis spectra (CH_3_OH) of *4-{[4-(5-Ethyl-1,3,4-oxadiazol-2-yl)phenyl]diazenyl}benzene-1,3-diol* (**9d**); Figure S78. ^1^H-NMR spectra (400 MHz, DMSO) of *4-{[4-(5-Ethyl-1,3,4-oxadiazol-2-yl)phenyl]diazenyl}benzene-1,3-diol* (**9d**); Figure S79. ^13^C-NMR spectra (100 MHz, DMSO) of *4-{[4-(5-Ethyl-1,3,4-oxadiazol-2-yl)phenyl]diazenyl}benzene-1,3-diol* (**9d**); Figure S80. IR spectra of *4-{[4-(5-Butyl-1,3,4-oxadiazol-2-yl)phenyl]diazenyl}-N,N-dimethylaniline* (**10a**); Figure S81. UV-Vis spectra (CH_3_OH) of *4-{[4-(5-Butyl-1,3,4-oxadiazol-2-yl)phenyl]diazenyl}-N,N-dimethylaniline* (**10a**); Figure S82. ^1^H-NMR spectra (400 MHz, DMSO) of *4-{[4-(5-Butyl-1,3,4-oxadiazol-2-yl)phenyl]diazenyl}-N,N-dimethylaniline* (**10a**); Figure S83. ^13^C-NMR spectra (100 MHz, DMSO) of *4-{[4-(5-Butyl-1,3,4-oxadiazol-2-yl)phenyl]diazenyl}-N,N-dimethylaniline* (**10a**); Figure S84. IR spectra of *4-{[4-(5-Hexyl-1,3,4-oxadiazol-2-yl)phenyl]diazenyl}-N,N-dimethylaniline* (**10b**); Figure S85. UV-Vis spectra (CH_3_OH) of *4-{[4-(5-Hexyl-1,3,4-oxadiazol-2-yl)phenyl]diazenyl}-N,N-dimethylaniline* (**10b**); Figure S86. ^1^H-NMR spectra (400 MHz, DMSO) of *4-{[4-(5-Heksyl-1,3,4-oxadiazol-2-yl)phenyl]diazenyl}-N,N-dimethylaniline* (**10b**); Figure S87. ^13^C-NMR spectra (100 MHz, DMSO) of *4-{[4-(5-Heksyl-1,3,4-oxadiazol-2-yl)phenyl]diazenyl}-N,N-dimethylaniline* (**10b**); Figure S88. IR spectra of *4-{[4-(5-Ethyl-1,3,4-oxadiazol-2-yl)phenyl]diazenyl}-N,N-dimethylaniline* (**10d**); Figure S89. UV-Vis spectra (CH_3_OH) of *4-{[4-(5-Ethyl-1,3,4-oxadiazol-2-yl)phenyl]diazenyl}-N,N-dimethylaniline* (**10d**); Figure S90. ^1^H-NMR spectra (400 MHz, DMSO) of *4-{[4-(5-Ethyl-1,3,4-oxadiazol-2-yl)phenyl]diazenyl}-N,N-dimethylaniline* (**10d**); Figure S91. ^13^C-NMR spectra (100 MHz, DMSO) of *4-{[4-(5-Ethyl-1,3,4-oxadiazol-2-yl)phenyl]diazenyl}-N,N-dimethylaniline* (**10d**).
