# Supplementary material for: Synthesis and Characterization of Novel 2-Alkyl-1,3,4-Oxadiazoles Containing a Phenylazo Group
Source: Molecules. 2024 Sep 11;29(18):4316. doi: 10.3390/molecules29184316 (PMC11433806; doi:10.3390/molecules29184316)
Supplement: Supplementary file 1 [file molecules-29-04316-s001.zip › molecules-3191662-supplementary.pdf]

Supplementary materials

Synthesis and Characterization of Novel 2-Alkyl-1,3,4-Oxadiazoles containing Phenylazo Group

Sebastian Górecki and Agnieszka Kudelko*

Department of Chemical Organic Technology and Petrochemistry, The Silesian University of Technology, Krzywoustego 4, PL-44100 Gliwice, Poland

E-mail: Agnieszka.Kudelko@polsl.pl (A. Kudelko)

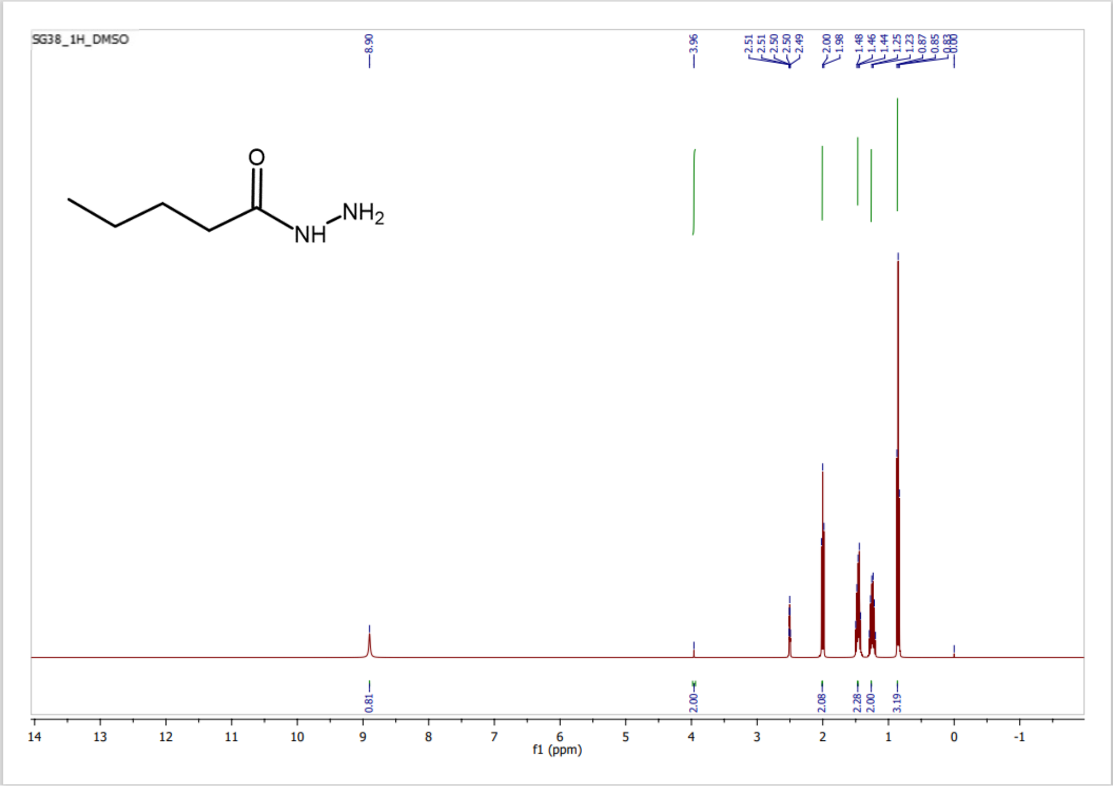

Figure S1. ^{1}H-NMR spectra (400 MHz, DMSO) of *Valeryl hydrazide* (**3a**)

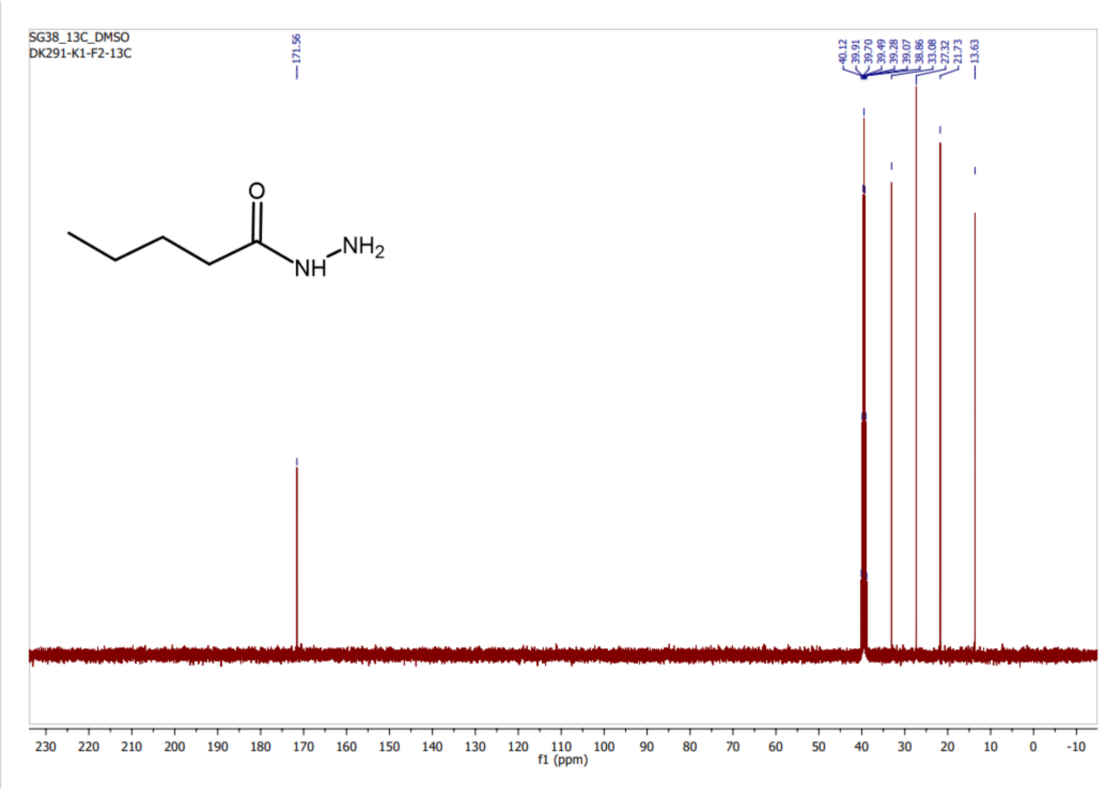

Figure S2. ^{13}C-NMR spectra (100 MHz, DMSO) of *Valeryl hydrazide* (**3a**)

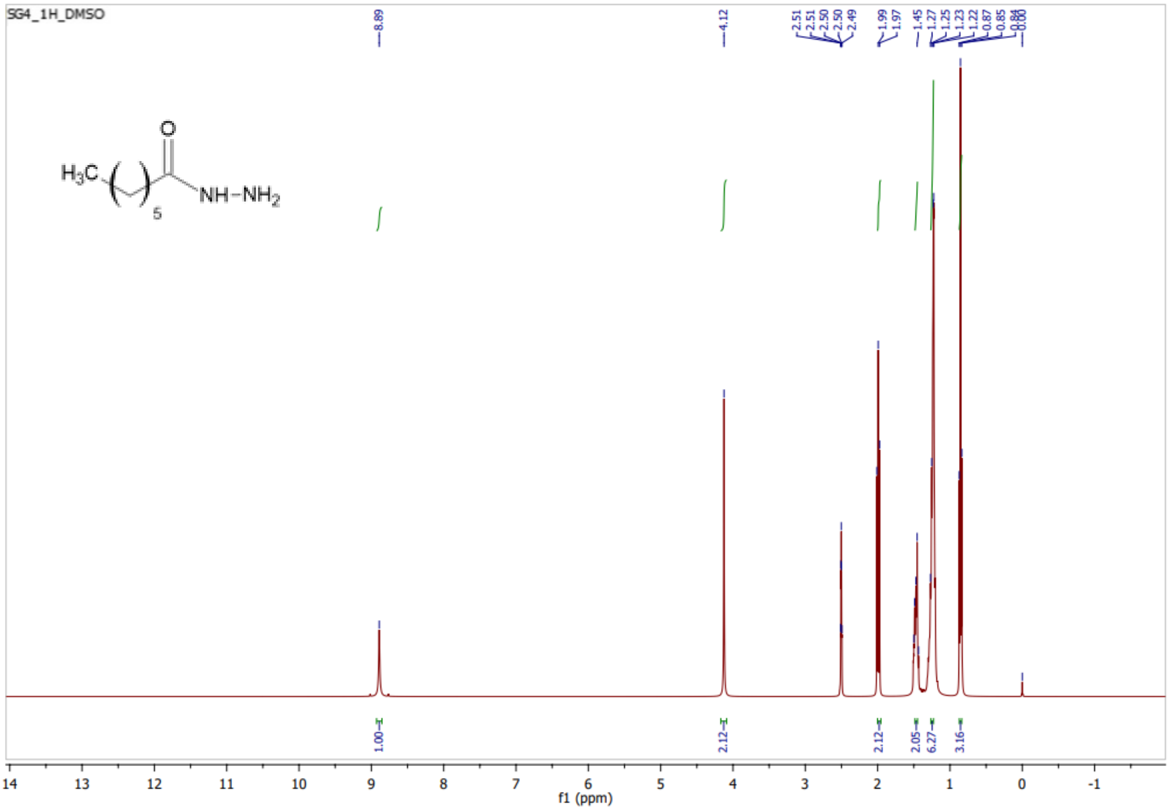

Figure S3. ^{1}H-NMR spectra (400 MHz, DMSO) of *Heptanehydrazide* (**3b**)

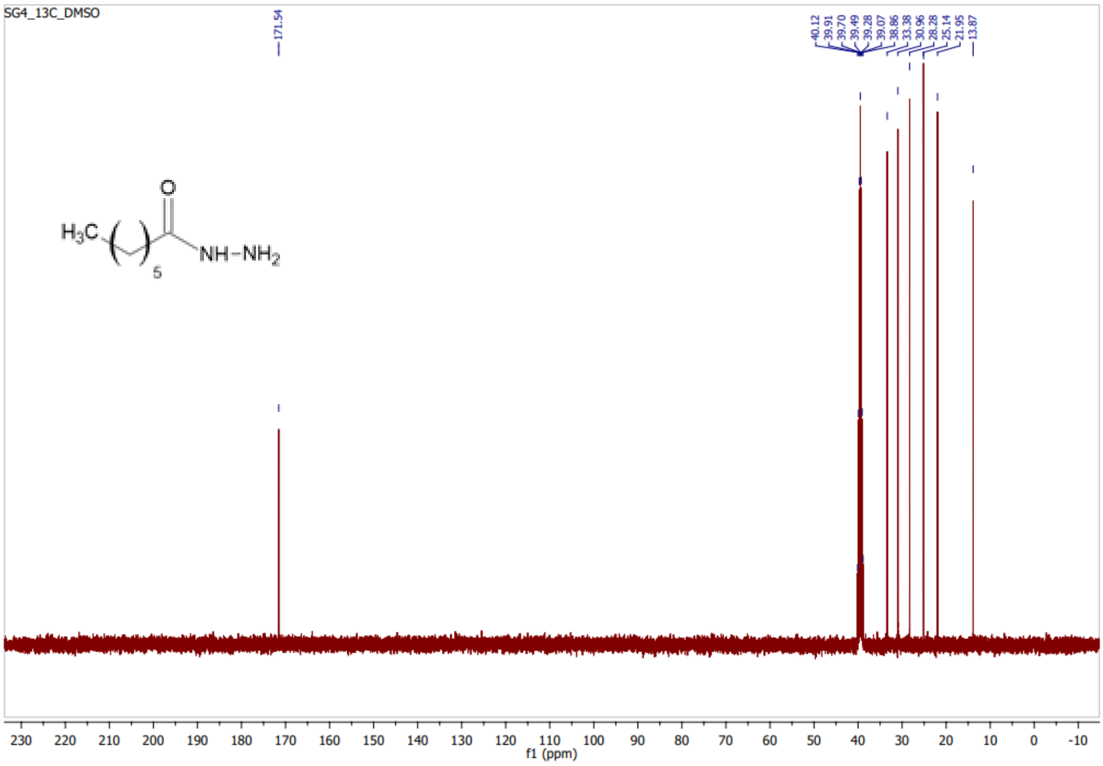

Figure S4. ^{13}C-NMR spectra (100 MHz, DMSO) of *Heptanehydrazide* (**3b**)

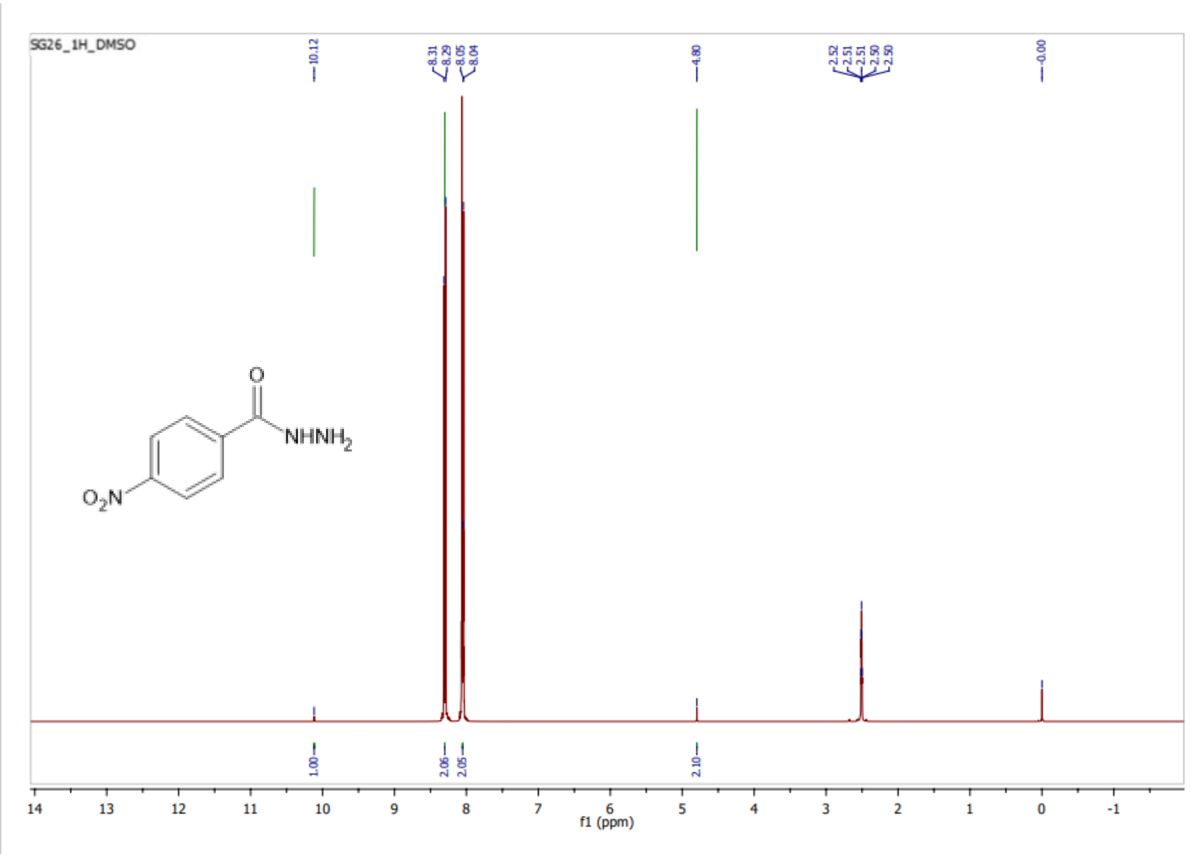

Figure S5. ^{1}H-NMR spectra (400 MHz, DMSO) of *4-Nitrobenzohydrazide* (**3d**)

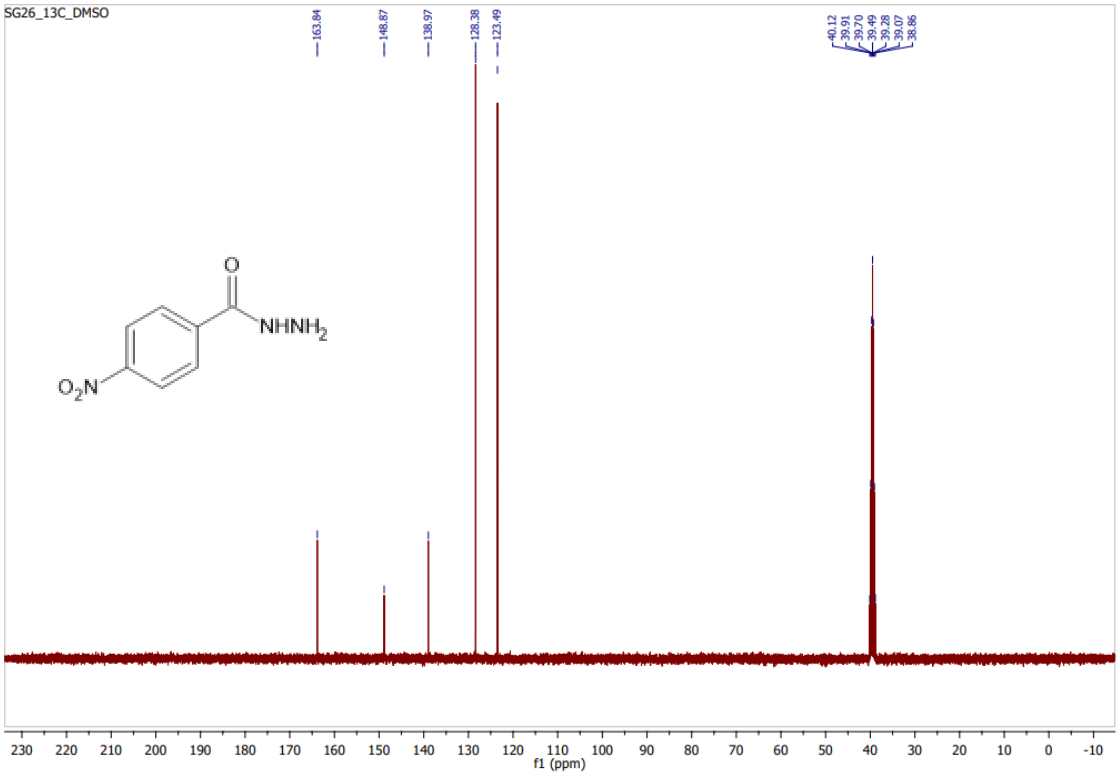

Figure S6. ^{13}C-NMR spectra (100 MHz, DMSO) of *4-Nitrobenzohydrazide* (**3d**)

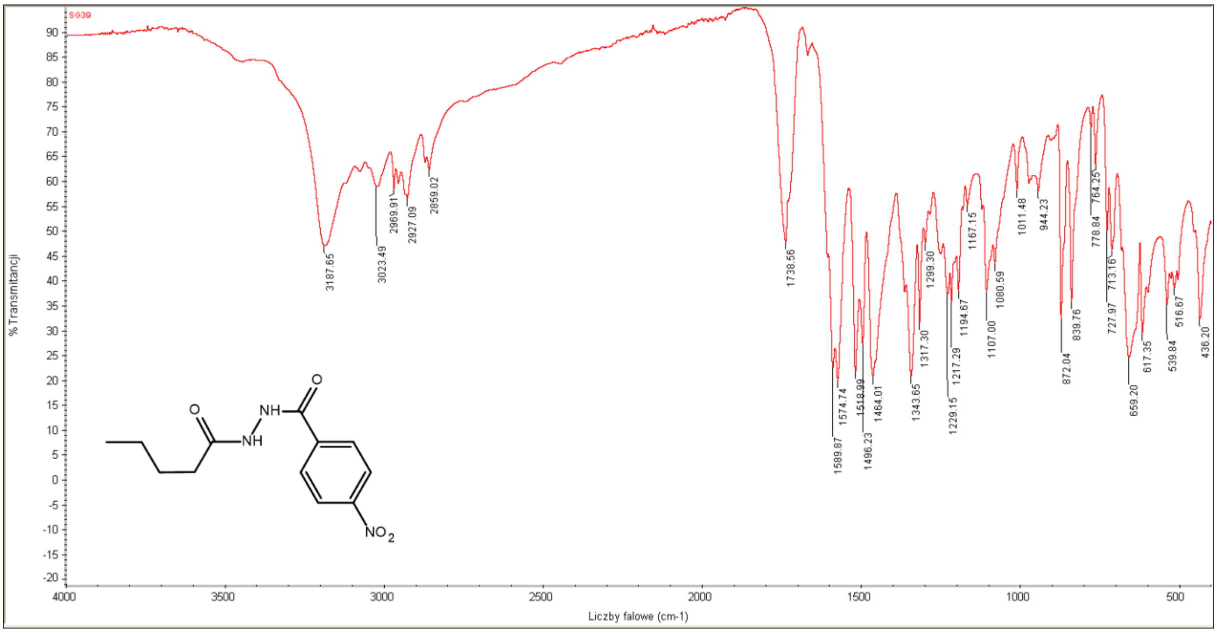

Figure S7. IR spectra of *4-Nitro-N′-pentanoylbenzohydrazide* (**5a**)

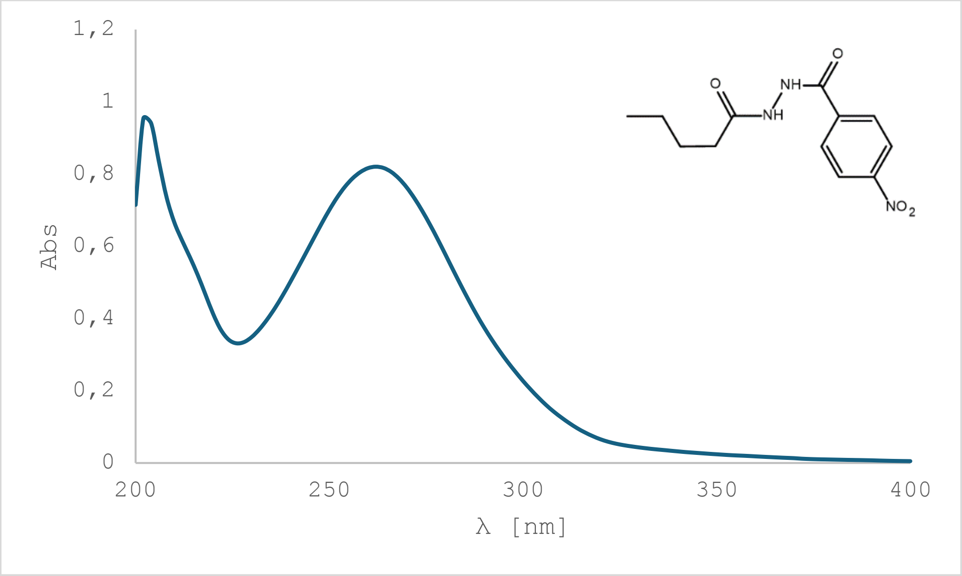

Figure S8. UV-Vis spectra (CH_3OH) of *4-Nitro-N′-pentanoylbenzohydrazide* (**5a**)

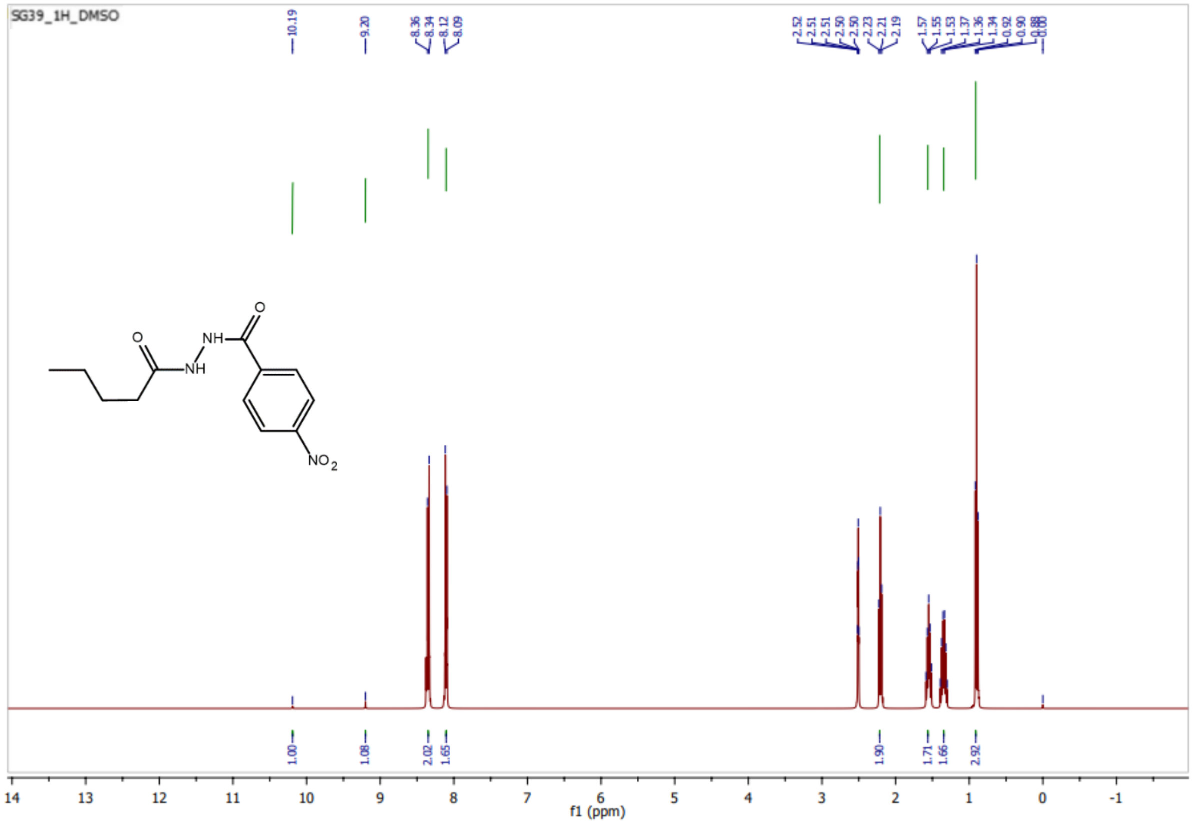

Figure S9. ^{1}H-NMR spectra (400 MHz, DMSO) of *4-Nitro-N′-pentanoylbenzohydrazide* (**5a**)

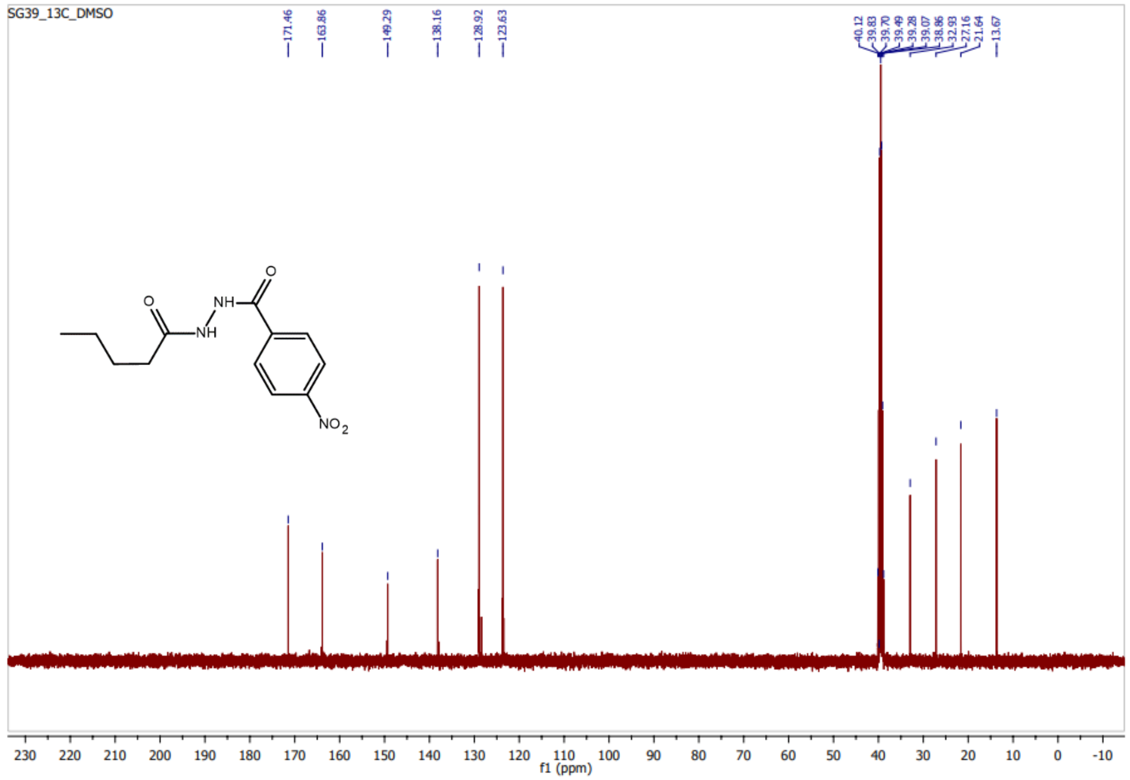

Figure S10. ^{13}C-NMR spectra (100 MHz, DMSO) of *4-Nitro-N′-pentanoylbenzohydrazide* (**5a**)

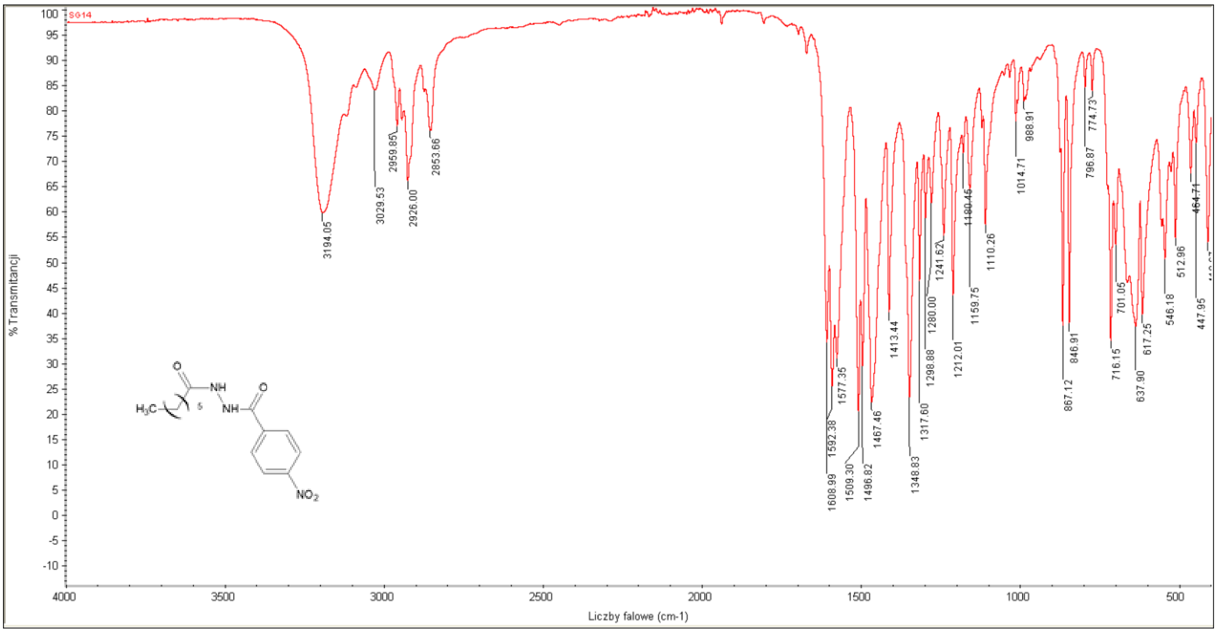

Figure S11. IR spectra of *N′-Heptanoyl-4-nitrobenzohydrazide* (**5b**)

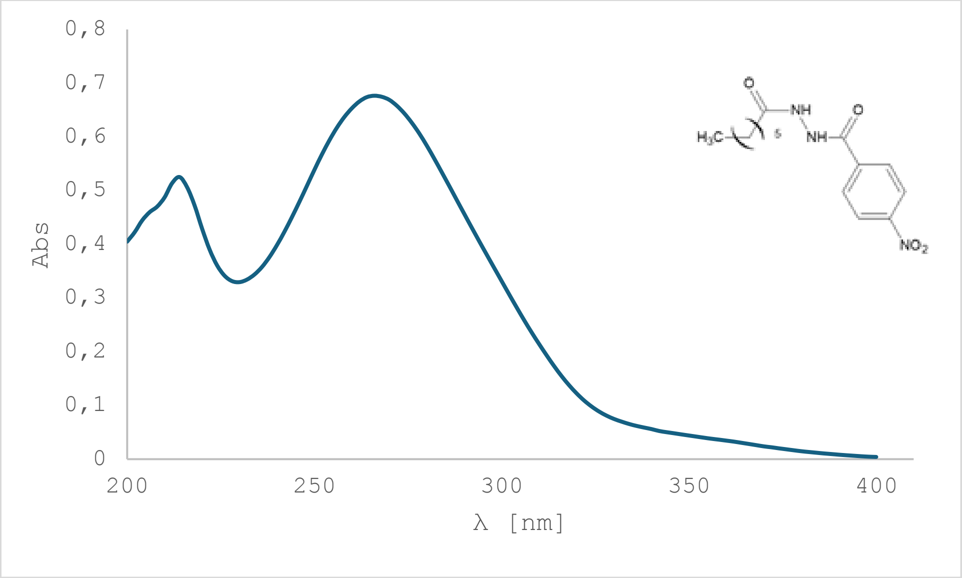

Figure S12. UV-Vis spectra (CH_3OH) of *N′-Heptanoyl-4-nitrobenzohydrazide* (**5b**)

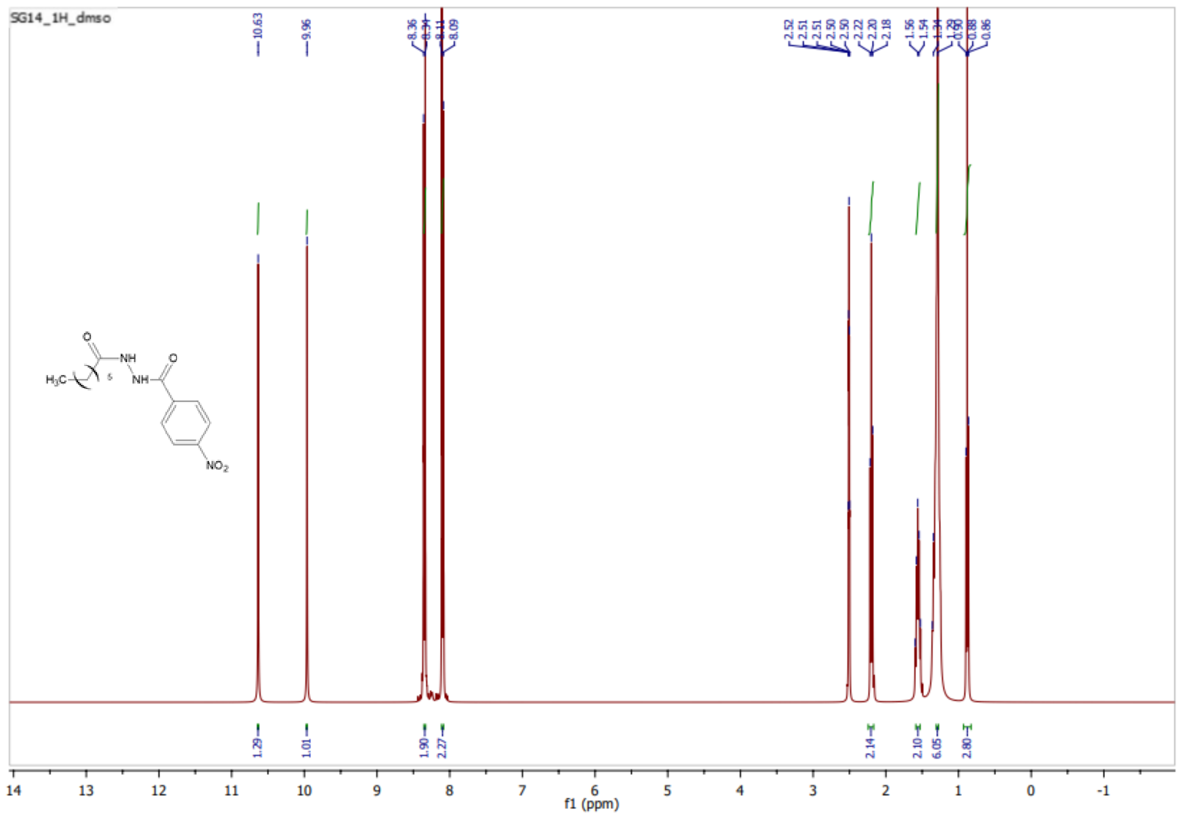

Figure S13. ^{1}H-NMR spectra (400 MHz, DMSO) of *N′-Heptanoyl-4-nitrobenzohydrazide* (**5b**)

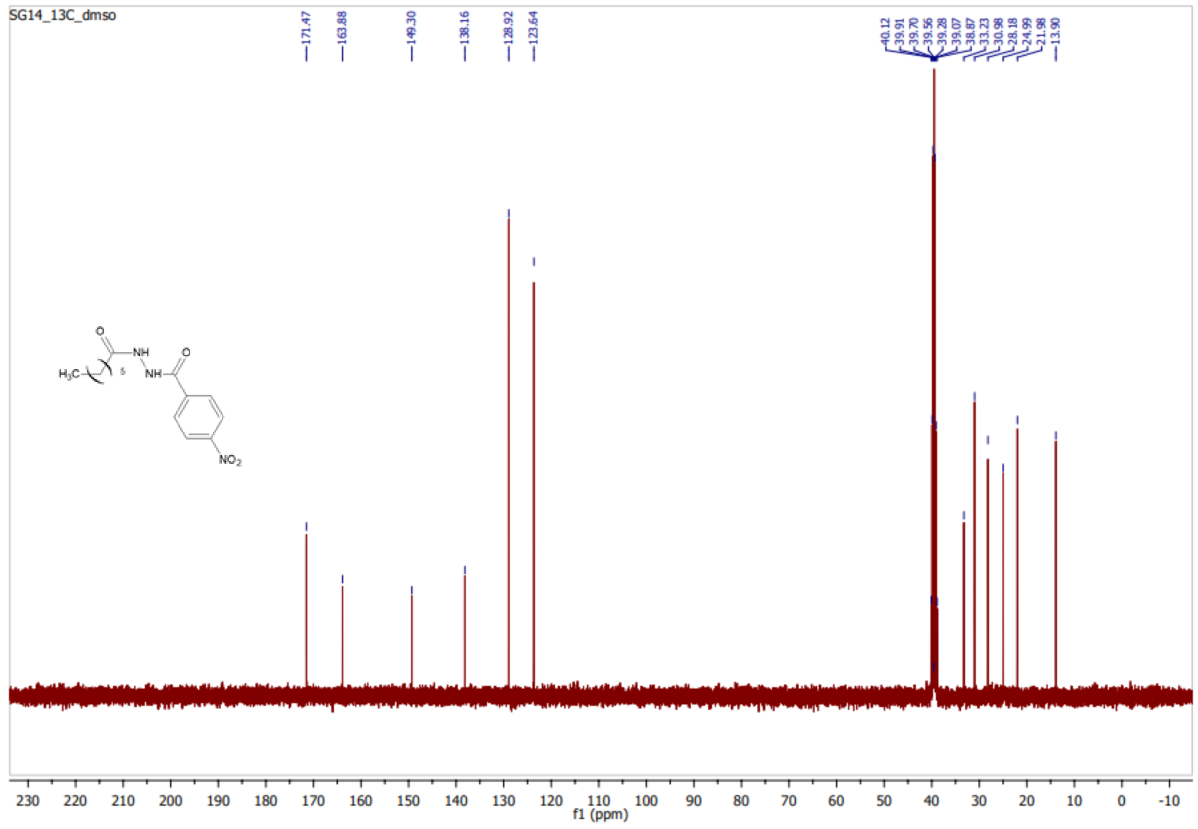

Figure S14. ^{13}C-NMR spectra (100 MHz, DMSO) of *N′-Heptanoyl-4-nitrobenzohydrazide* (**5b**)

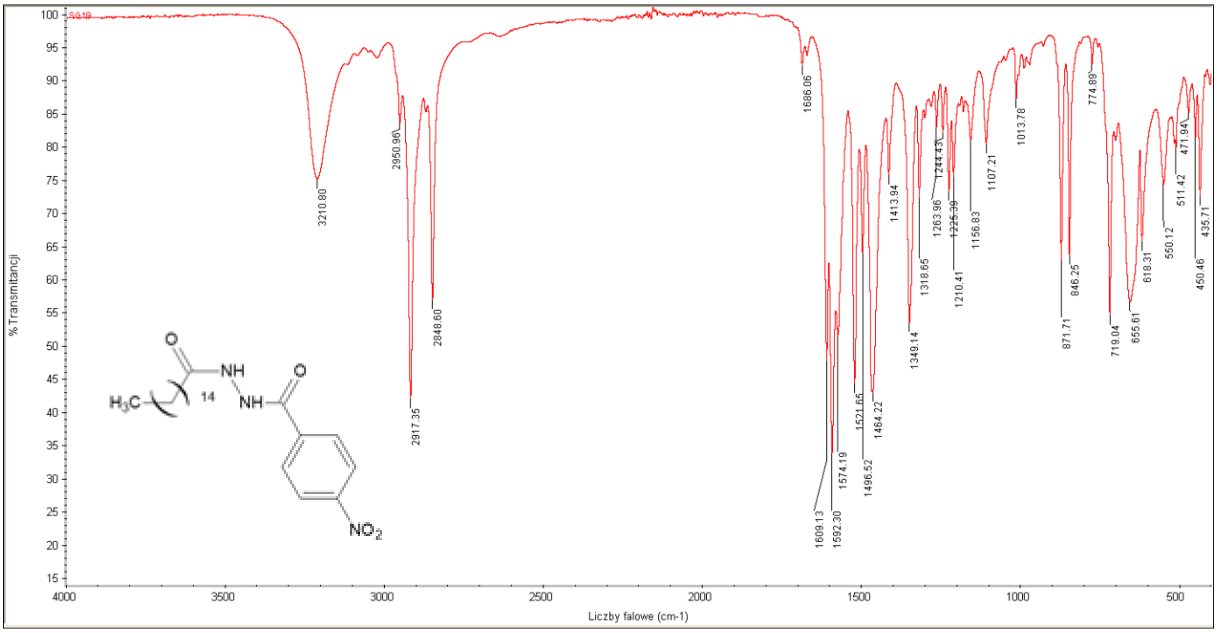

Figure S15. IR spectra of *4-Nitro-N′-hexadecanoylbenzohydrazide* (**5c**)

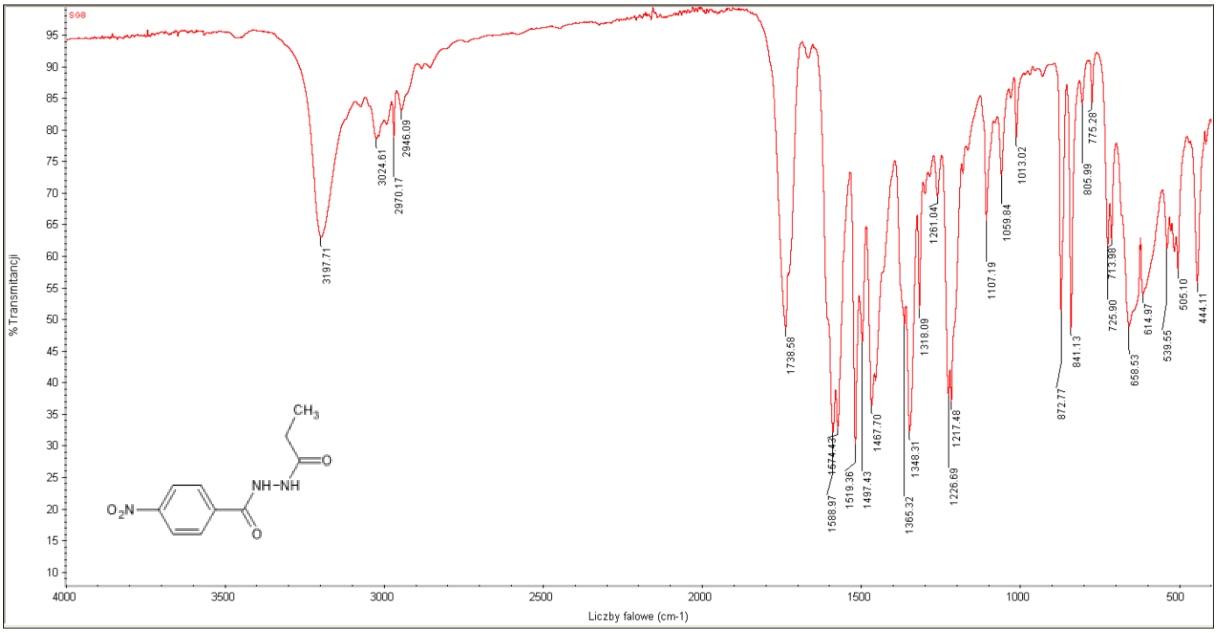

Figure S16. IR spectra of *N′-Propionyl-4′-nitrobenzohydrazide* (**5d**)

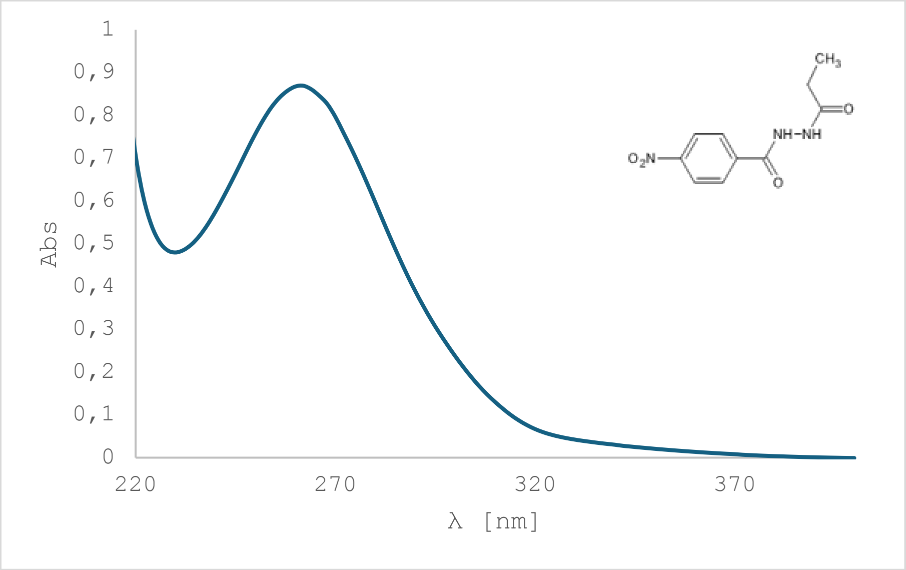

Figure S17. UV-Vis spectra (CH_3OH) of *N′-Propionyl-4′-nitrobenzohydrazide* (**5d**)

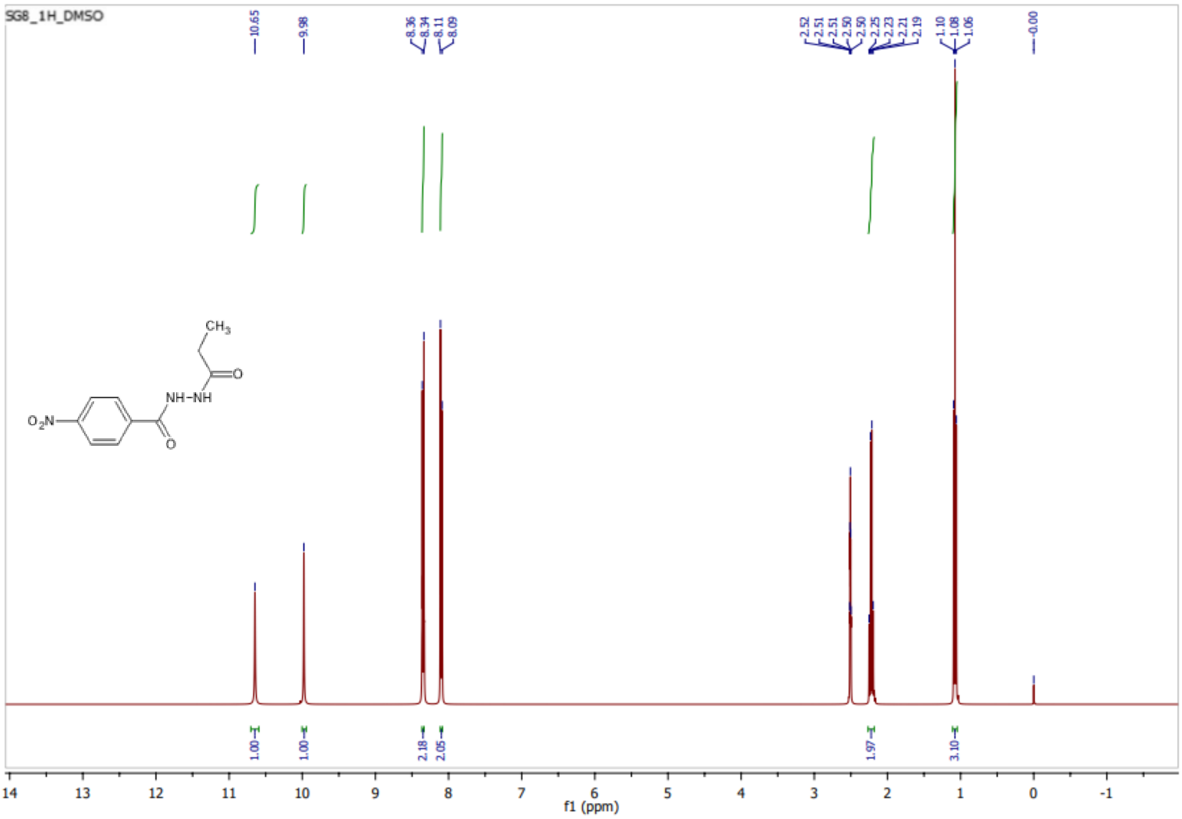

Figure S18. ^{1}H-NMR spectra (400 MHz, DMSO) of *N′-Propionyl-4′-nitrobenzohydrazide* (**5d**)

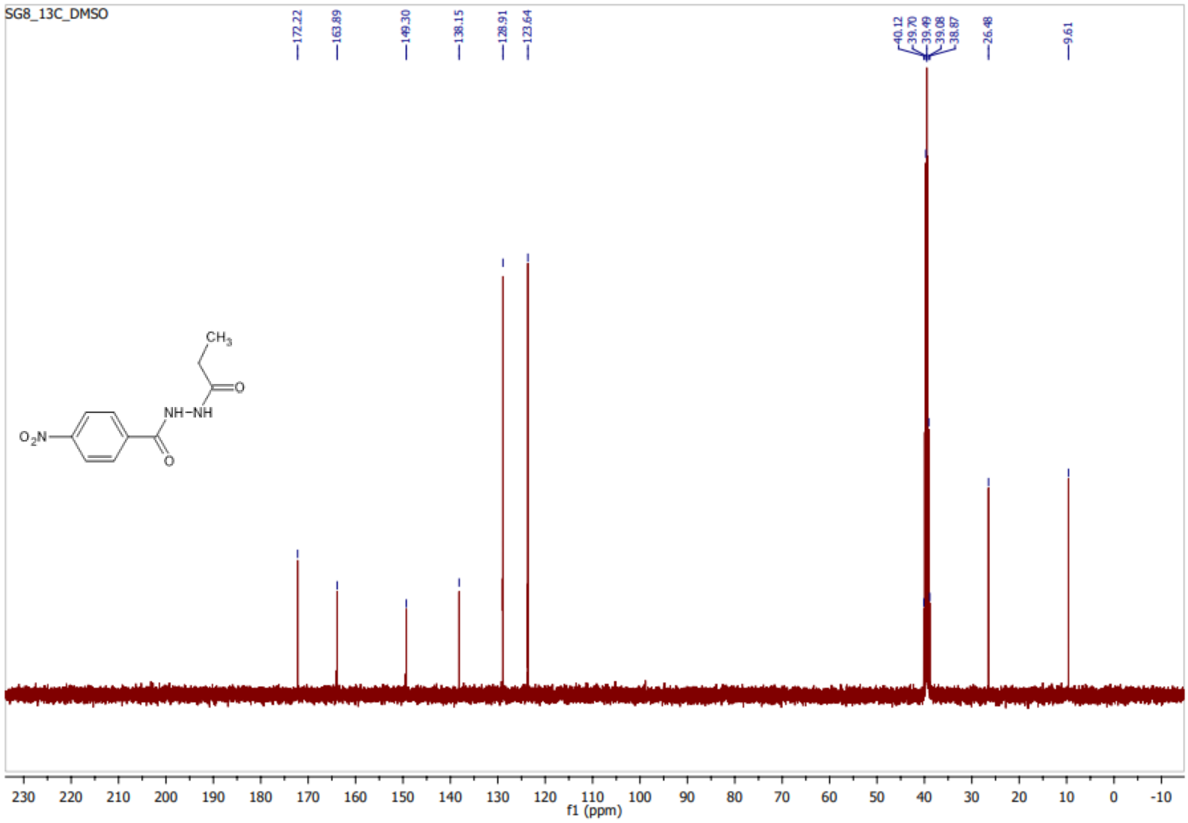

Figure S19. ^{13}C-NMR spectra (100 MHz, DMSO) of *N′-Propionyl-4′-nitrobenzohydrazide* (**5d**)

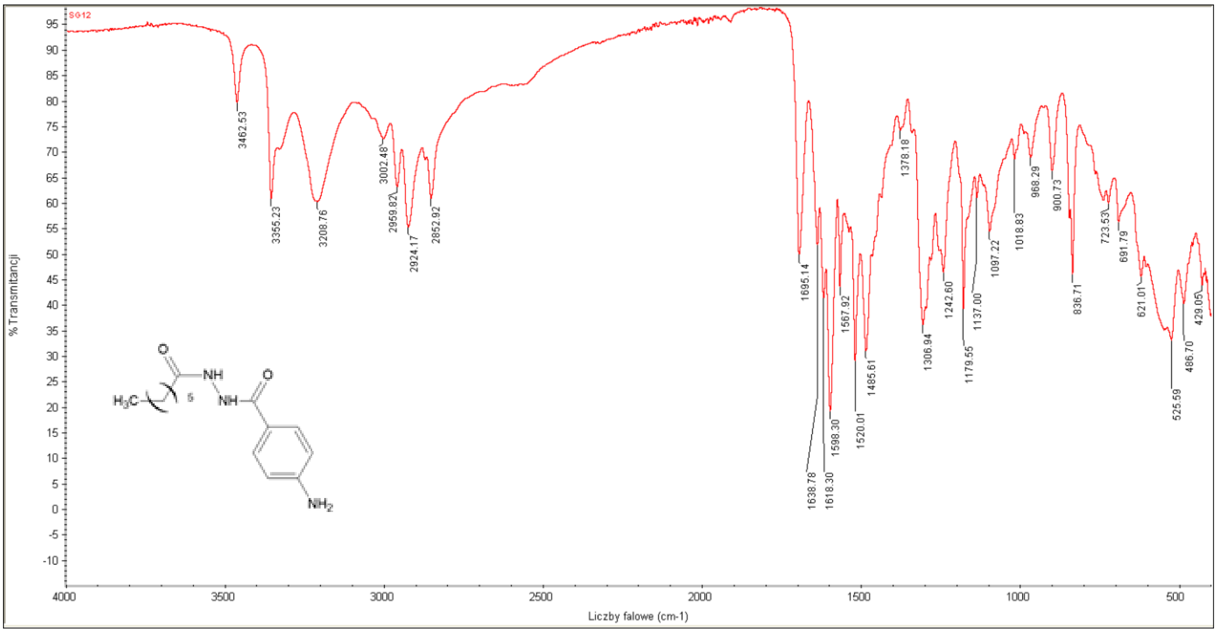

Figure S20. IR spectra of *4-Amino-N′-heptanoylbenzohydrazide* (**5e**)

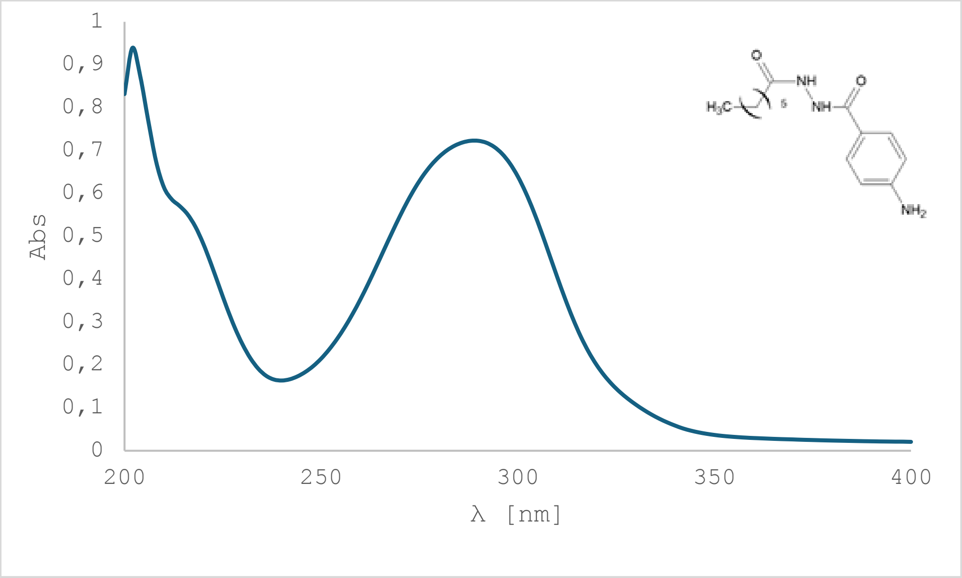

Figure S21. UV-Vis spectra (CH_3OH) of *4-Amino-N′-heptanoylbenzohydrazide* (**5e**)

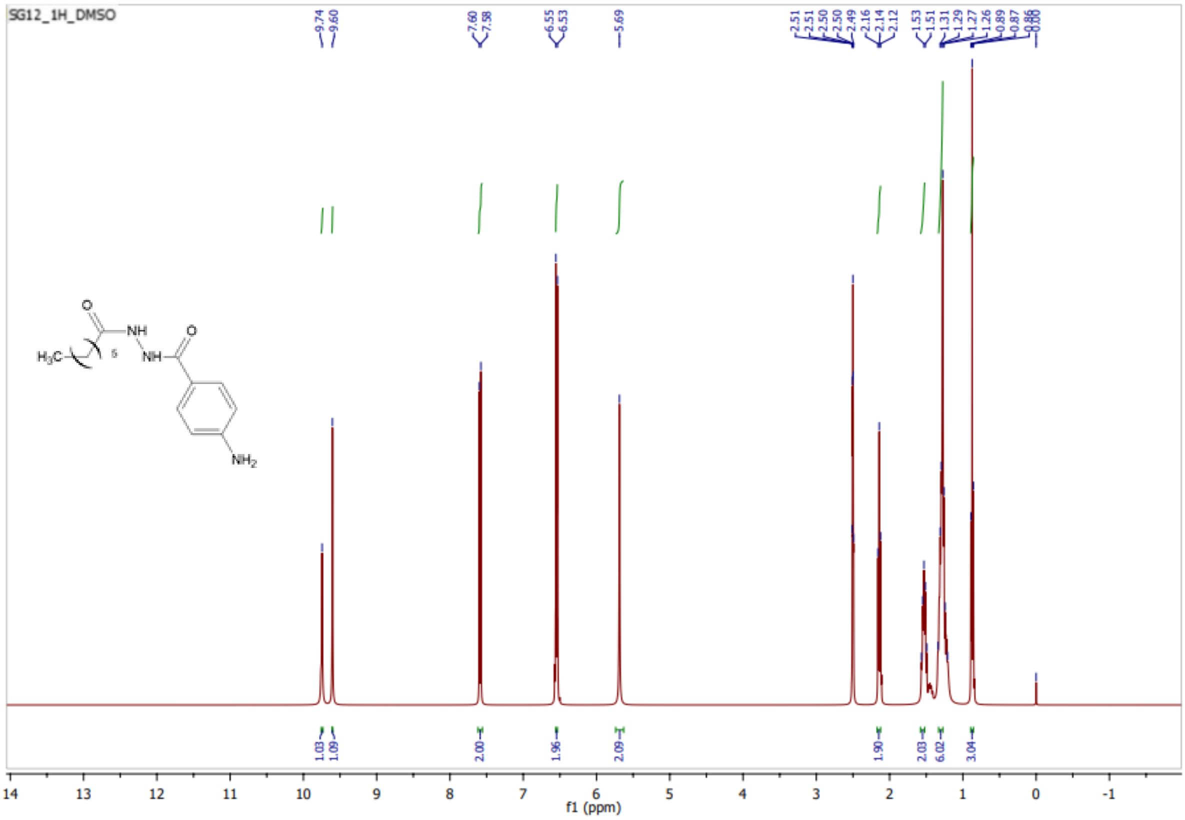

Figure S22. ^{1}H-NMR spectra (400 MHz, DMSO) of *4-Amino-N′-heptanoylbenzohydrazide* (**5e**)

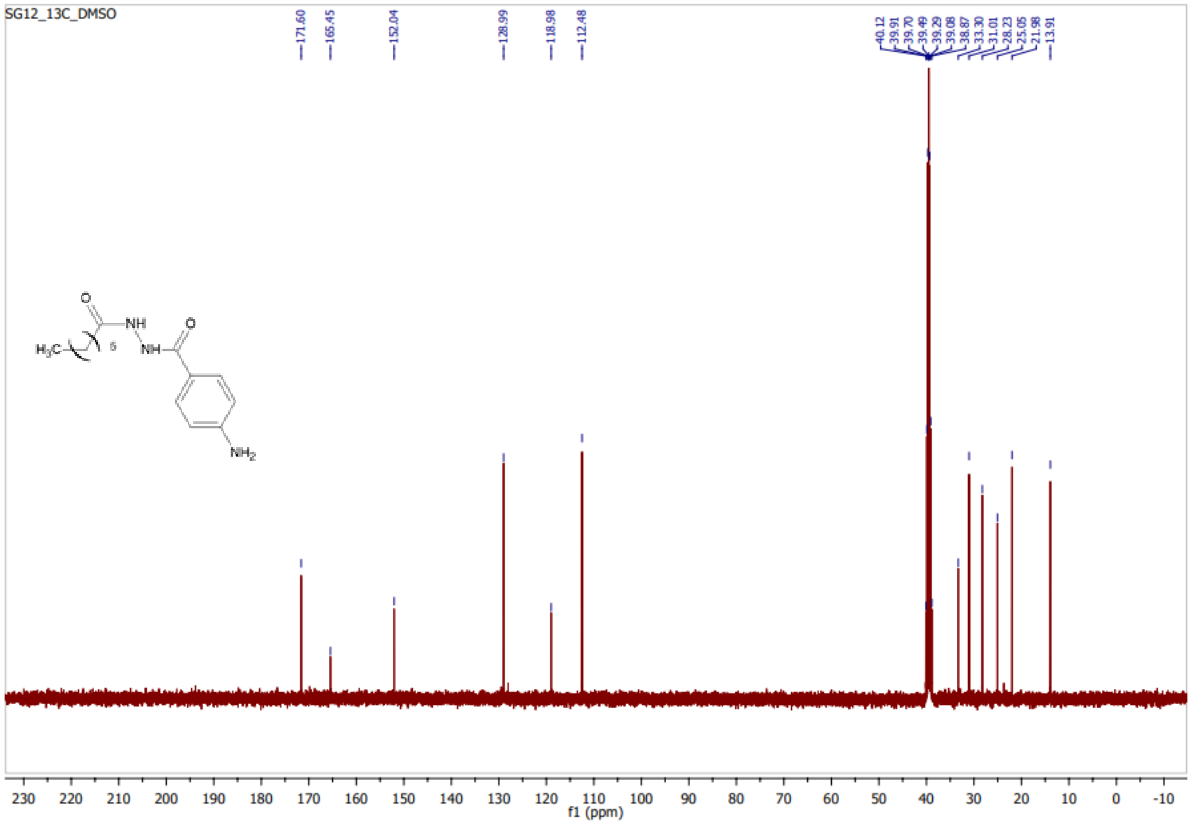

Figure S23. ^{13}C-NMR spectra (100 MHz, DMSO) of *4-Amino-N′-heptanoylbenzohydrazide* (**5e**)

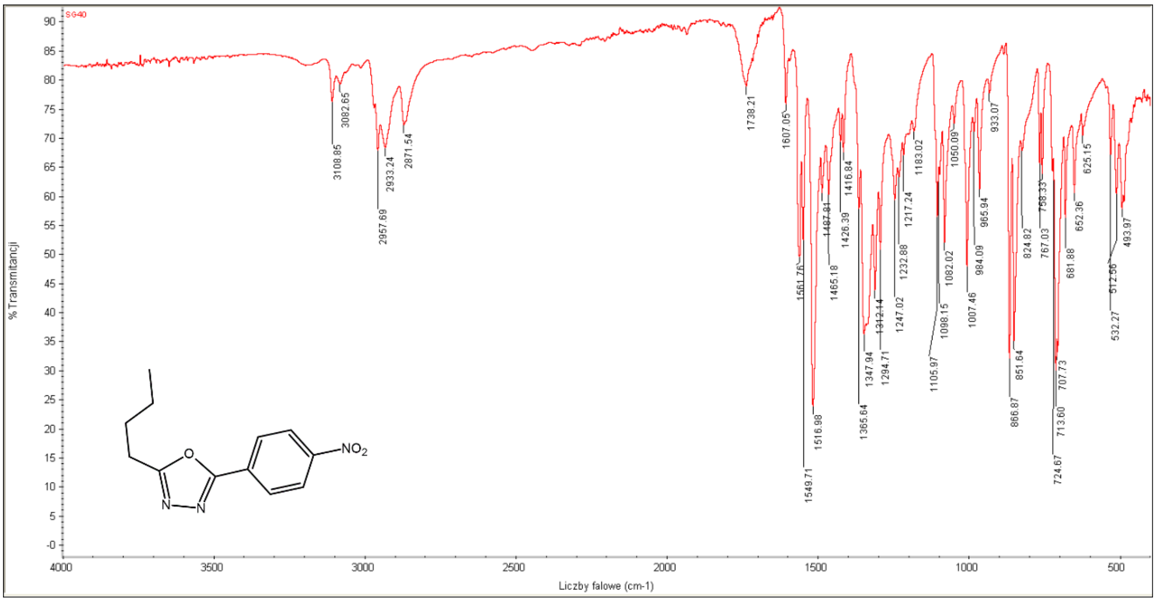

Figure S24. IR spectra of *2-Butyl-5-(4-nitrophenyl)-1,3,4-oxadiazole* (**6a**)

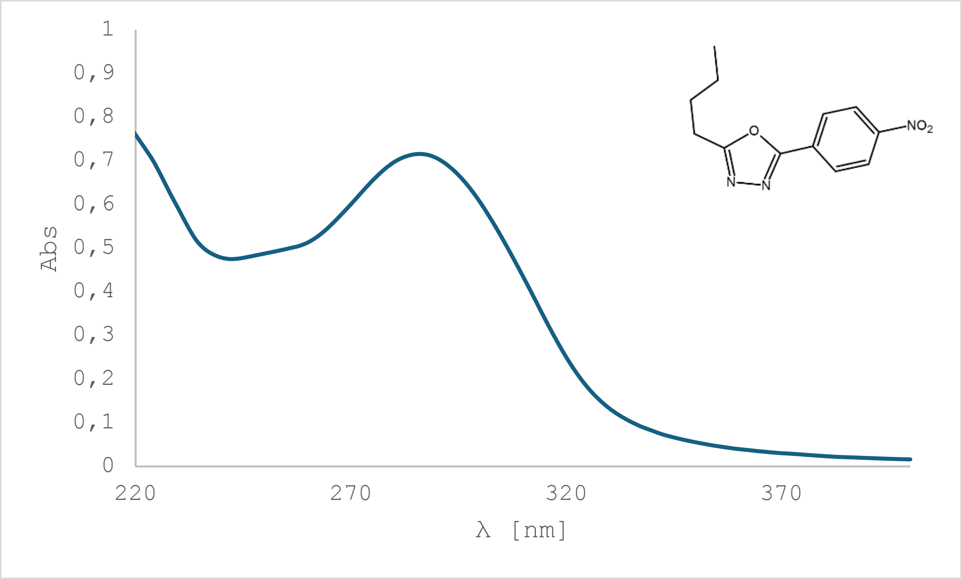

Figure S25. UV-Vis spectra (CH_3OH) of *2-Butyl-5-(4-nitrophenyl)-1,3,4-oxadiazole* (**6a**)

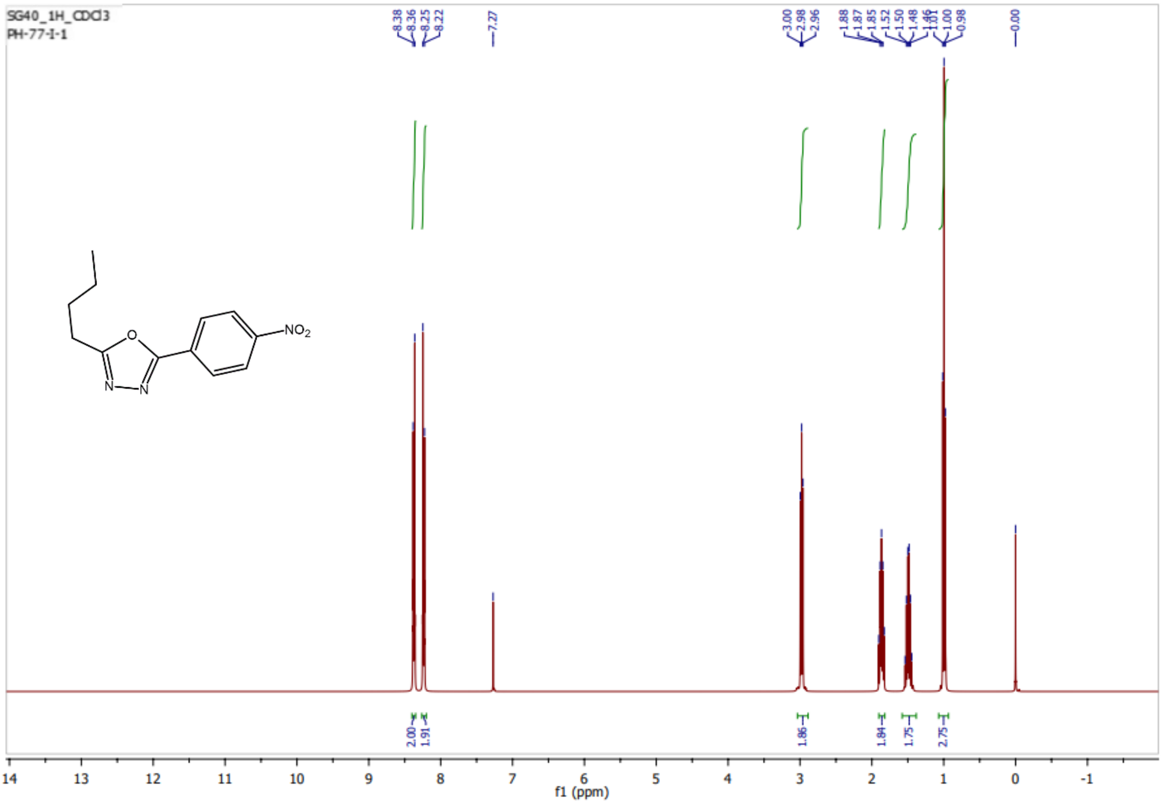

Figure S26. ^{1}H-NMR spectra (400 MHz, $CDCl_3$) of *2-Butyl-5-(4-nitrophenyl)-1,3,4-oxadiazole* (**6a**)

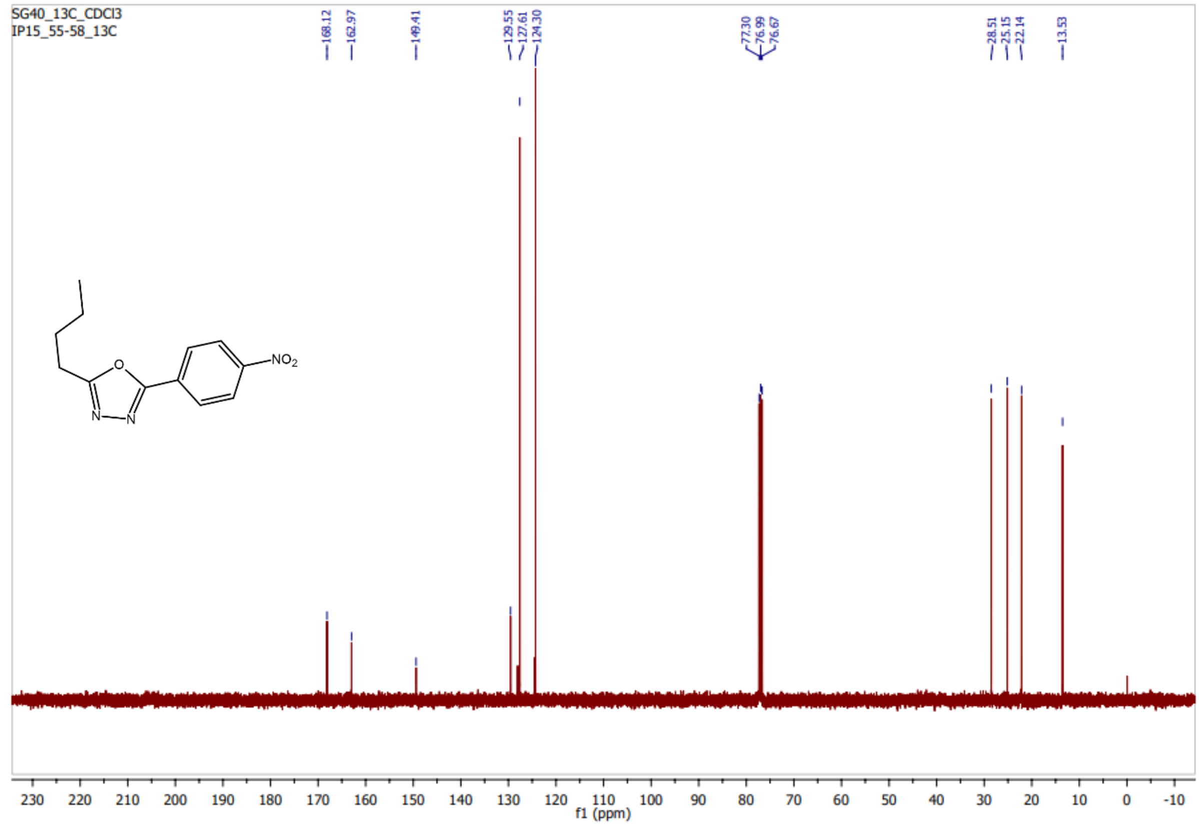

Figure S27. ¹³C-NMR spectra (100 MHz, $CDCl_3$) of *2-Butyl-5-(4-nitrophenyl)-1,3,4-oxadiazole* (**6a**)

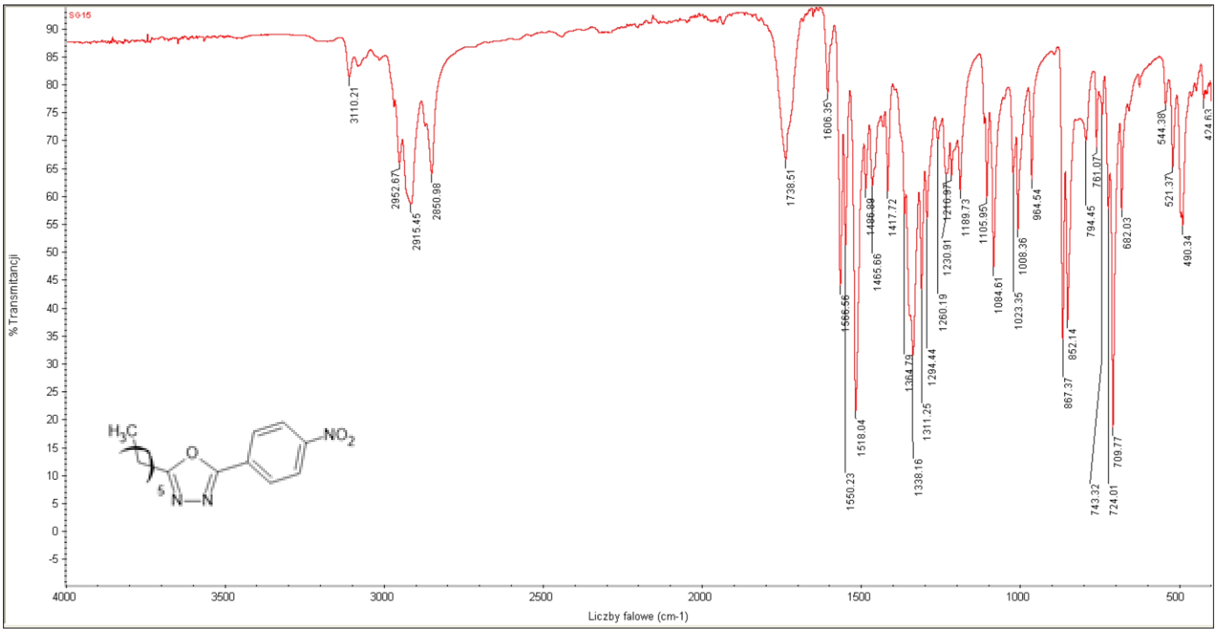

Figure S28. IR spectra of *2-Hexyl-5-(4-nitrophenyl)-1,3,4-oxadiazole* (**6b**)

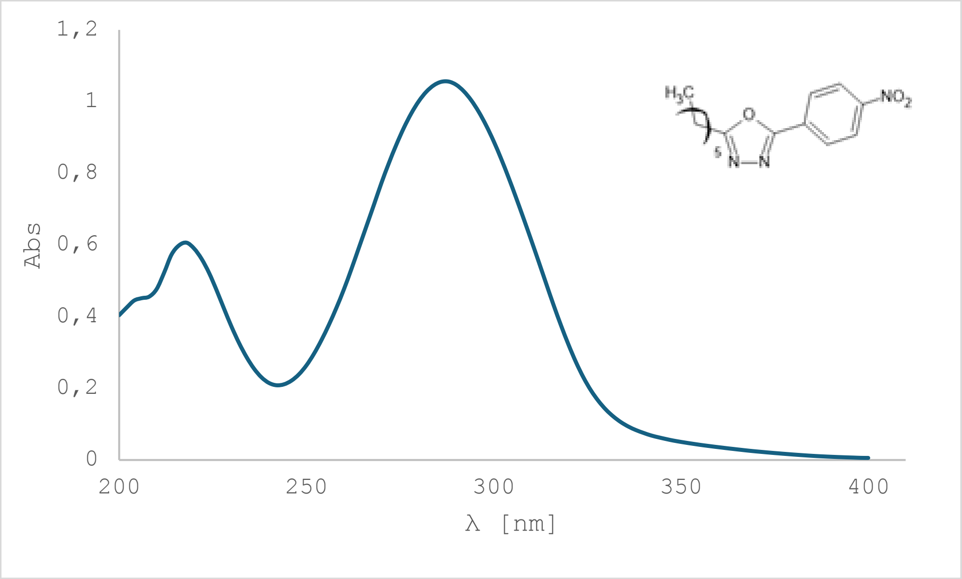

Figure S29. UV-Vis spectra (CH3OH) of *2-Hexyl-5-(4-nitrophenyl)-1,3,4-oxadiazole* (**6b**)

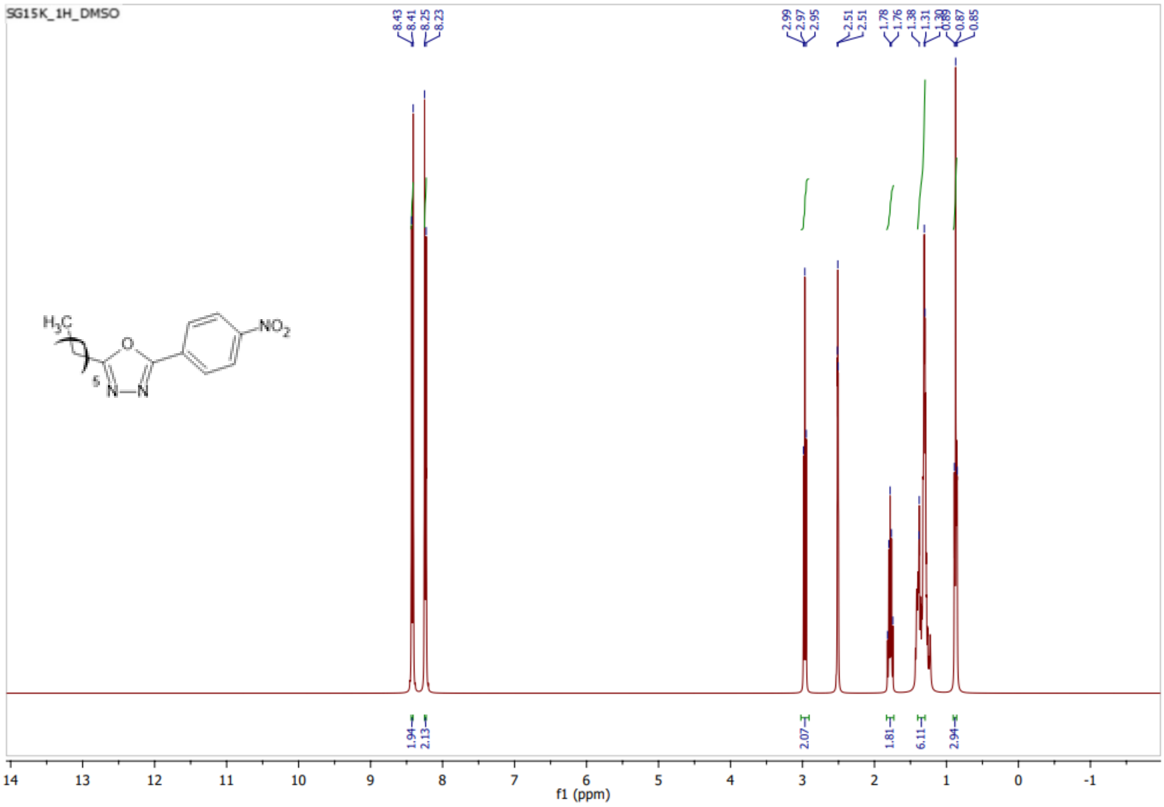

Figure S30. ^{1}H-NMR spectra (400 MHz, DMSO) of *2-Hexyl-5-(4-nitrophenyl)-1,3,4-oxadiazole* (**6b**)

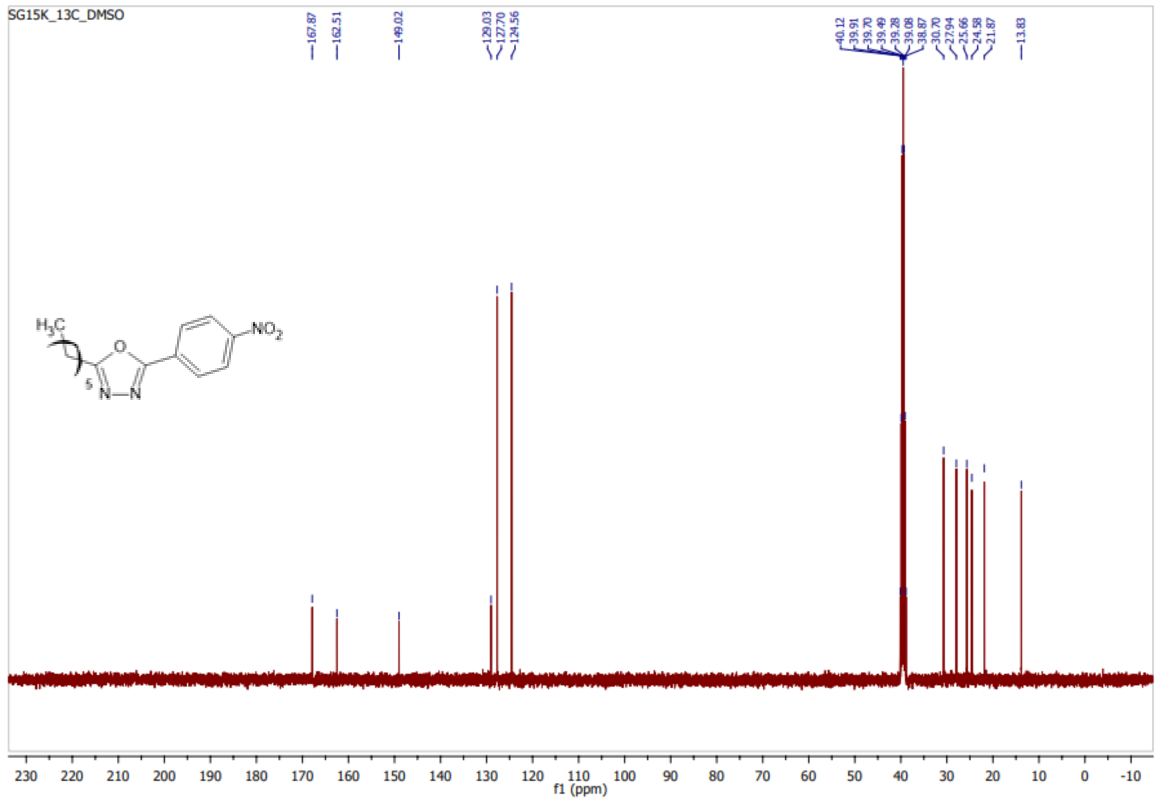

Figure S31. ^{13}C-NMR spectra (100 MHz, DMSO) of *2-Hexyl-5-(4-nitrophenyl)-1,3,4-oxadiazole* (**6b**)

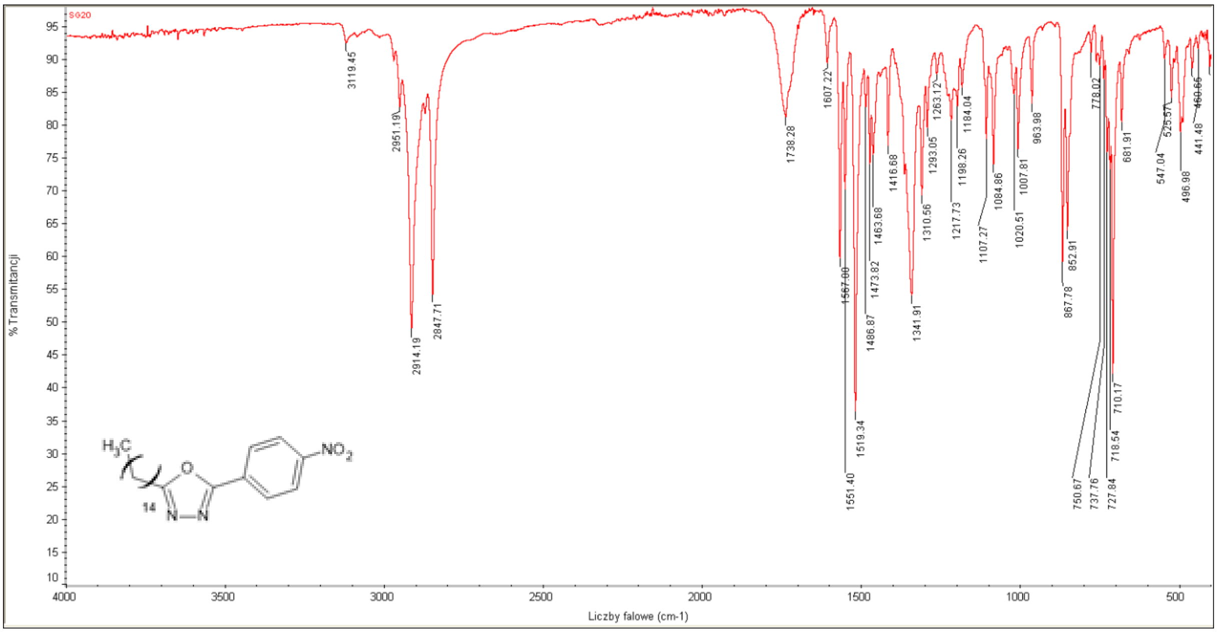

Figure S32. IR spectra of *2-Pentadecyl-5-(4-nitrophenyl)-1,3,4-oxadiazole* (**6c**)

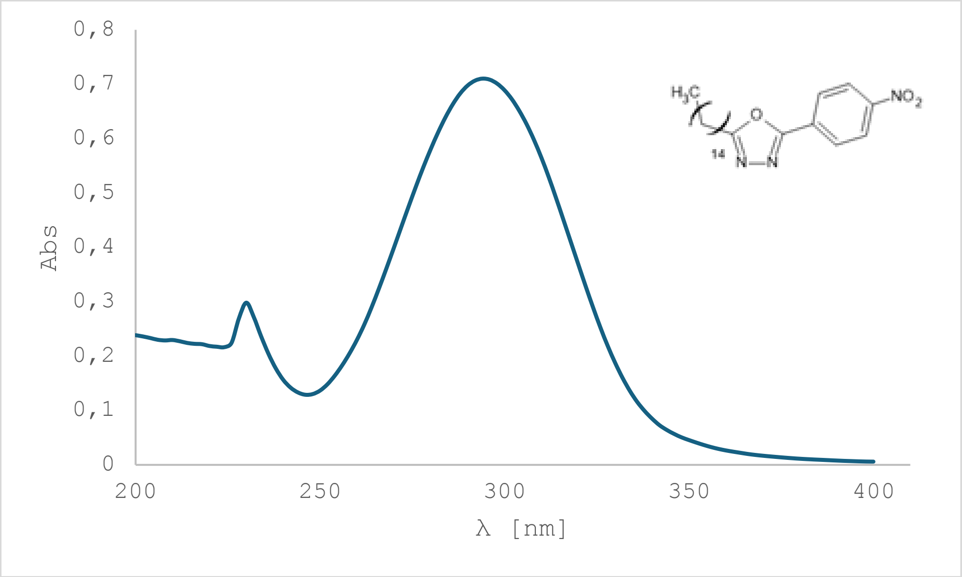

Figure S33. UV-Vis spectra (CH_2Cl_2) of *2-Pentadecyl-5-(4-nitrophenyl)-1,3,4-oxadiazole* (**6c**)

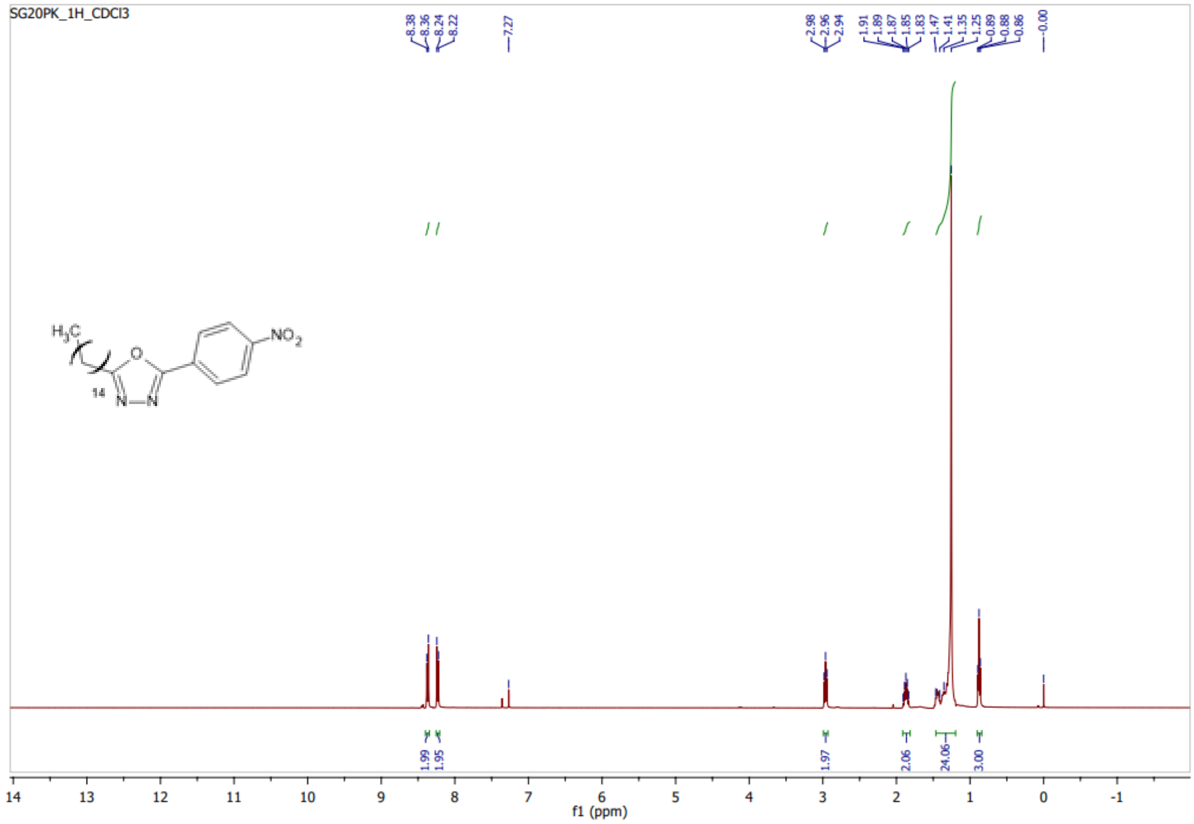

Figure S34. ^{1}H-NMR spectra (400 MHz, $CDCl_3$) of *2-Pentadecyl-5-(4-nitrophenyl)-1,3,4-oxadiazole* (**6c**)

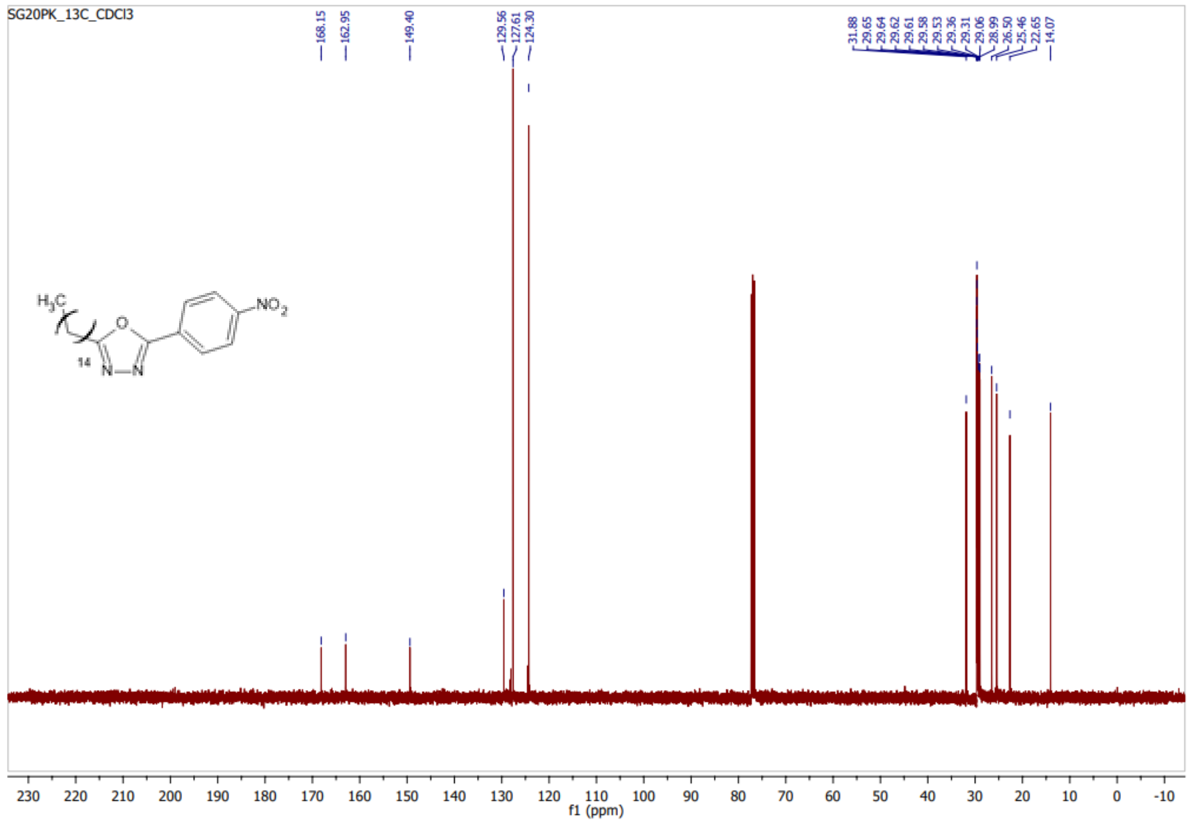

Figure S35. ^{13}C-NMR spectra (100 MHz, $CDCl_3$) of *2-Pentadecyl-5-(4-nitrophenyl)-1,3,4-oxadiazole* (**6c**)

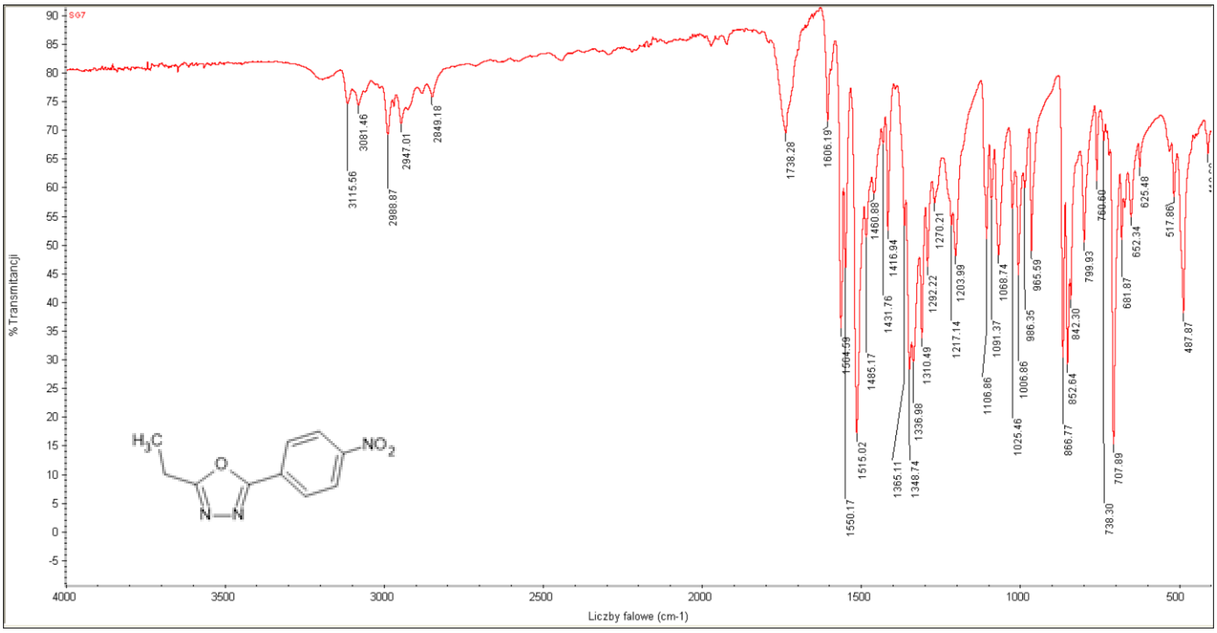

Figure S36. IR spectra of *2-Ethyl-5-(4-nitrophenyl)-1,3,4-oxadiazole* (**6d**)

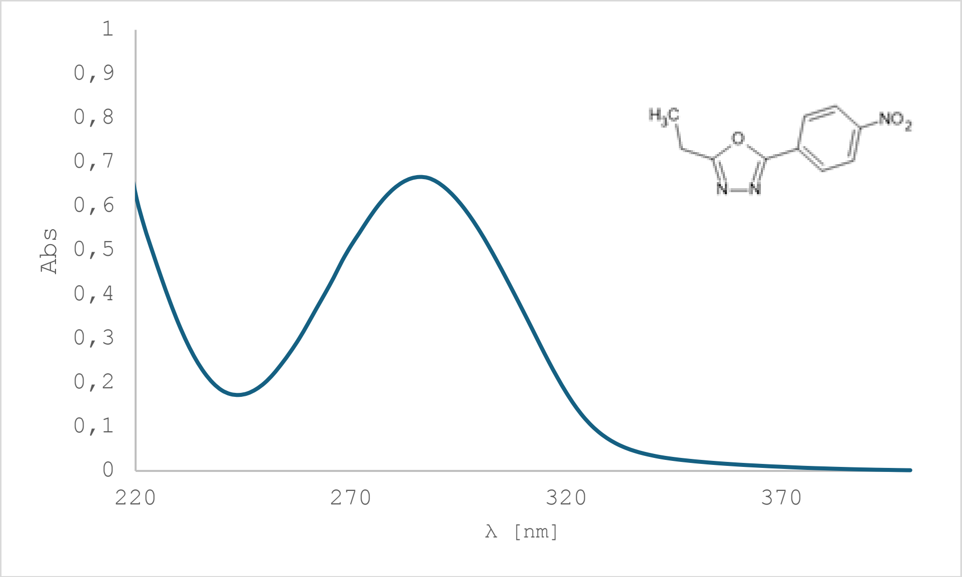

Figure S37. UV-Vis spectra (CH3OH) of *2-Ethyl-5-(4-nitrophenyl)-1,3,4-oxadiazole* (**6d**)

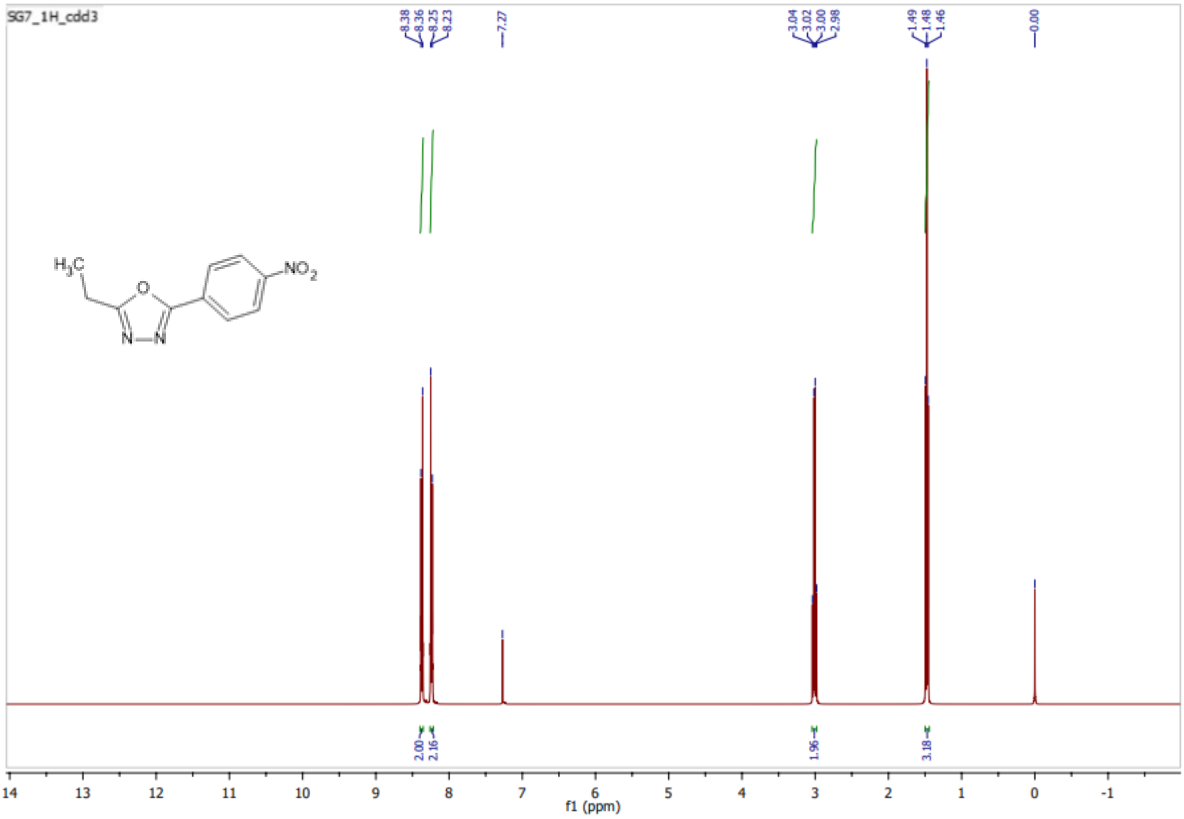

Figure S38. ^{1}H-NMR spectra (400 MHz, CDCl3) of *2-Ethyl-5-(4-nitrophenyl)-1,3,4-oxadiazole* (**6d**)

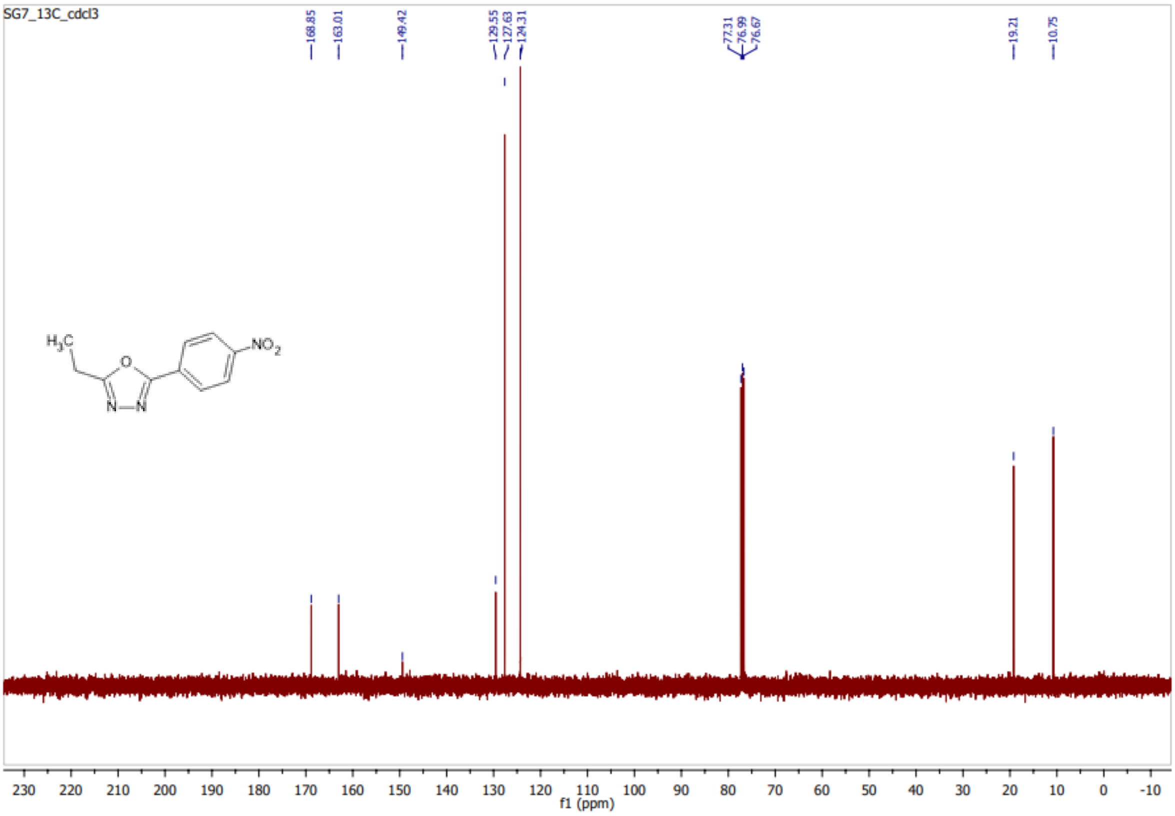

Figure S39. ^{13}C-NMR spectra (100 MHz, $CDCl_3$) of *2-Ethyl-5-(4-nitrophenyl)-1,3,4-oxadiazole* (**6d**)

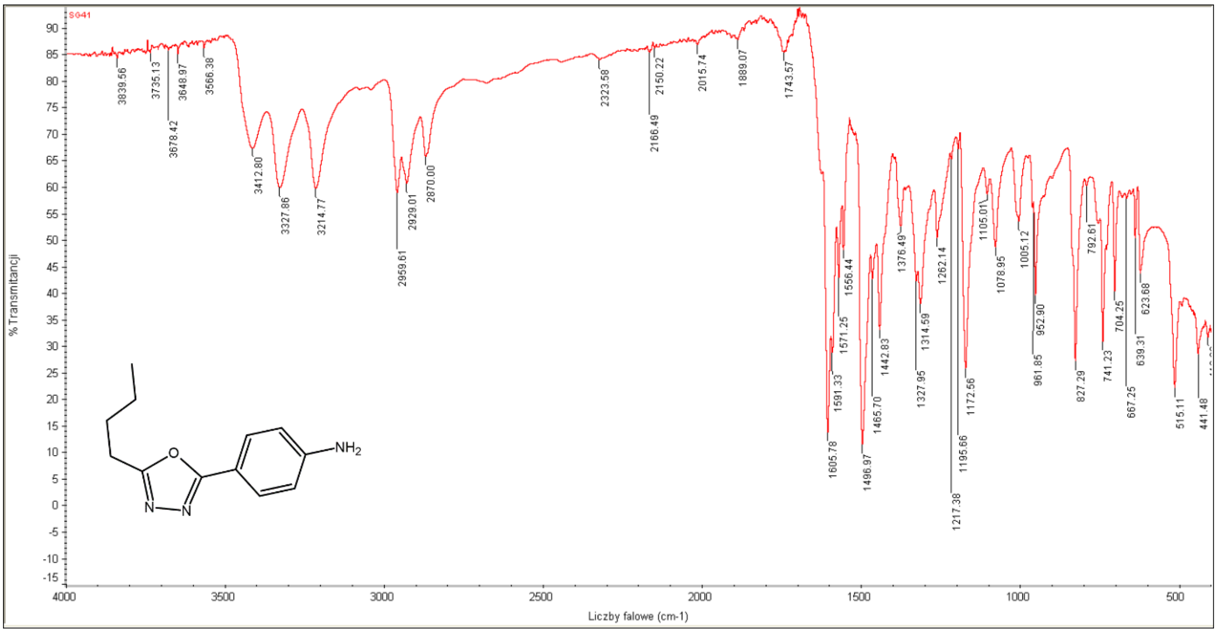

Figure S40. IR spectra of *4-(5-Butyl-1,3,4-oxadiazol-2-yl)aniline* (**7a**)

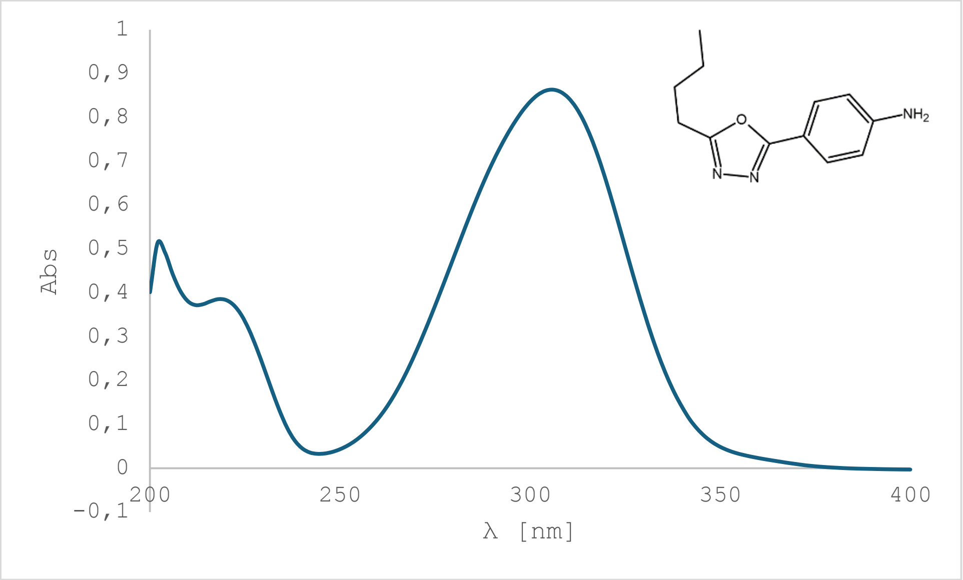

Figure S41. UV-Vis spectra (CH_3OH) of *4-(5-Butyl-1,3,4-oxadiazol-2-yl)aniline* (**7a**)

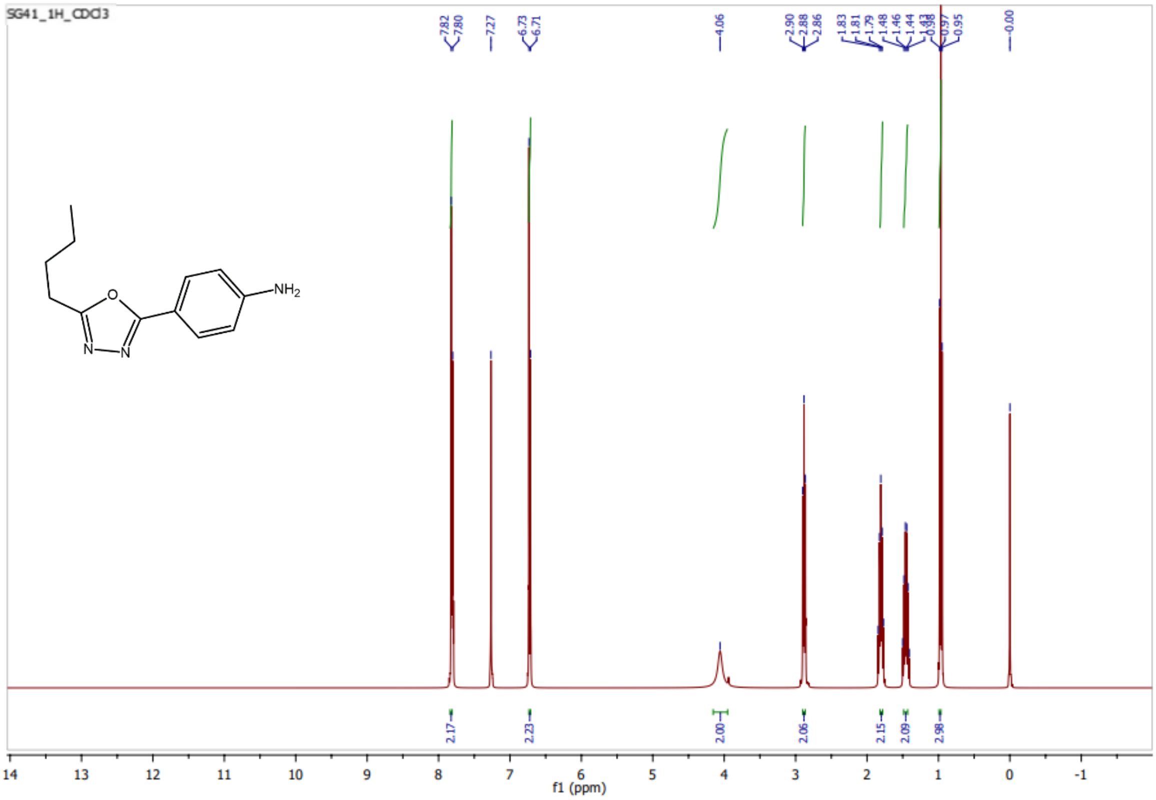

Figure S42. ^{1}H-NMR spectra (400 MHz, $CDCl_3$) of *4-(5-Butyl-1,3,4-oxadiazol-2-yl)aniline* (**7a**)

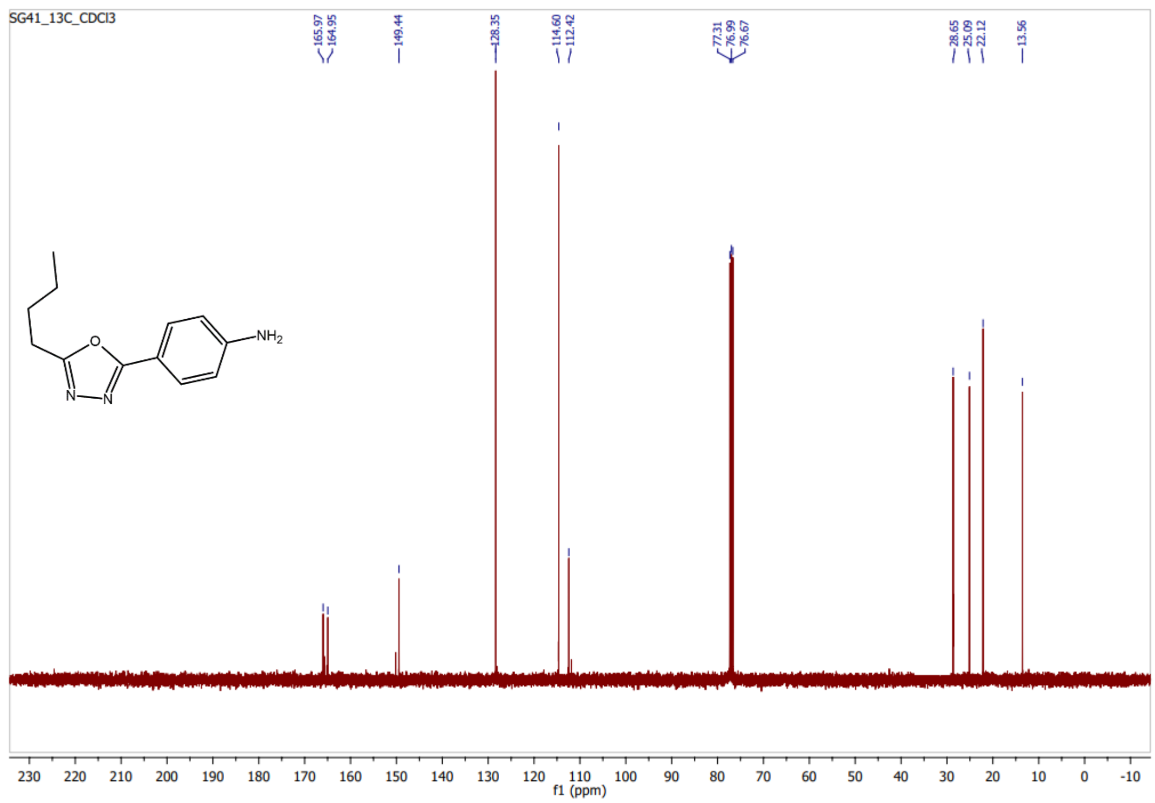

Figure S43. ^{13}C-NMR spectra (100 MHz, $CDCl_3$) of *4-(5-Butyl-1,3,4-oxadiazol-2-yl)aniline* (**7a**)

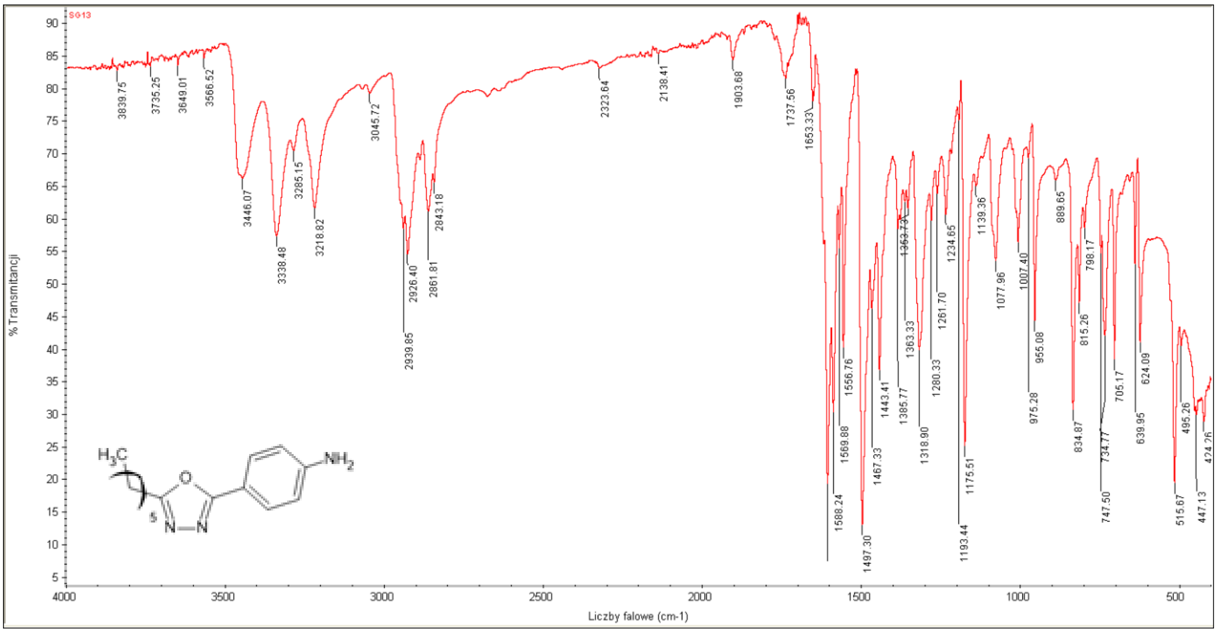

Figure S44. IR spectra of *4-(5-Hexyl-1,3,4-oxadiazol-2-yl)aniline* (**7b**)

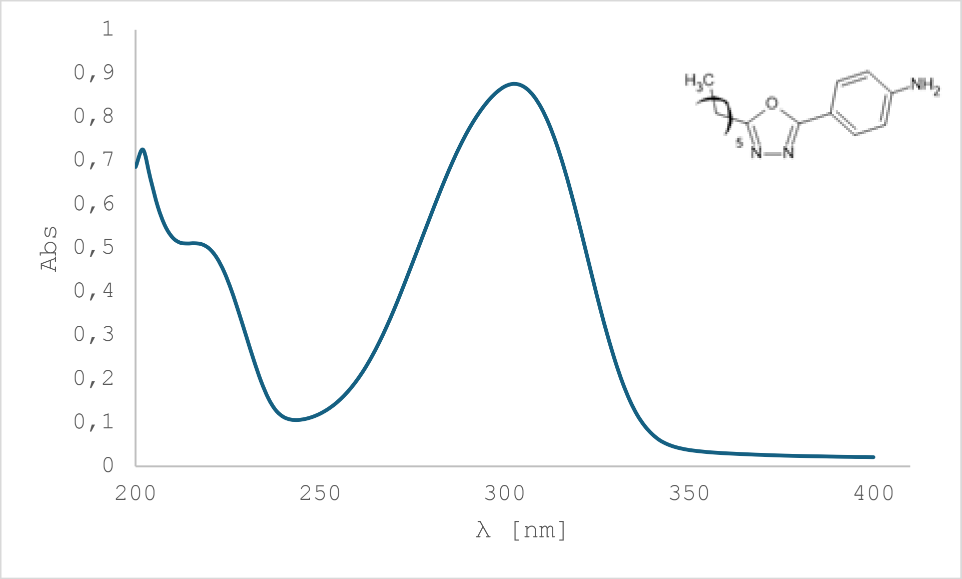

Figure S45. UV-Vis spectra (CH_3OH) of *4-(5-Hexyl-1,3,4-oxadiazol-2-yl)aniline* (**7b**)

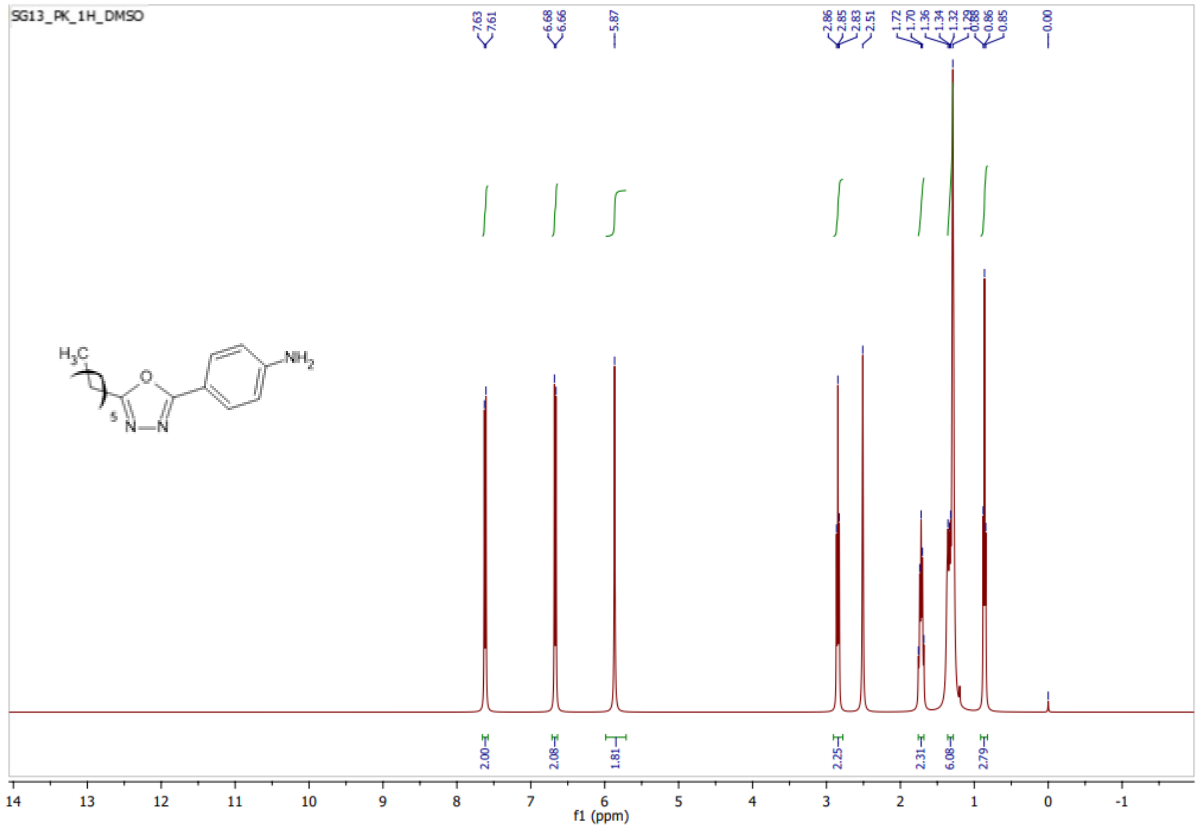

Figure S46. ^{1}H-NMR spectra (400 MHz, DMSO) of *4-(5-Hexyl-1,3,4-oxadiazol-2-yl)aniline* (**7b**)

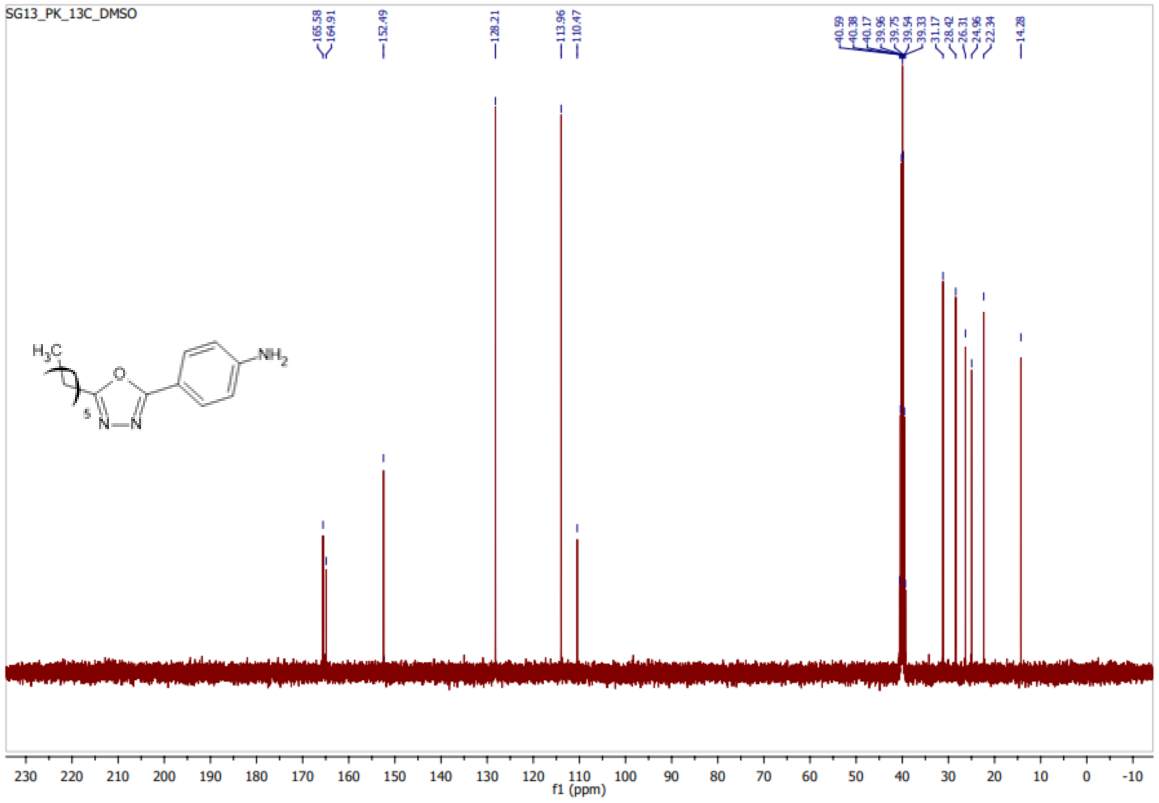

Figure S47. ^{13}C-NMR spectra (100 MHz, DMSO) of *4-(5-Hexyl-1,3,4-oxadiazol-2-yl)aniline* (**7b**)

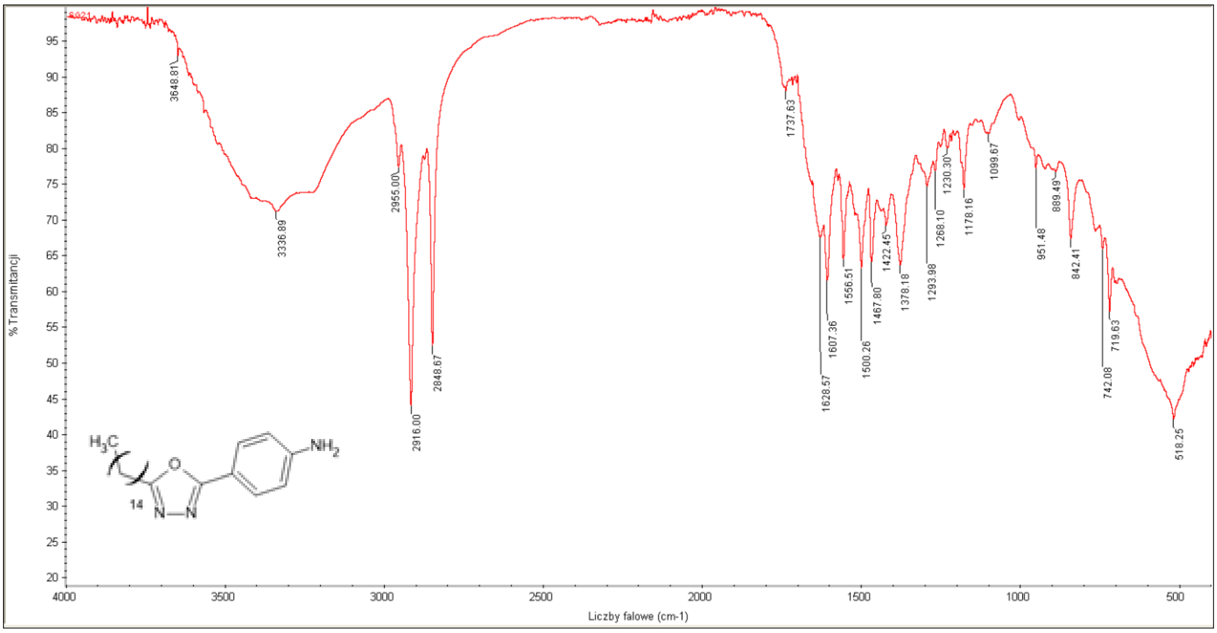

Figure S48. IR spectra of *4-(5-Pentadecyl-1,3,4-oxadiazol-2-yl)aniline* (**7c**)

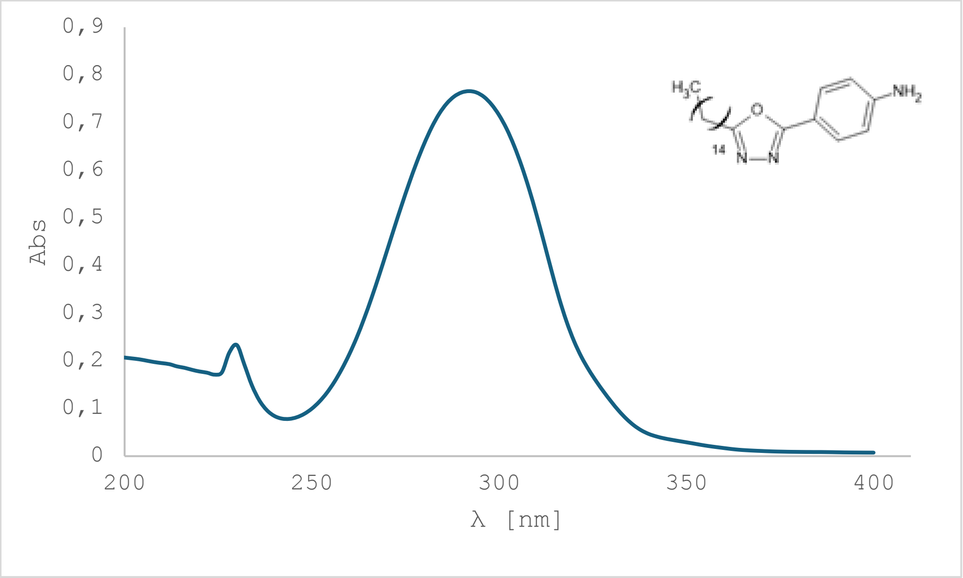

Figure S49. UV-Vis spectra (CH_2Cl_2) of *4-(5-Pentadecyl-1,3,4-oxadiazol-2-yl)aniline* (**7c**)

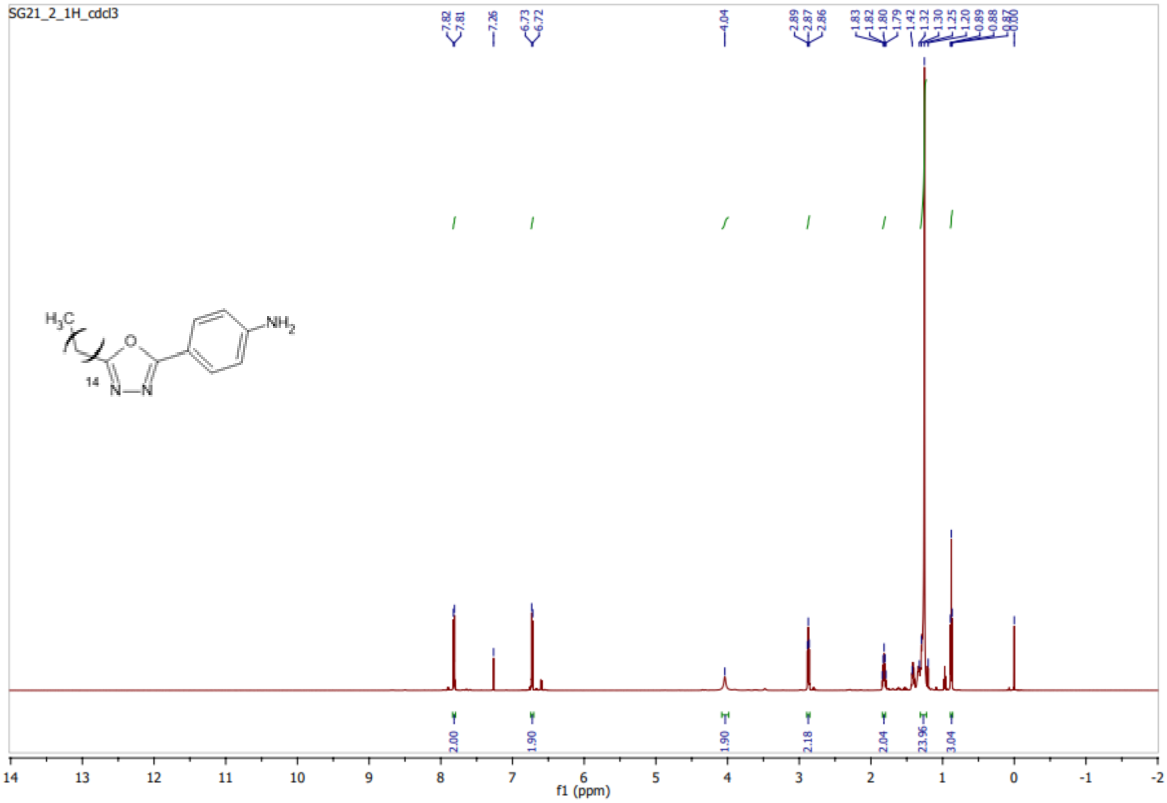

Figure S50. ^{1}H-NMR spectra (600 MHz, $CDCl_3$) of *4-(5-Pentadecyl-1,3,4-oxadiazol-2-yl)aniline* (**7c**)

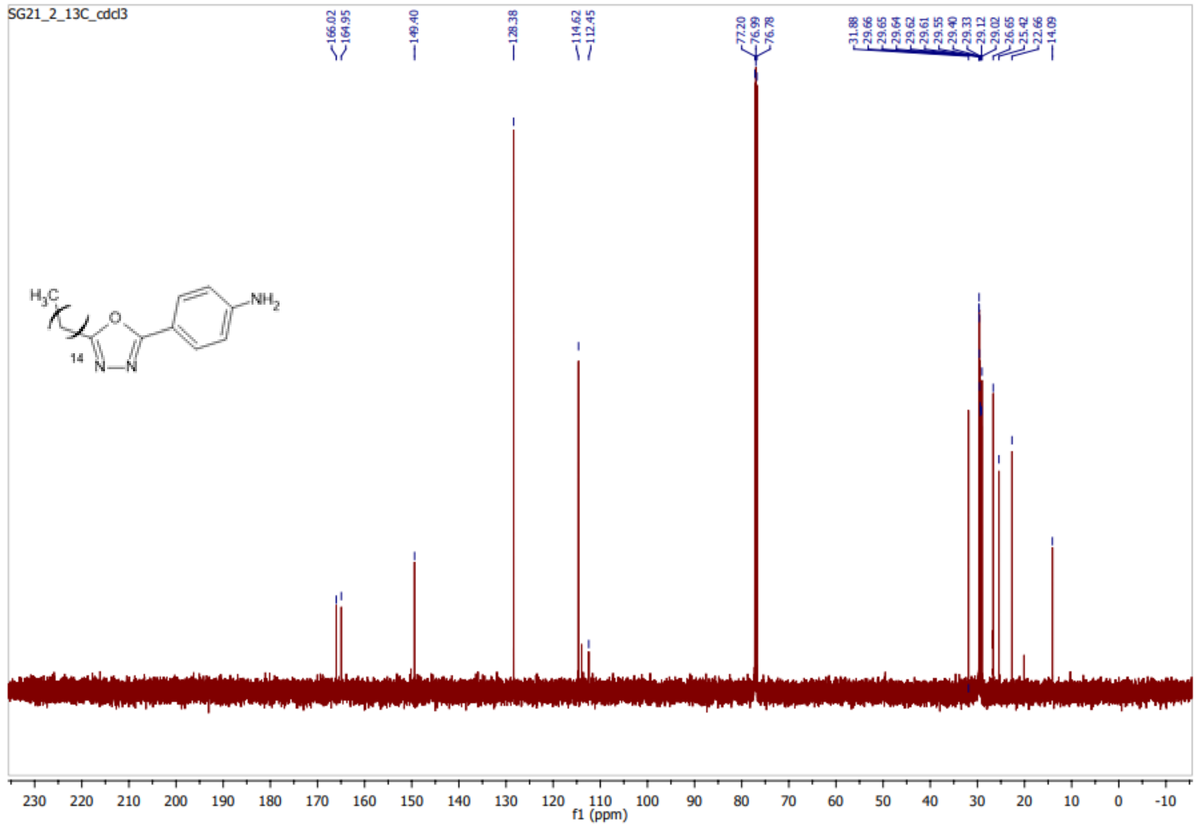

Figure S51. ^{13}C-NMR spectra (151 MHz, $CDCl_3$) of *4-(5-Pentadecyl-1,3,4-oxadiazol-2-yl)aniline* (**7c**)

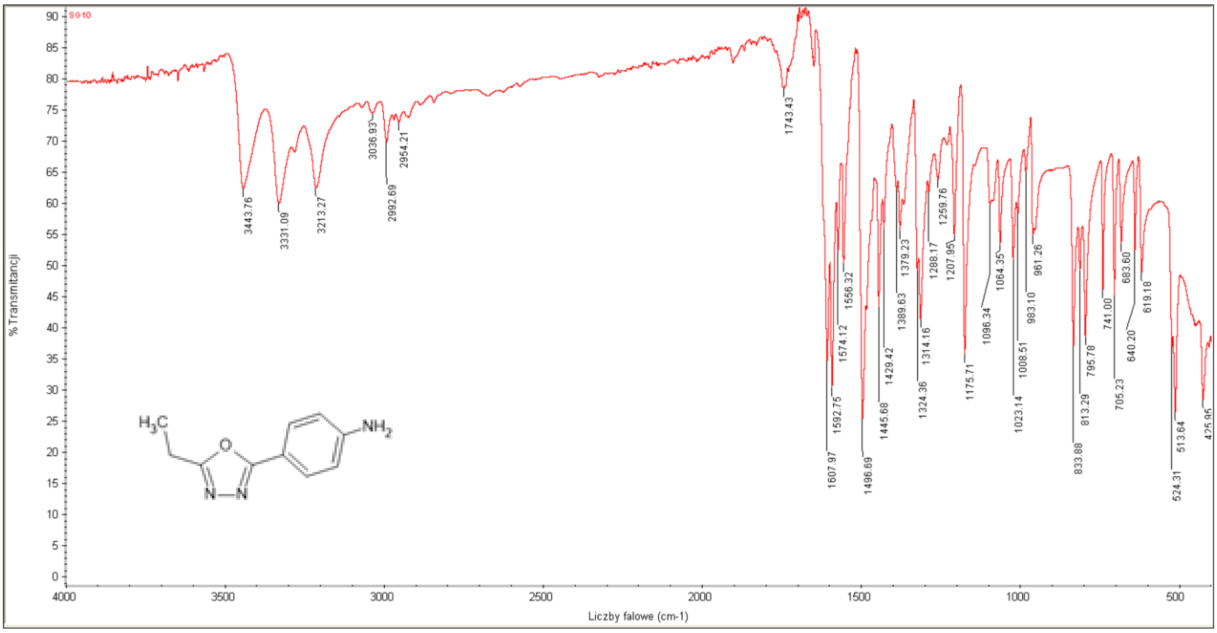

Figure S52. IR spectra of *4-(5-Ethyl-1,3,4-oxadiazol-2-yl)aniline* **(7d)**

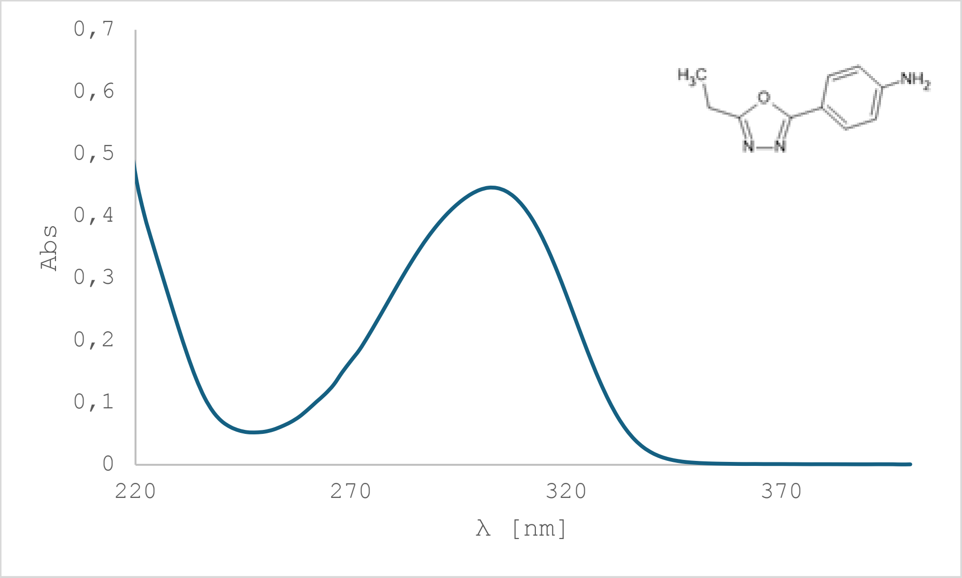

Figure S53. UV-Vis spectra (CH3OH) of *4-(5-Ethyl-1,3,4-oxadiazol-2-yl)aniline* (**7d**)

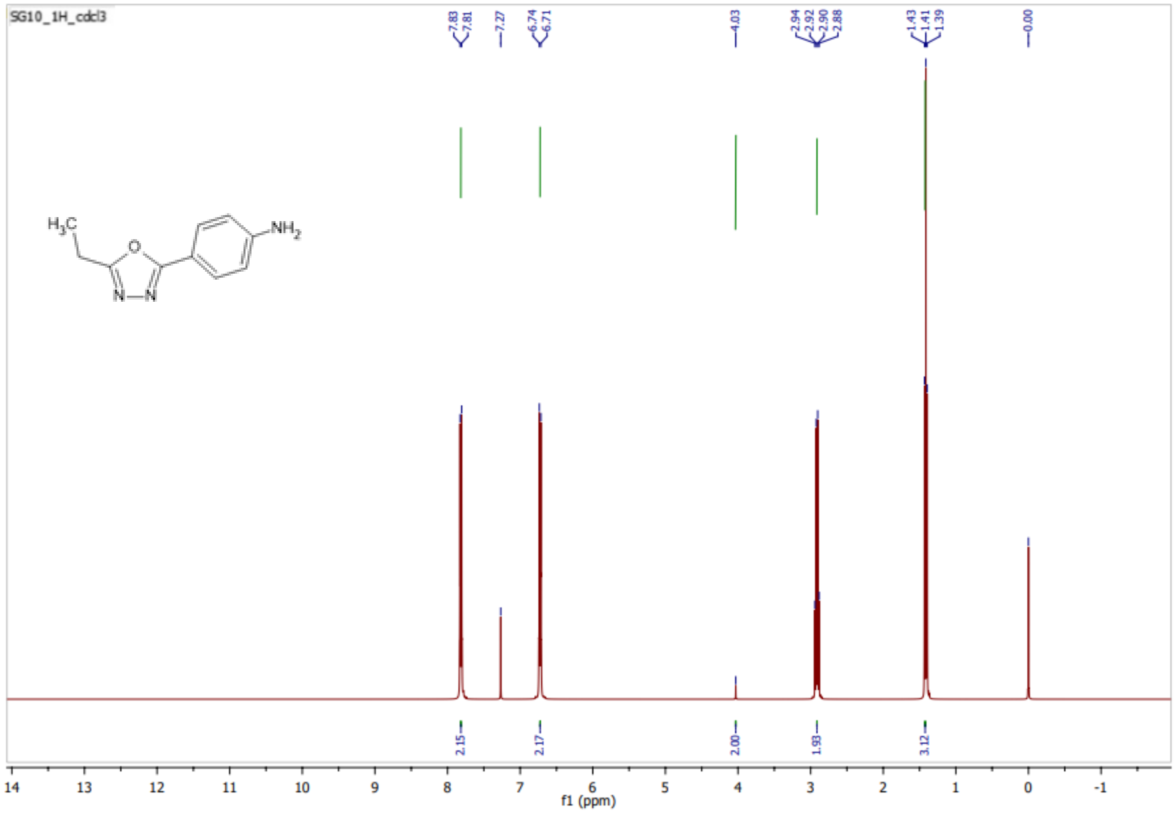

Figure S54. ^{1}H-NMR spectra (400 MHz, CDCl3) of *4-(5-Ethyl-1,3,4-oxadiazol-2-yl)aniline* (**7d**)

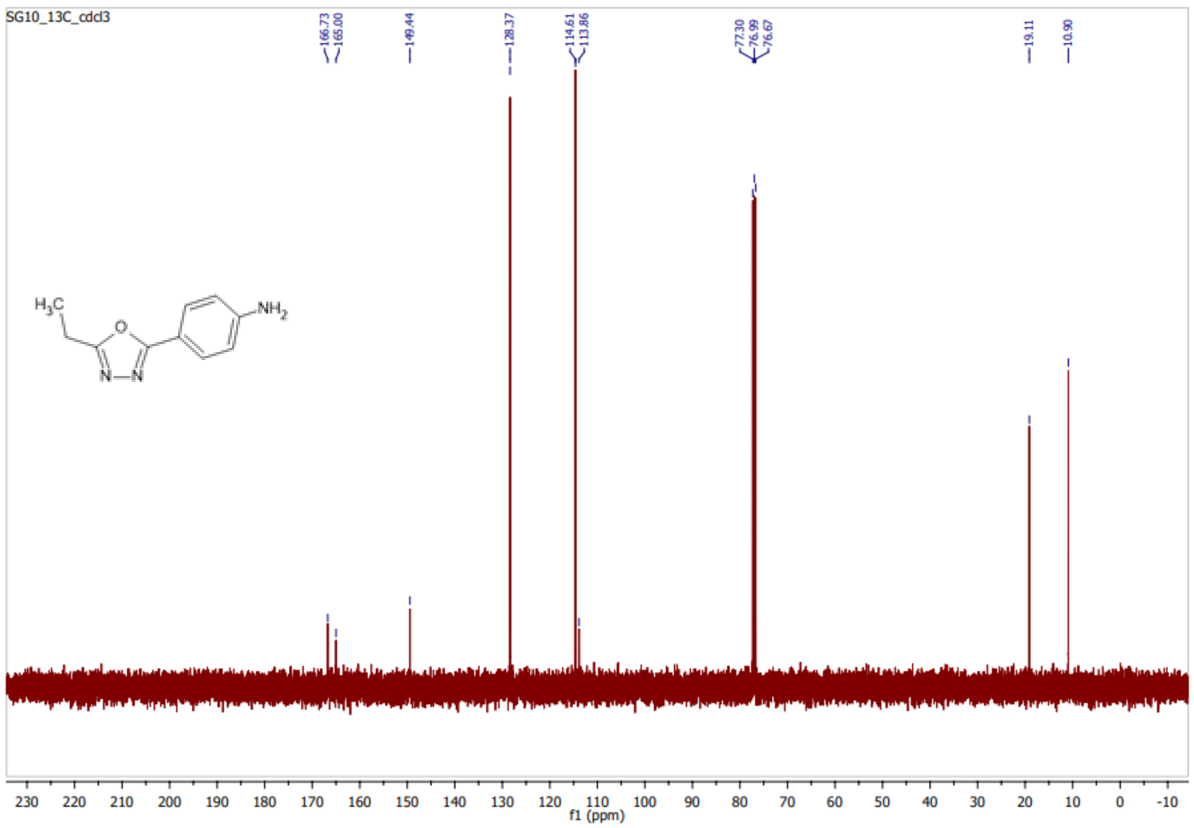

Figure S55. ^{13}C-NMR spectra (100 MHz, $CDCl_3$) of *4-(5-Ethyl-1,3,4-oxadiazol-2-yl)aniline* (**7d**)

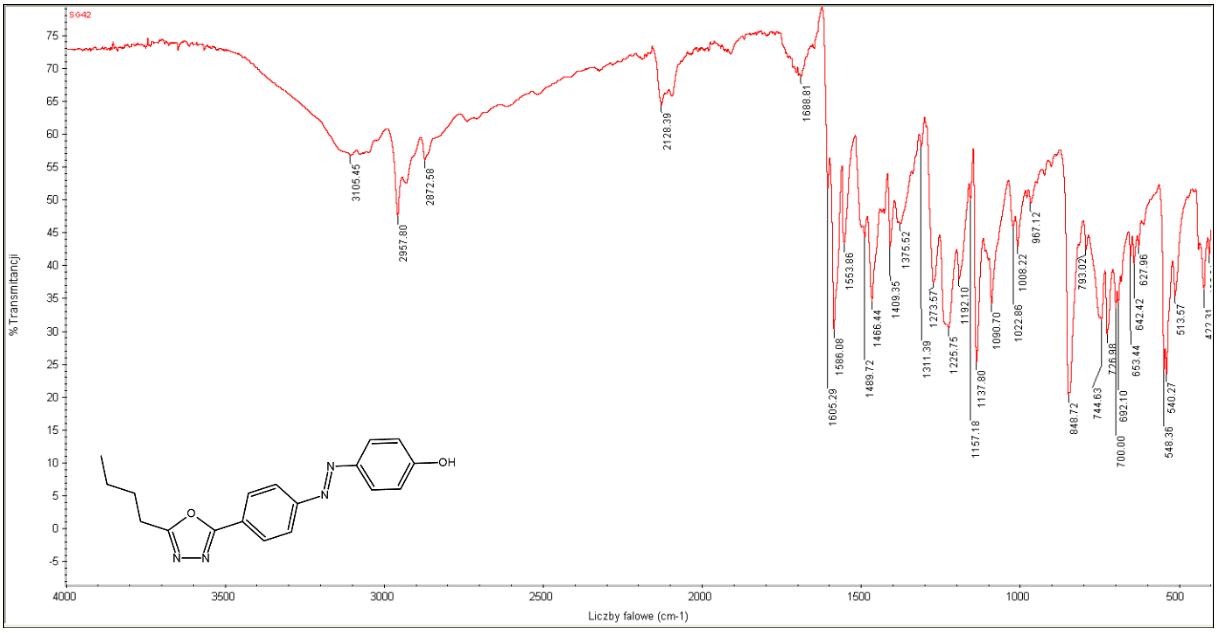

Figure S56. IR spectra of *4-{[4-(5-Butyl-1,3,4-oxadiazol-2-yl)phenyl]diazenyl}phenol* **(8a)**

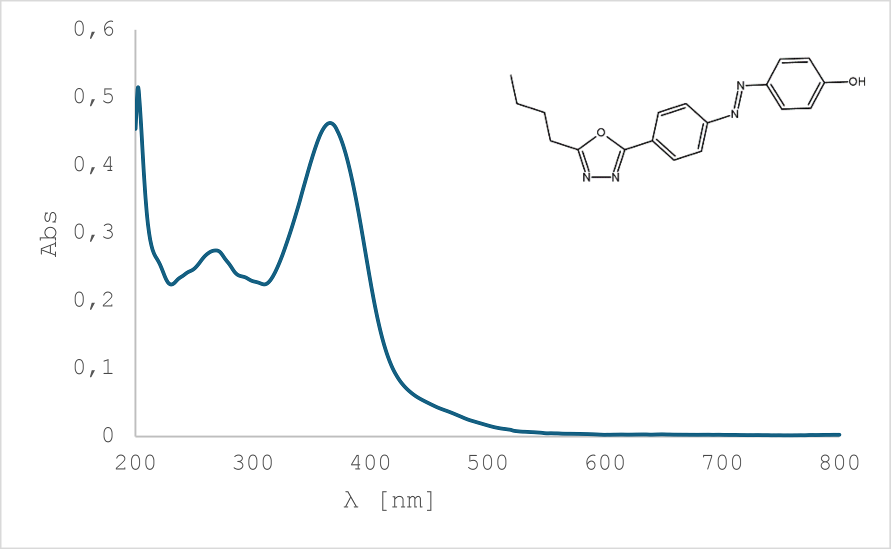

Figure S57. UV-Vis spectra (CH_3OH) of *4-{[4-(5-Butyl-1,3,4-oxadiazol-2-yl)phenyl]diazenyl}phenol* **(8a)**

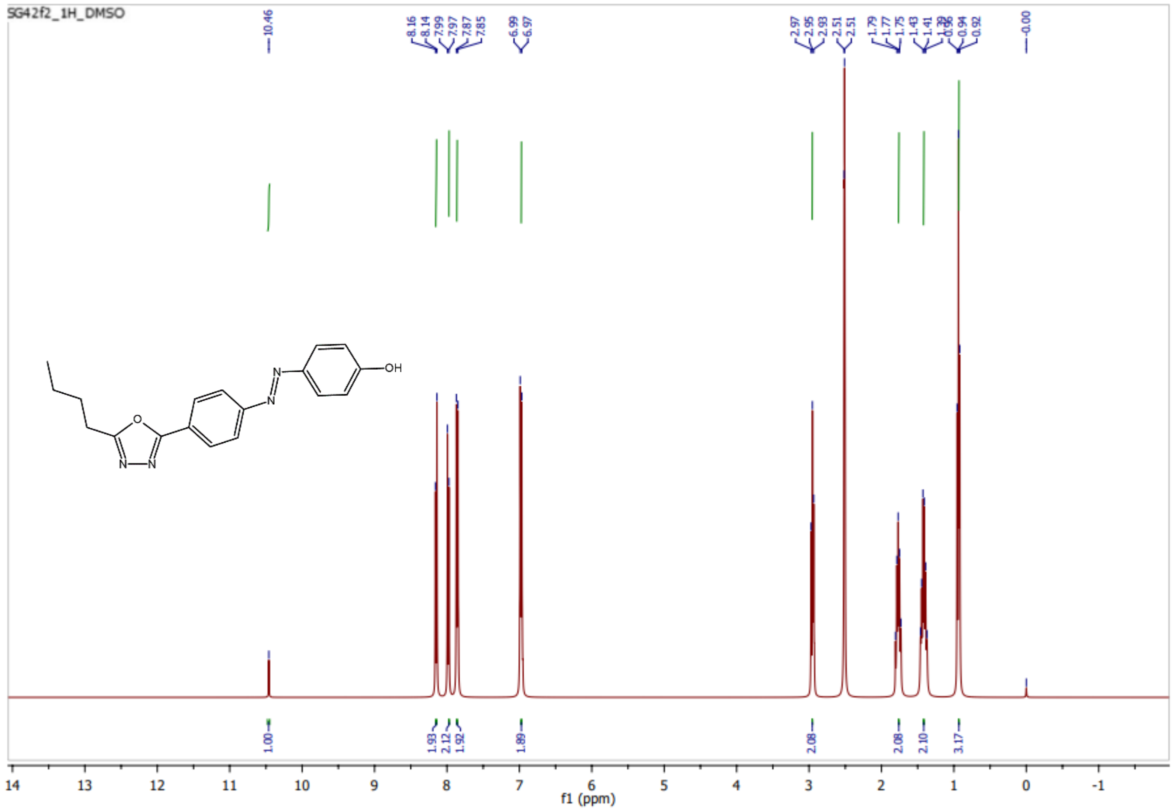

Figure S58. ¹H-NMR spectra (400 MHz, DMSO) of *4-{[4-(5-Butyl-1,3,4-oxadiazol-2-yl)phenyl]diazenyl}phenol* **(8a)**

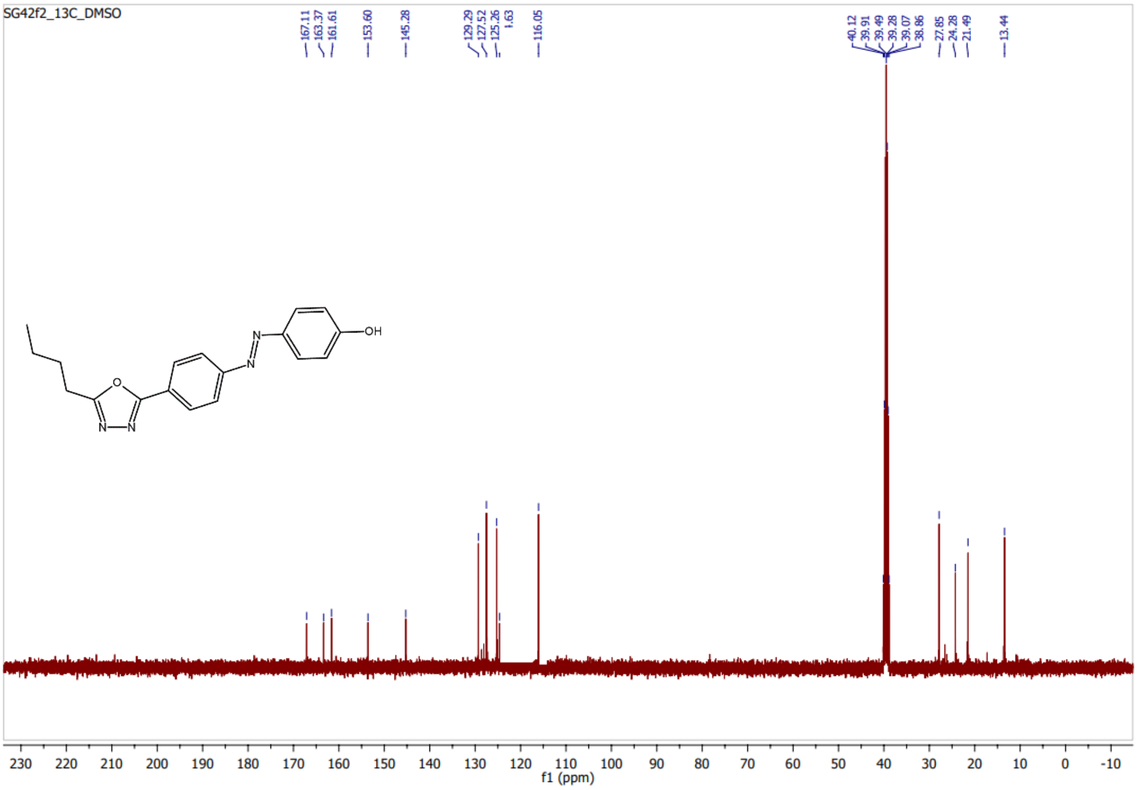

Figure S59. ^{13}C-NMR spectra (100 MHz, DMSO) of *4-{[4-(5-Butyl-1,3,4-oxadiazol-2-yl)phenyl]diazenyl}phenol* **(8a)**

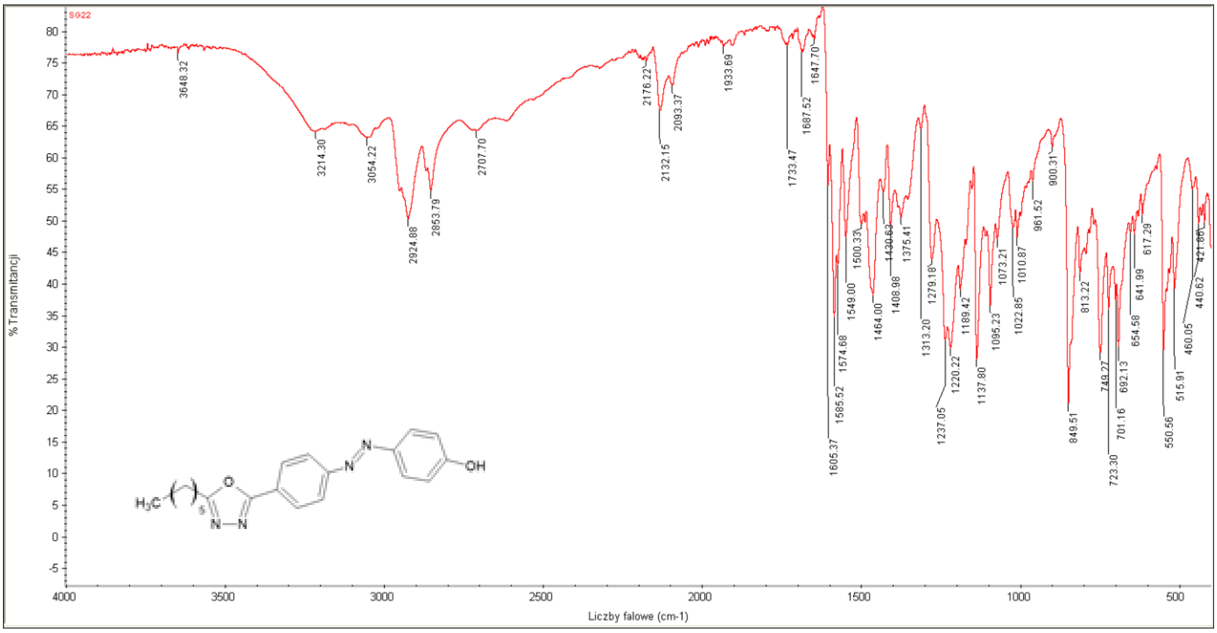

Figure S60. IR spectra of *4-{[4-(5-Hexyl-1,3,4-oxadiazol-2-yl)phenyl]diazenyl}phenol* **(8b)**

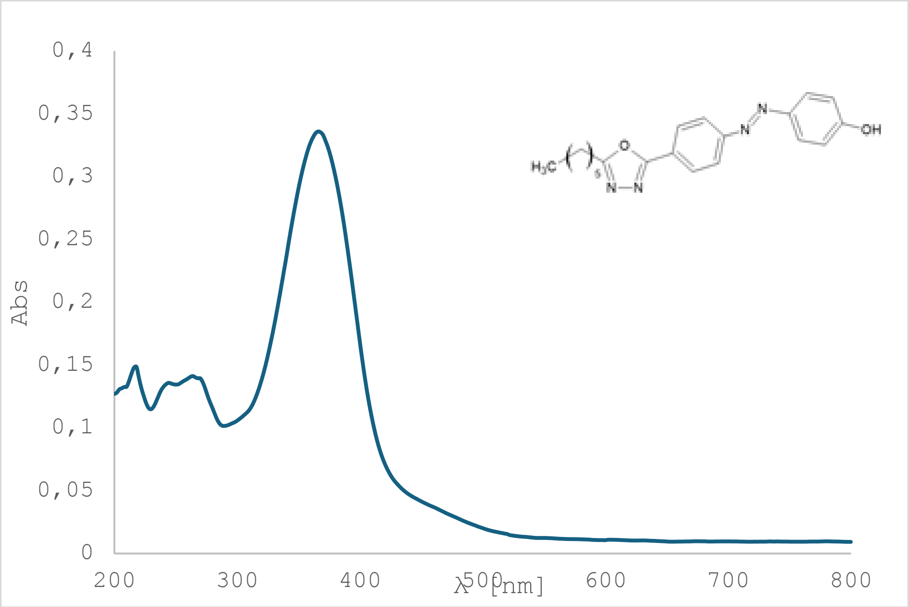

Figure S61. UV-Vis spectra (CH_3OH) of *4-{[4-(5-Hexyl-1,3,4-oxadiazol-2-yl)phenyl]diazenyl}phenol* **(8b)**

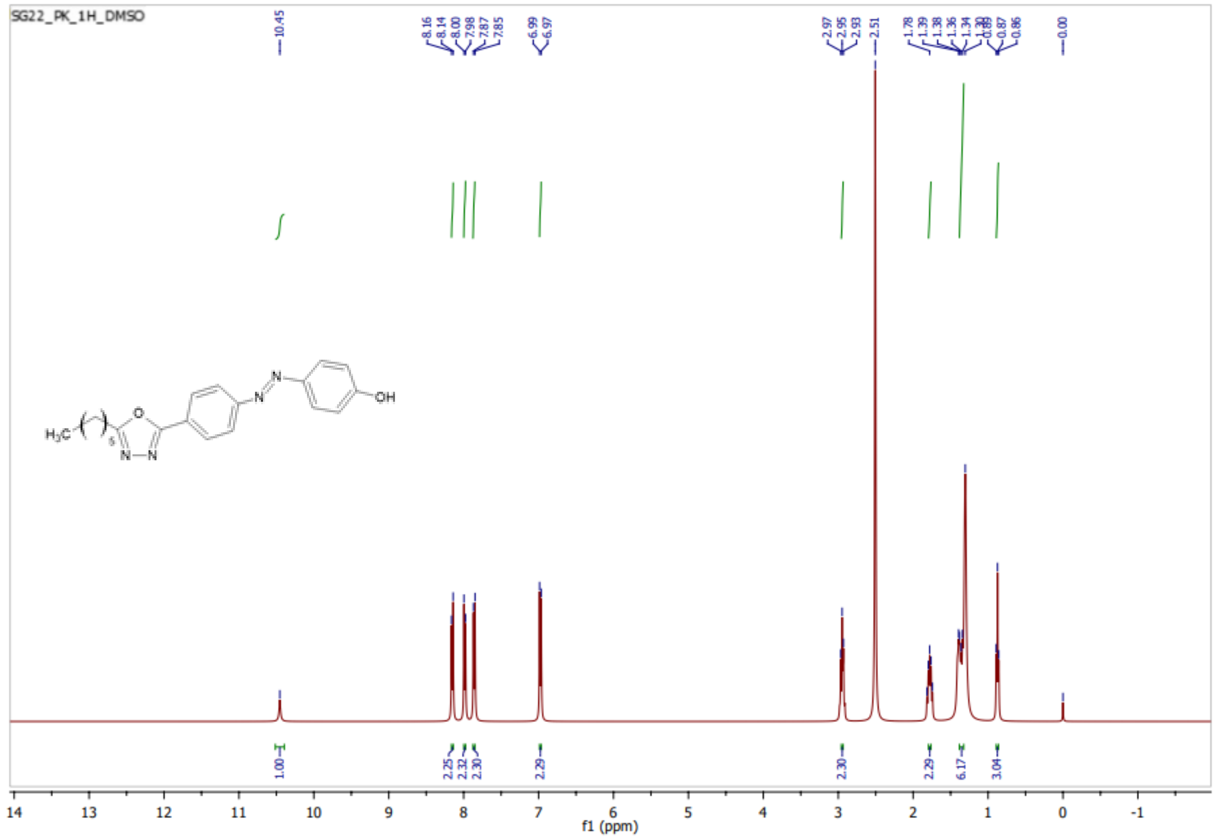

Figure S62. ¹H-NMR spectra (400 MHz, DMSO) of *4-{[4-(5-Heksyl-1,3,4-oxadiazol-2-yl)phenyl]diazenyl}phenol* **(8b)**

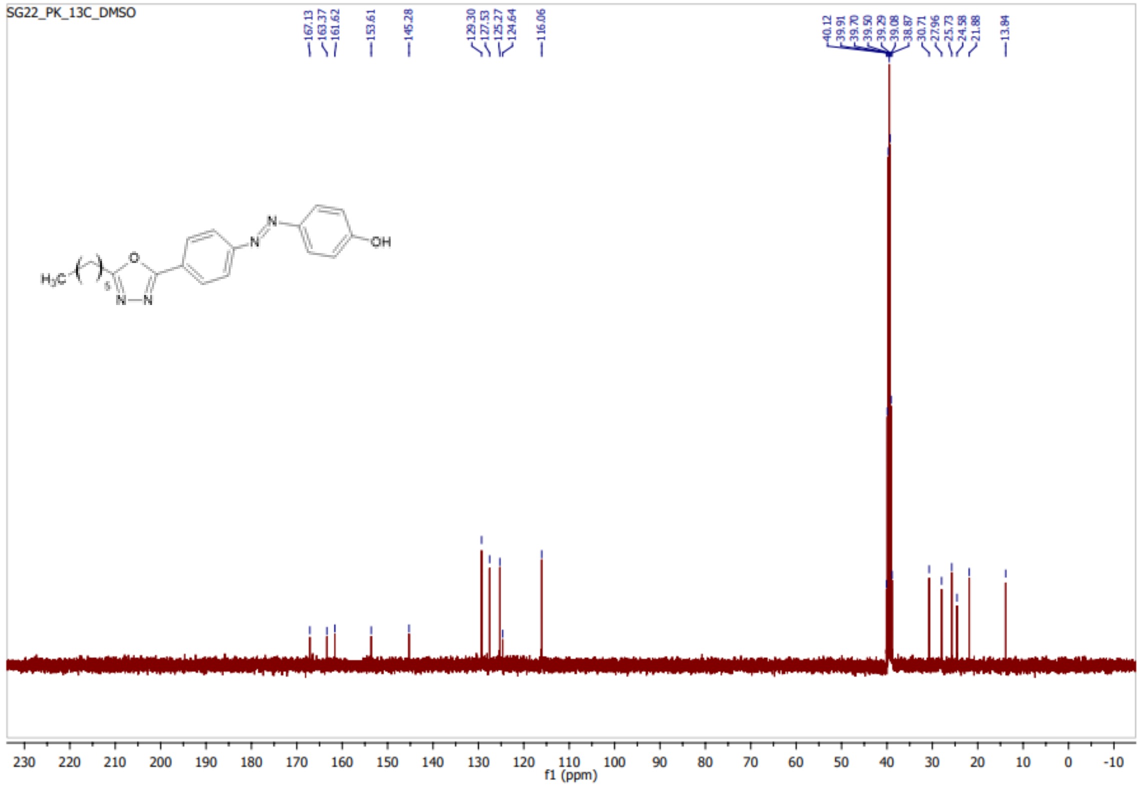

Figure S63. ^{13}C-NMR spectra (100 MHz, DMSO) of *4-{[4-(5-Heksyl-1,3,4-oxadiazol-2-yl)phenyl]diazenyl}phenol* **(8b)**

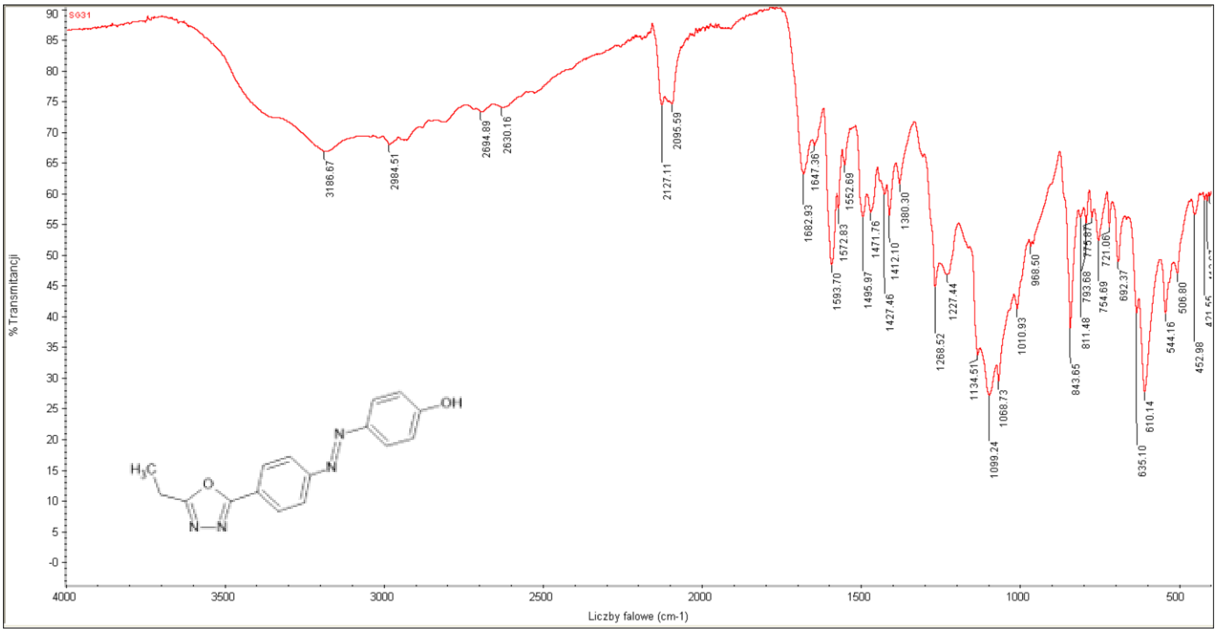

Figure S64. IR spectra of *4-{[4-(5-Ethyl-1,3,4-oxadiazol-2-yl)phenyl]diazenyl}phenol* **(8d)**

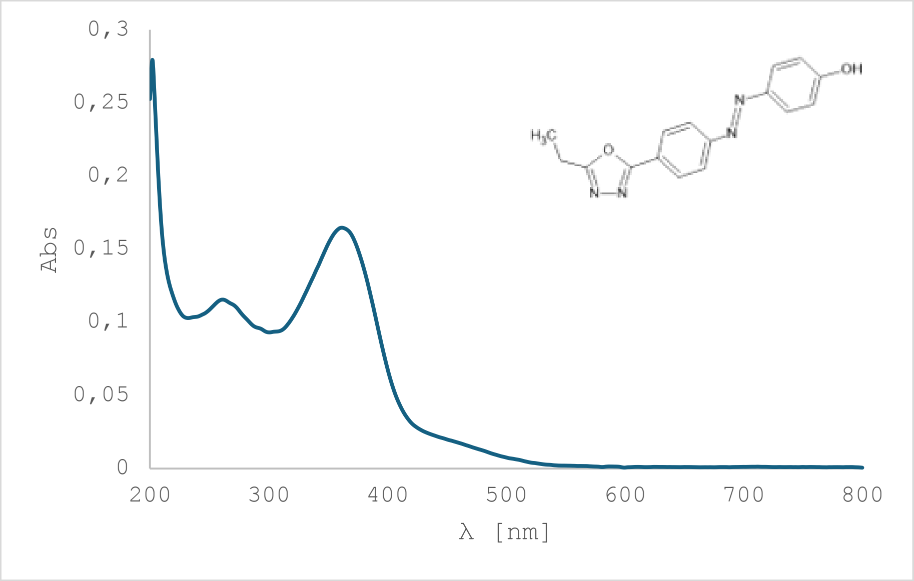

Figure S65. UV-Vis spectra (CH_3OH) of *4-{[4-(5-Ethyl-1,3,4-oxadiazol-2-yl)phenyl]diazenyl}phenol* **(8d)**

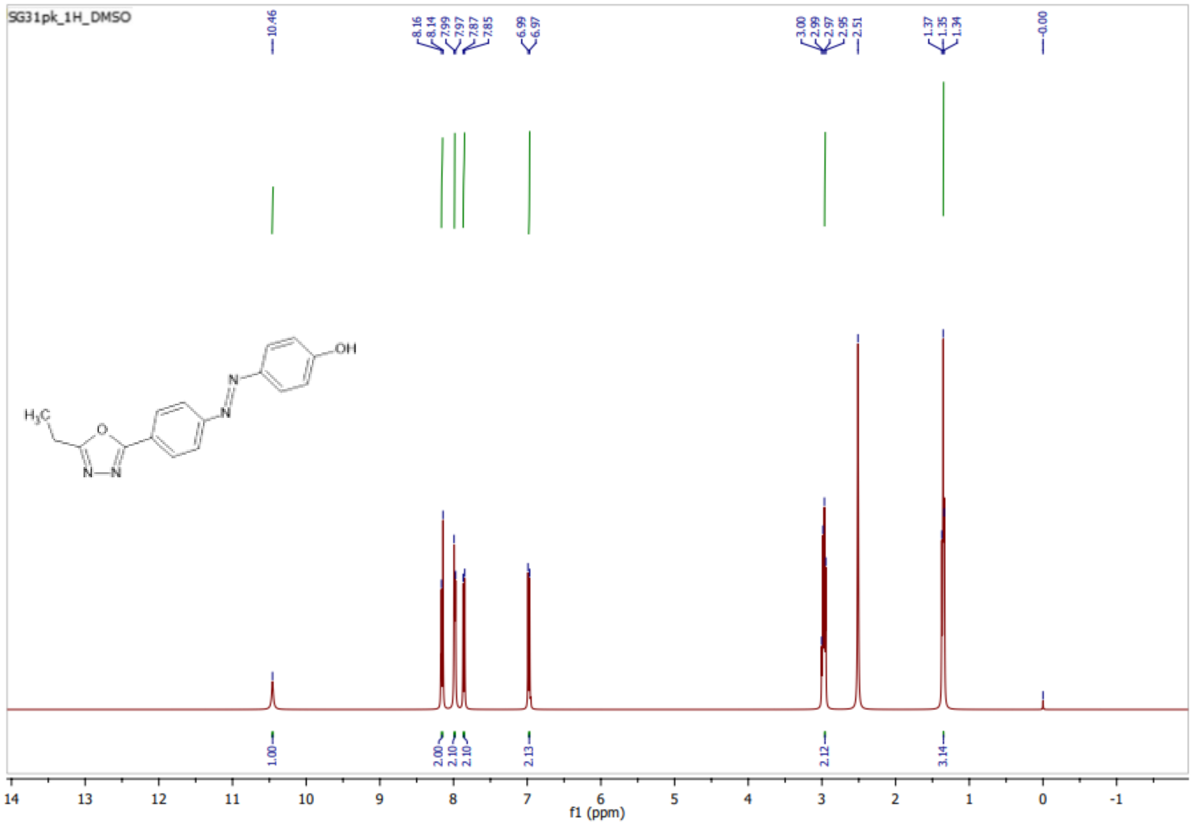

Figure S66. ^{1}H-NMR spectra (400 MHz, DMSO) of *4-{[4-(5-Ethyl-1,3,4-oxadiazol-2-yl)phenyl]diazenyl}phenol* **(8d)**

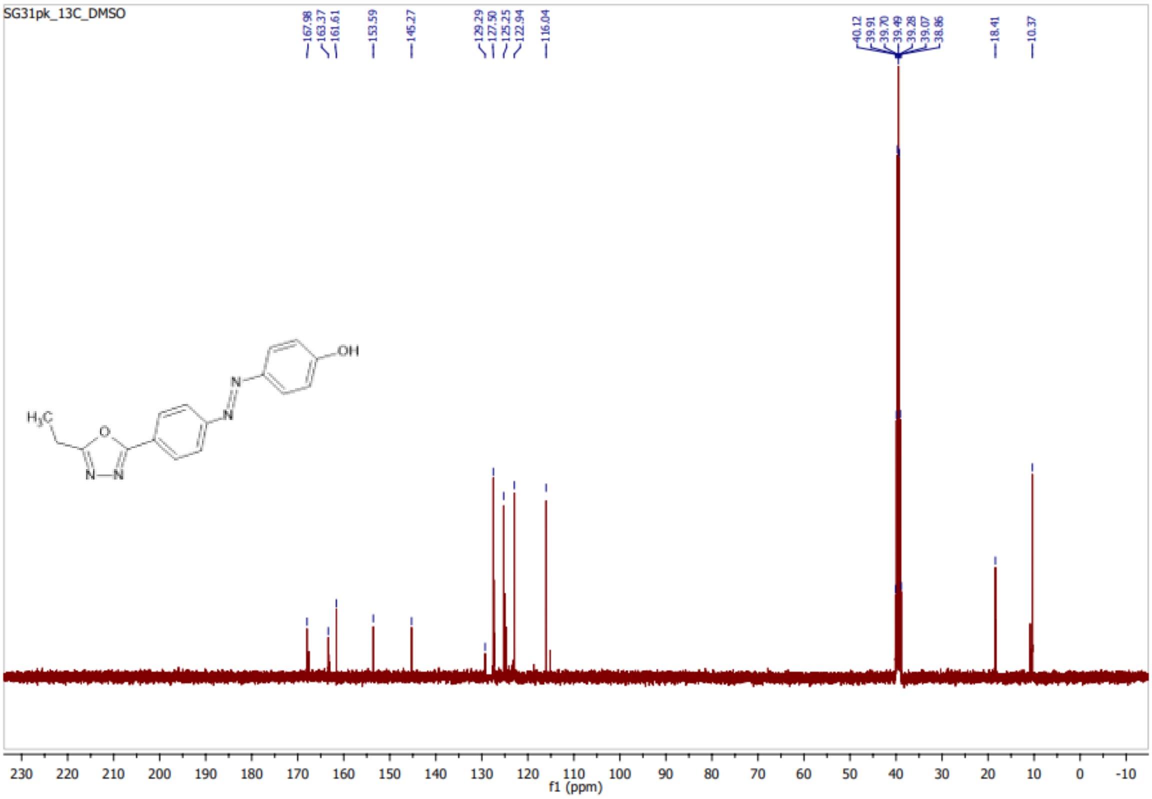

Figure S67. ^{13}C-NMR spectra (100 MHz, DMSO) of *4-{[4-(5-Ethyl-1,3,4-oxadiazol-2-yl)phenyl]diazenyl}phenol* **(8d)**

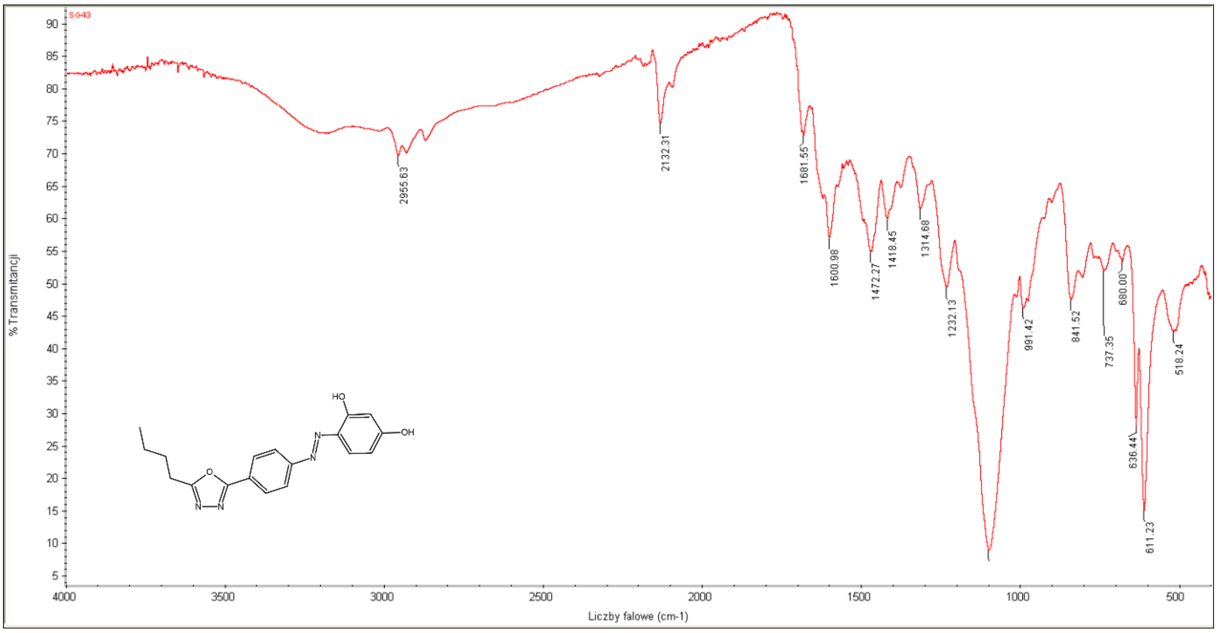

Figure S68. IR spectra of *4-{[4-(5-Butyl-1,3,4-oxadiazol-2-yl)phenyl]diazenyl}benzene-1,3-diol* **(9a)**

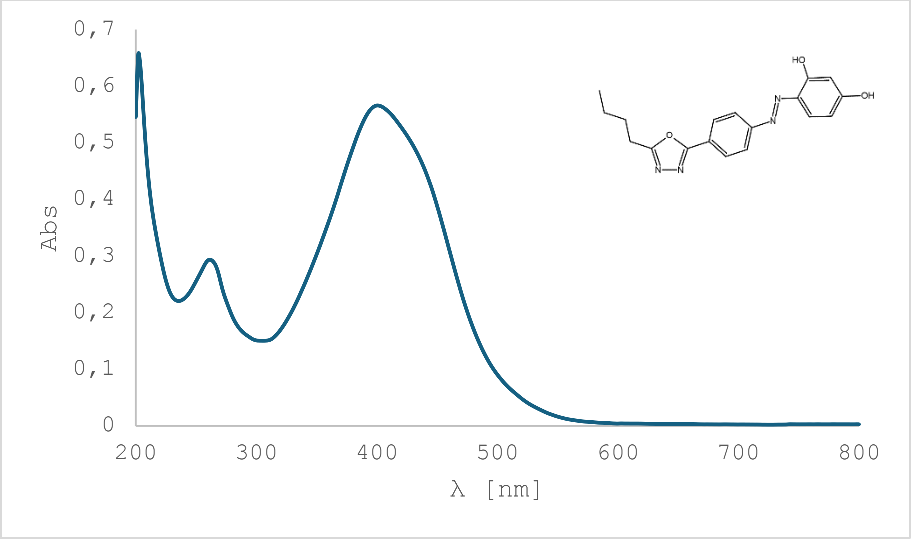

Figure S69. UV-Vis spectra (CH_3OH) of *4-{[4-(5-Butyl-1,3,4-oxadiazol-2-yl)phenyl]diazenyl}benzene-1,3-diol* **(9a)**

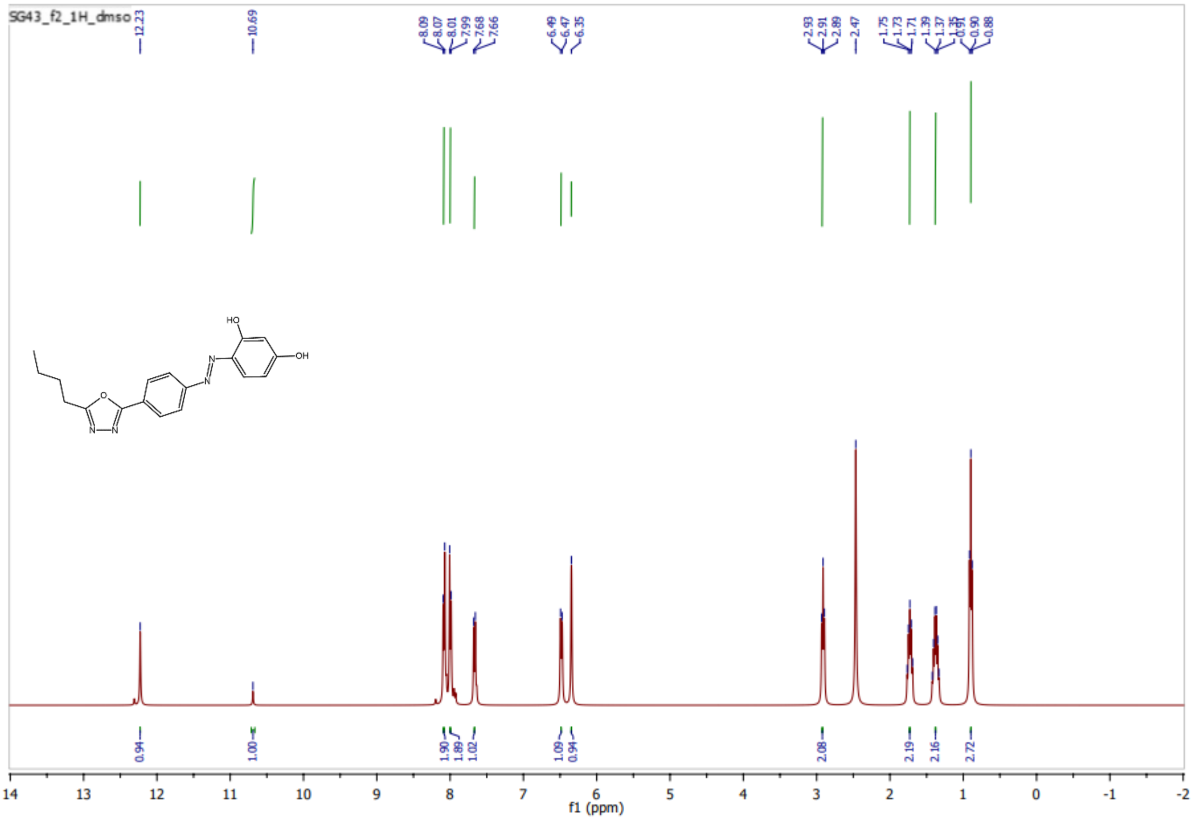

Figure S70. ^1H-NMR spectra (400 MHz, DMSO) of *4-{[4-(5-Butyl-1,3,4-oxadiazol-2-yl)phenyl]diazenyl}benzene-1,3-diol* **(9a)**

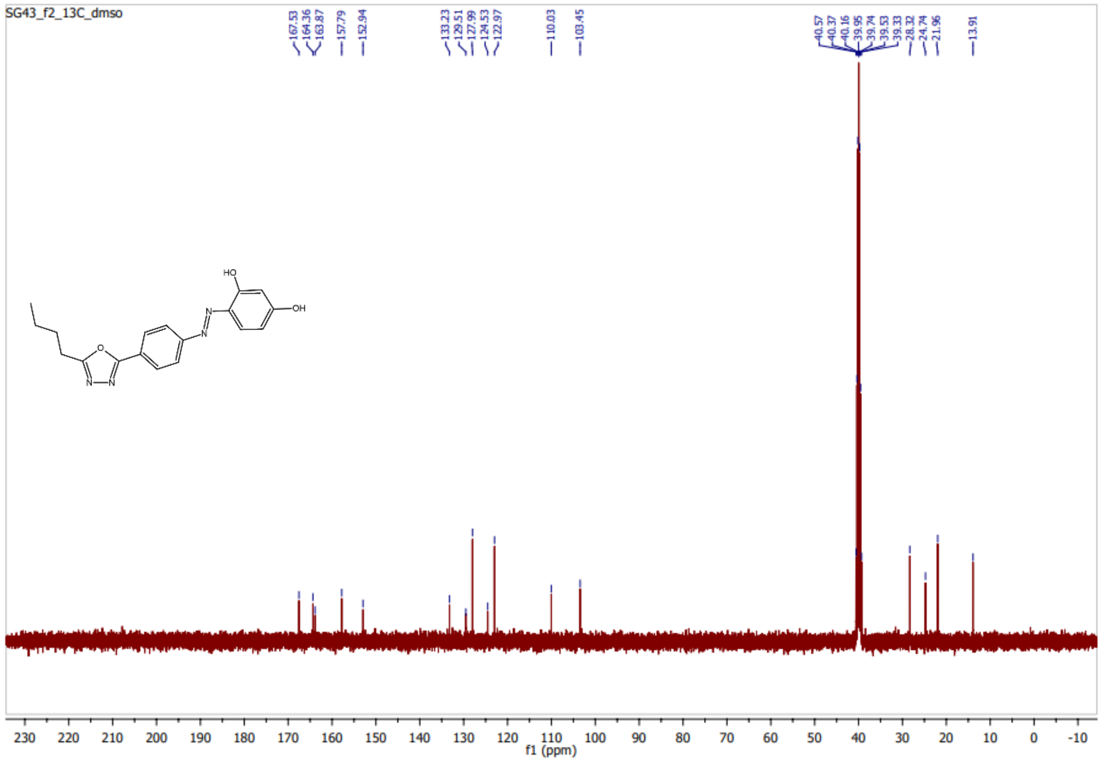

Figure S71. ^{13}C-NMR spectra (100 MHz, DMSO) of *4-{[4-(5-Butyl-1,3,4-oxadiazol-2-yl)phenyl]diazenyl}benzene-1,3-diol* **(9a)**

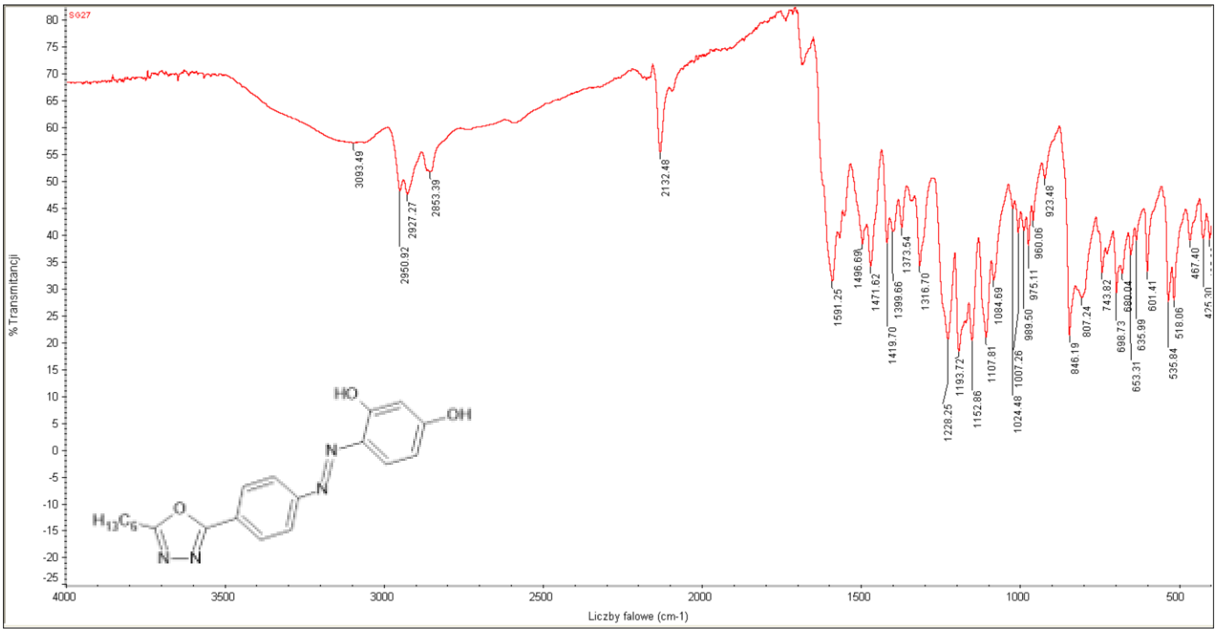

Figure S72. IR spectra of *4-{[4-(5-Hexyl-1,3,4-oxadiazol-2-yl)phenyl]diazenyl}benzene-1,3-diol* **(9b)**

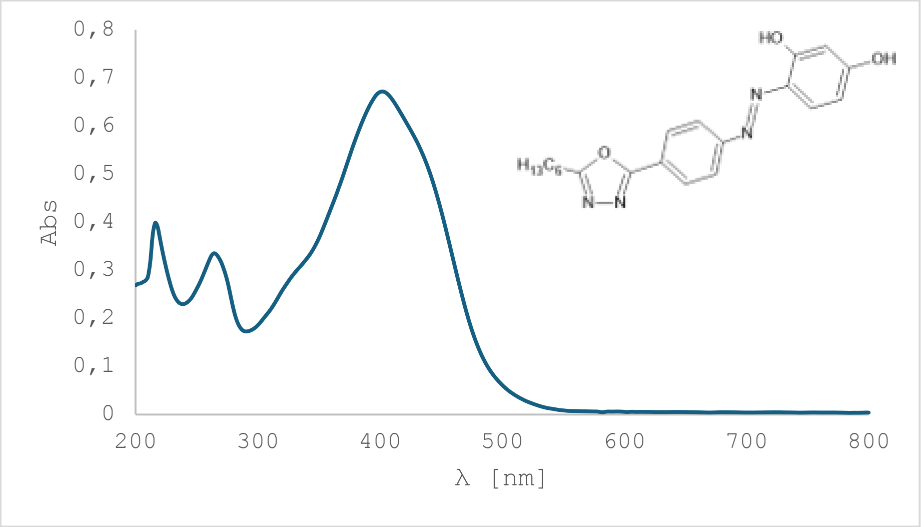

Figure S73. UV-Vis spectra (CH_3OH) of *4-{[4-(5-Hexyl-1,3,4-oxadiazol-2-yl)phenyl]diazenyl}benzene-1,3-diol* **(9b)**

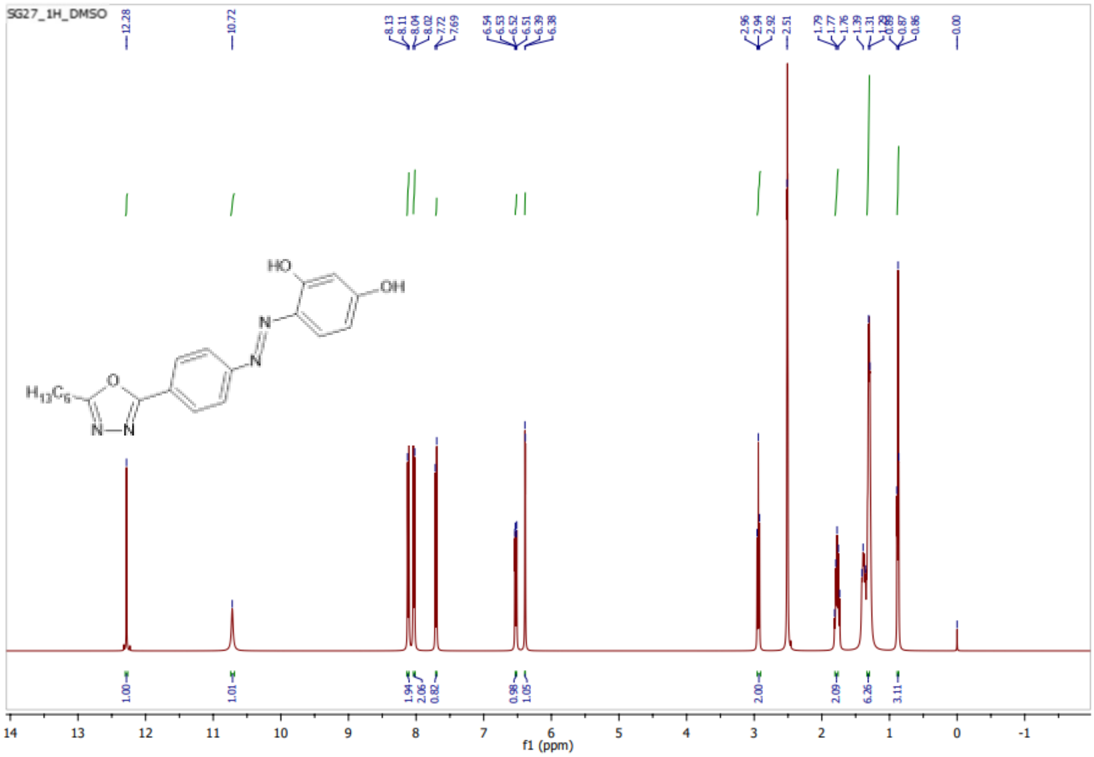

Figure S74. ^{1}H-NMR spectra (400 MHz, DMSO) of *4-{[4-(5-Heksyl-1,3,4-oxadiazol-2-yl)phenyl]diazenyl}benzene-1,3-diol* **(9b)**

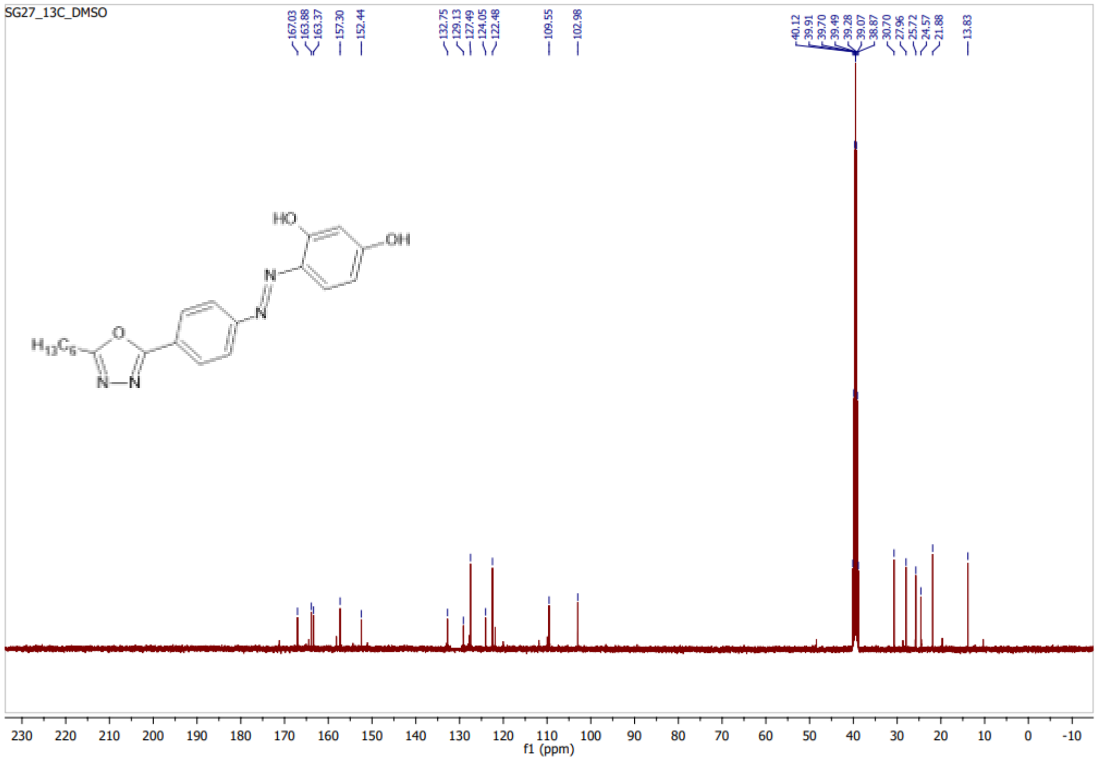

Figure S75. ^{13}C-NMR spectra (100 MHz, DMSO) of *4-{[4-(5-Heksyl-1,3,4-oxadiazol-2-yl)phenyl]diazenyl}benzene-1,3-diol* **(9b)**

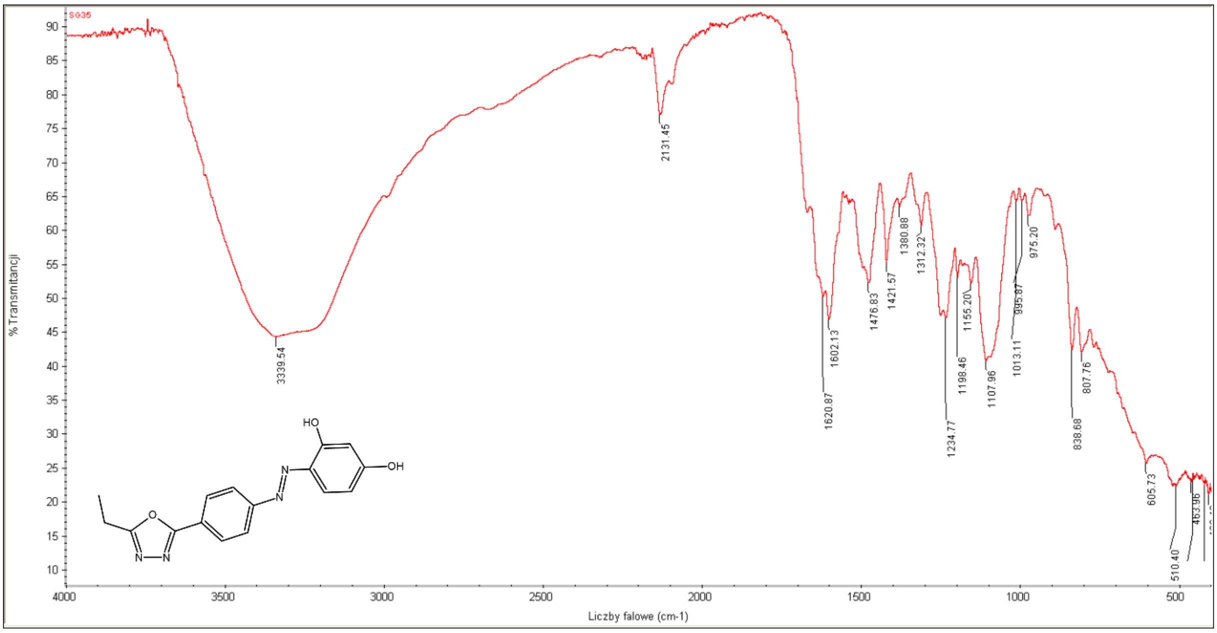

Figure S76. IR spectra of *4-{[4-(5-Ethyl-1,3,4-oxadiazol-2-yl)phenyl]diazenyl}benzene-1,3-diol* **(9d)**

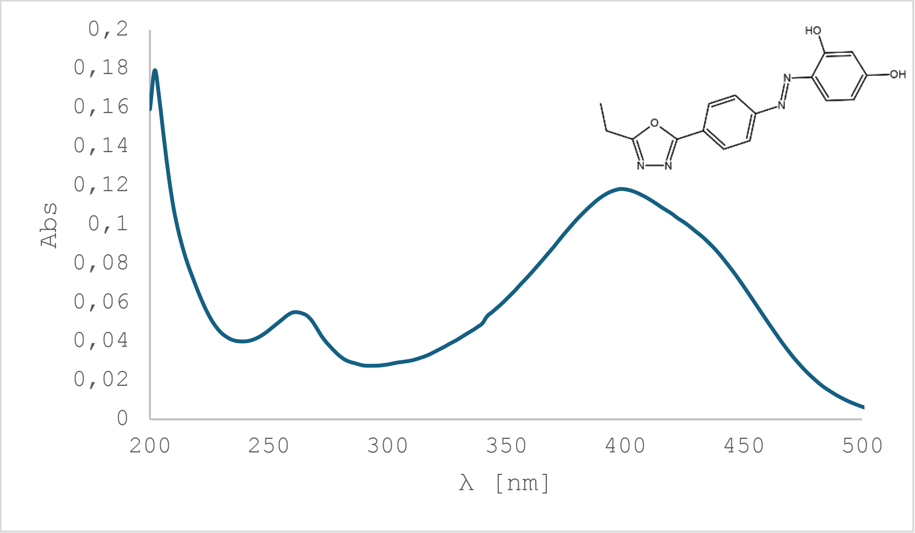

Figure S77. UV-Vis spectra (CH_3OH) of *4-{[4-(5-Ethyl-1,3,4-oxadiazol-2-yl)phenyl]diazenyl}benzene-1,3-diol* **(9d)**

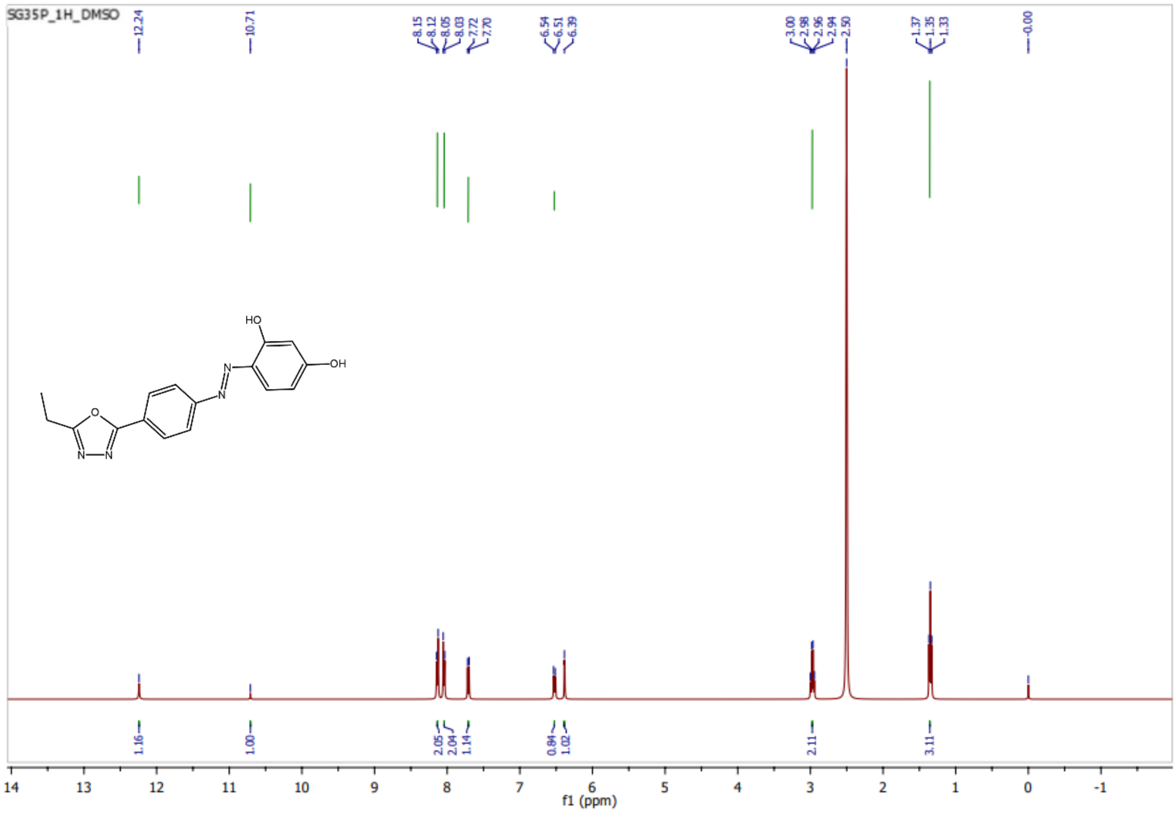

Figure S78. ¹H-NMR spectra (400 MHz, DMSO) of *4-{[4-(5-Ethyl-1,3,4-oxadiazol-2-yl)phenyl]diazenyl}benzene-1,3-diol* **(9d)**

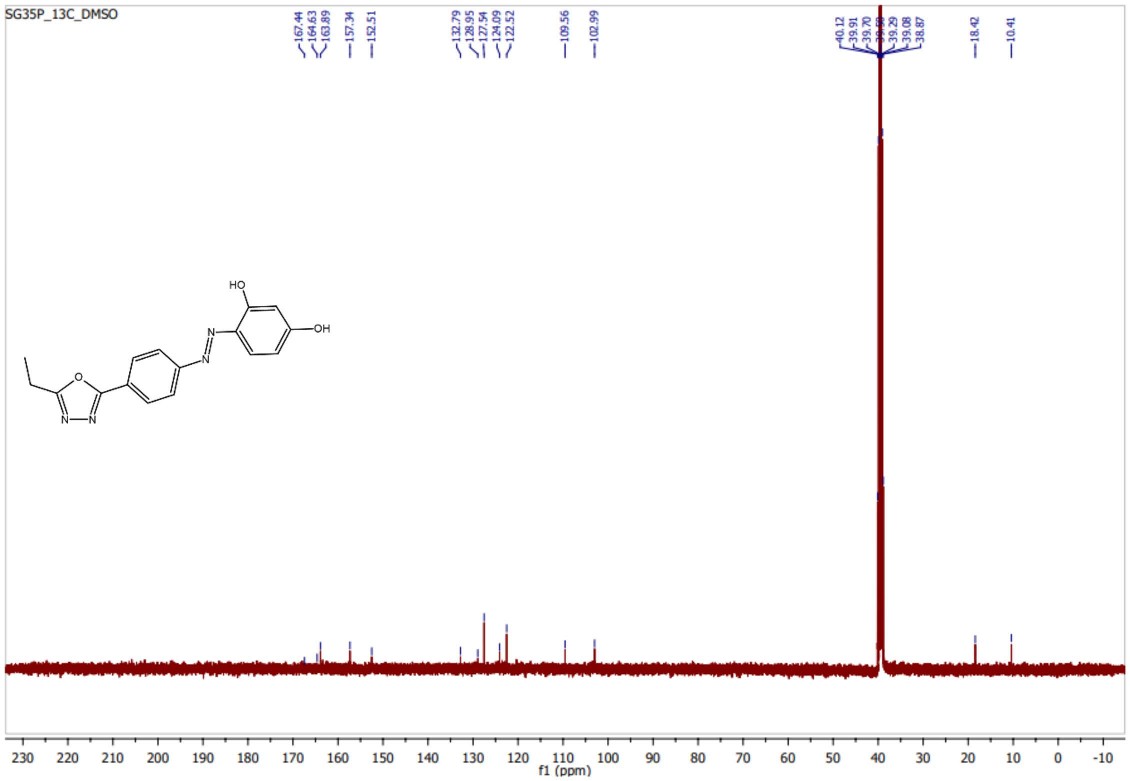

Figure S79. ^{13}C-NMR spectra (100 MHz, DMSO) of *4-{[4-(5-Ethyl-1,3,4-oxadiazol-2-yl)phenyl]diazenyl}benzene-1,3-diol* **(9d)**

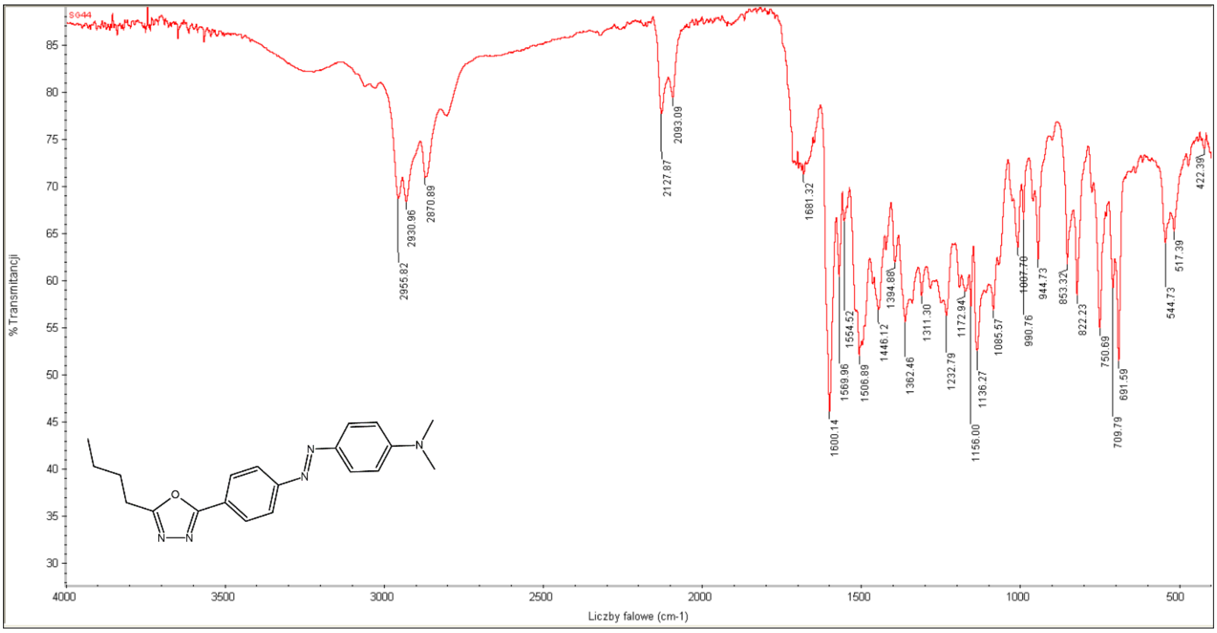

Figure S80. IR spectra of *4-{[4-(5-Butyl-1,3,4-oxadiazol-2-yl)phenyl]diazenyl}-N,N-dimethylaniline* **(10a)**

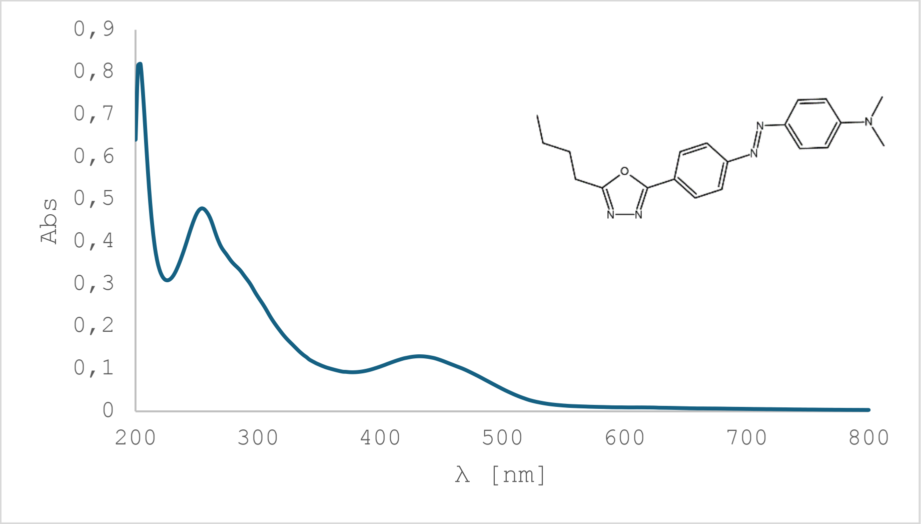

Figure S81. UV-Vis spectra (CH_3OH) of *4-{[4-(5-Butyl-1,3,4-oxadiazol-2-yl)phenyl]diazenyl}-N,N-dimethylaniline* **(10a)**

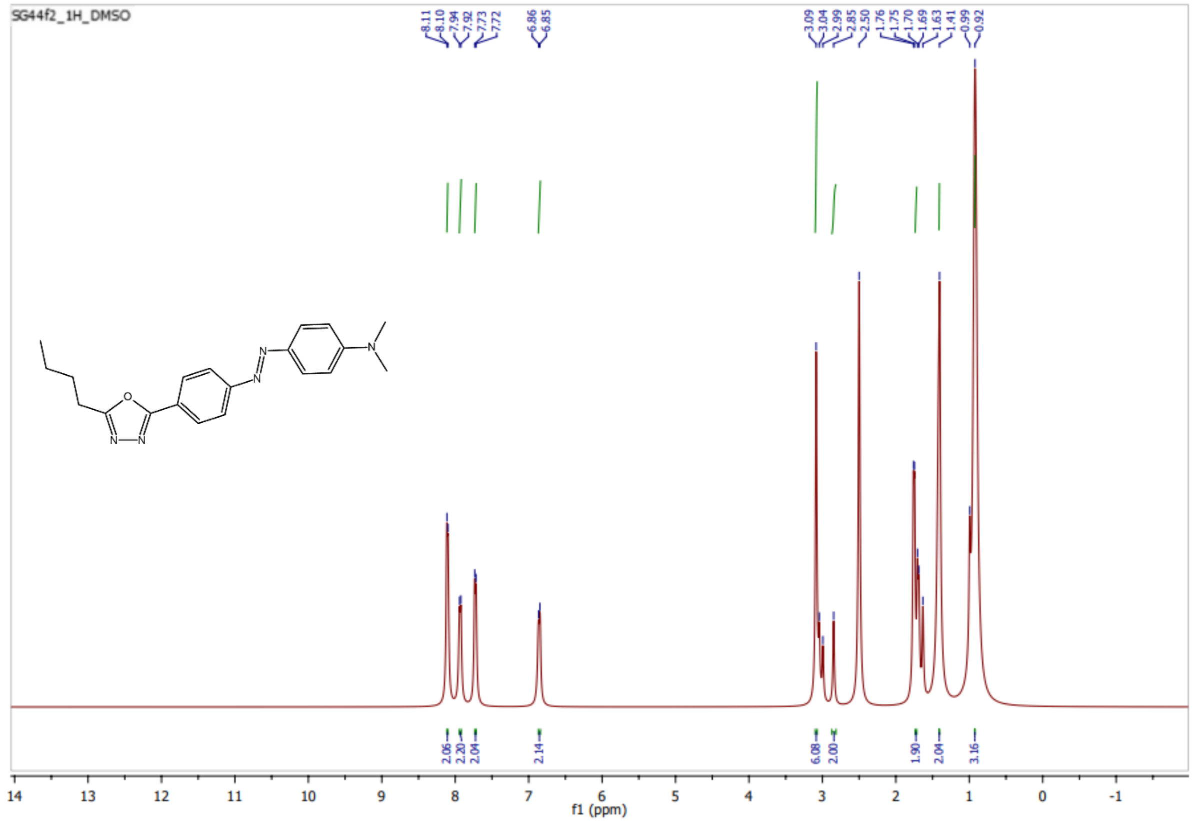

Figure S82. ^{1}H-NMR spectra (400 MHz, DMSO) of *4-{[4-(5-Butyl-1,3,4-oxadiazol-2-yl)phenyl]diazenyl}-N,N-dimethylaniline* **(10a)**

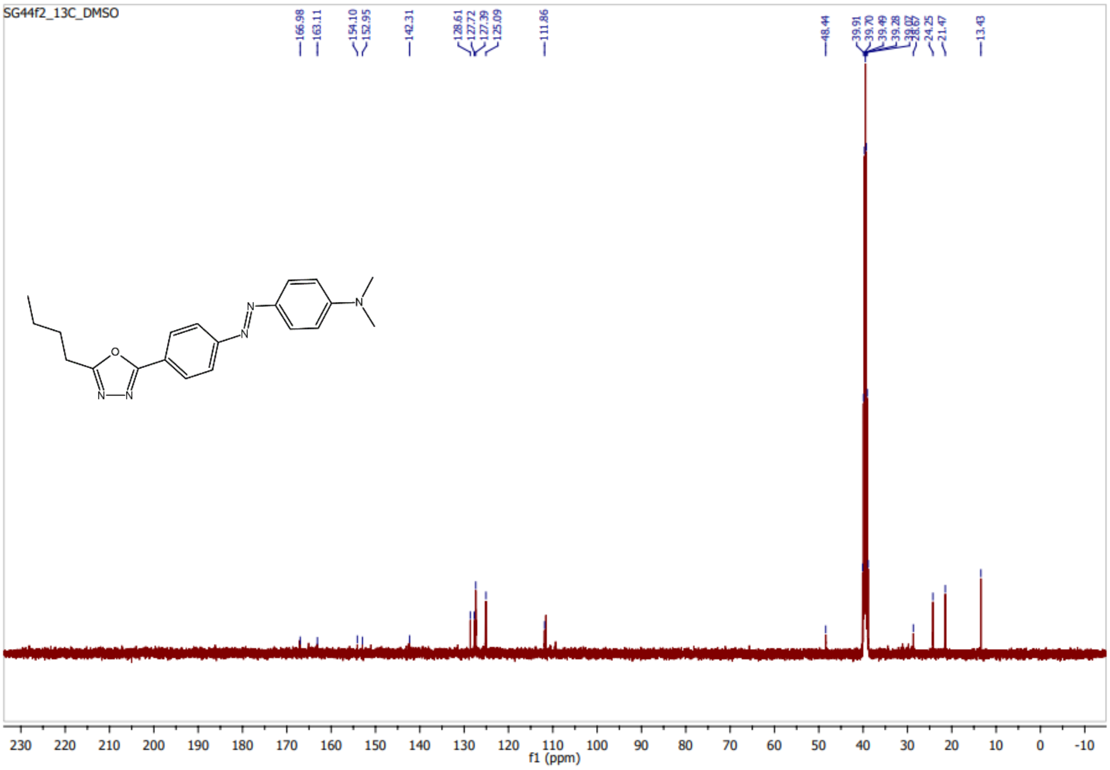

Figure S83. ^{13}C-NMR spectra (100 MHz, DMSO) of *4-{[4-(5-Butyl-1,3,4-oxadiazol-2-yl)phenyl]diazenyl}-N,N-dimethylaniline* **(10a)**

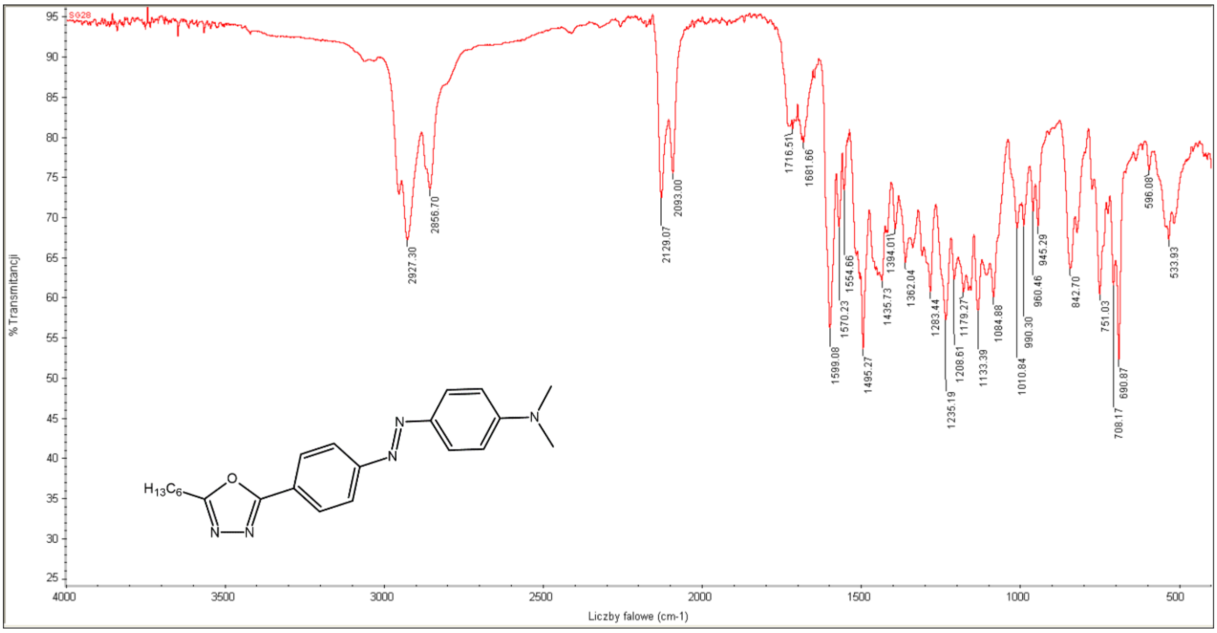

Figure S84. IR spectra of *4-{[4-(5-Hexyl-1,3,4-oxadiazol-2-yl)phenyl]diazenyl}-N,N-dimethylaniline* **(10b)**

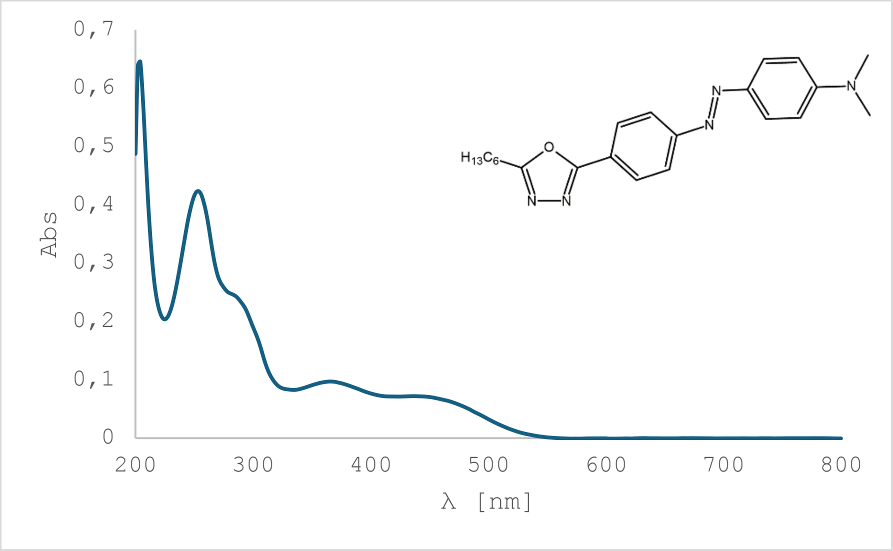

Figure S85. UV-Vis spectra (CH_3OH) of *4-{[4-(5-Hexyl-1,3,4-oxadiazol-2-yl)phenyl]diazenyl}-N,N-dimethylaniline* **(10b)**

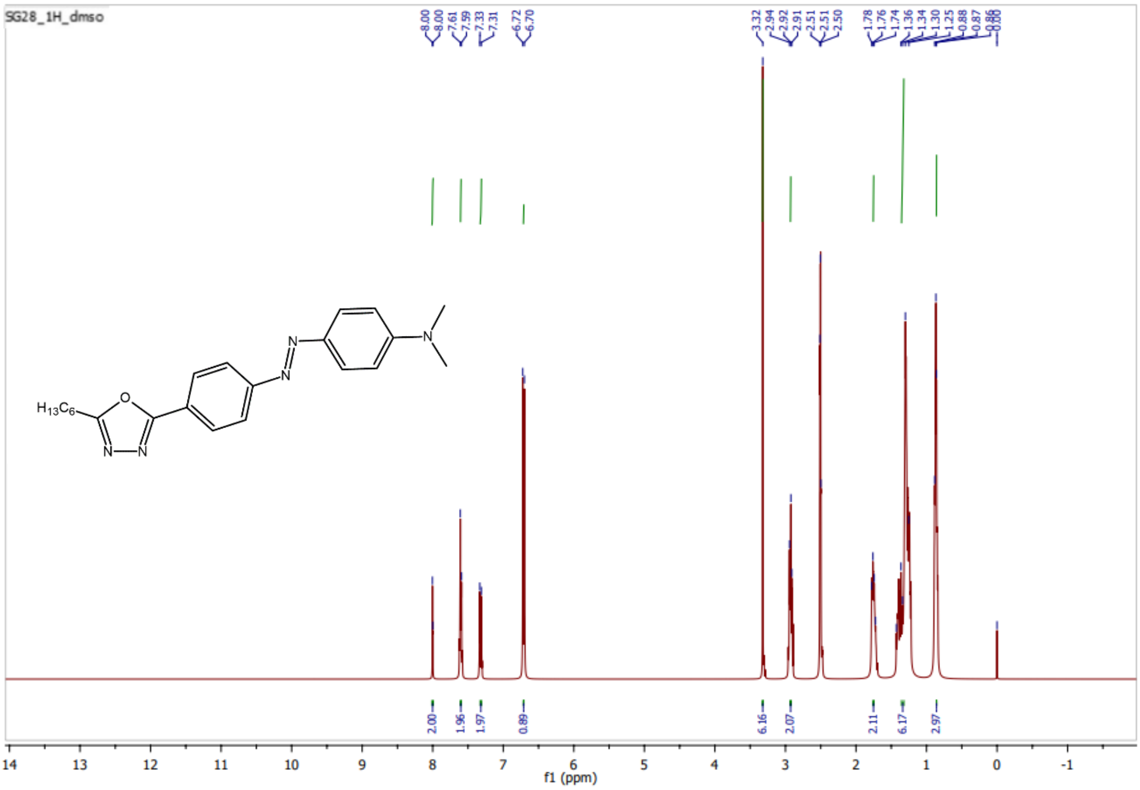

Figure S86. ^{1}H-NMR spectra (400 MHz, DMSO) of *4-{[4-(5-Heksyl-1,3,4-oxadiazol-2-yl)phenyl]diazenyl}-N,N-dimethylaniline* **(10b)**

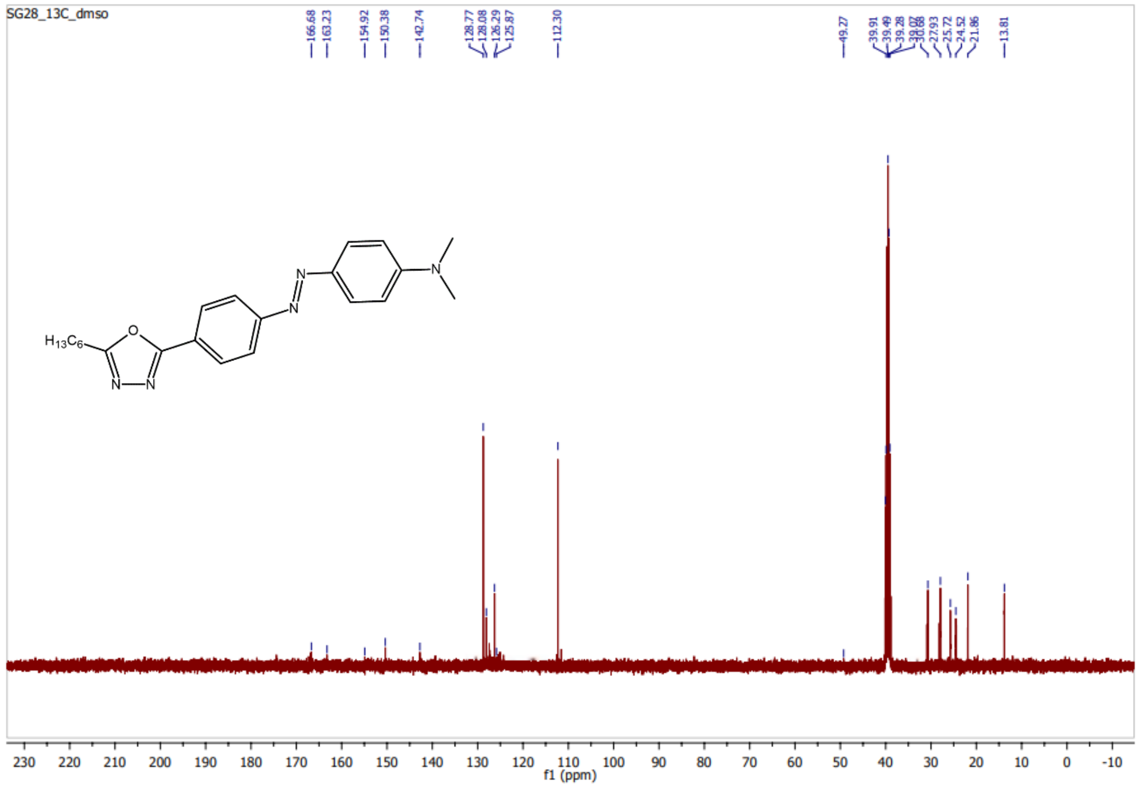

Figure S87. ^{13}C-NMR spectra (100 MHz, DMSO) of *4-{[4-(5-Heksyl-1,3,4-oxadiazol-2-yl)phenyl]diazenyl}-N,N-dimethylaniline* **(10b)**

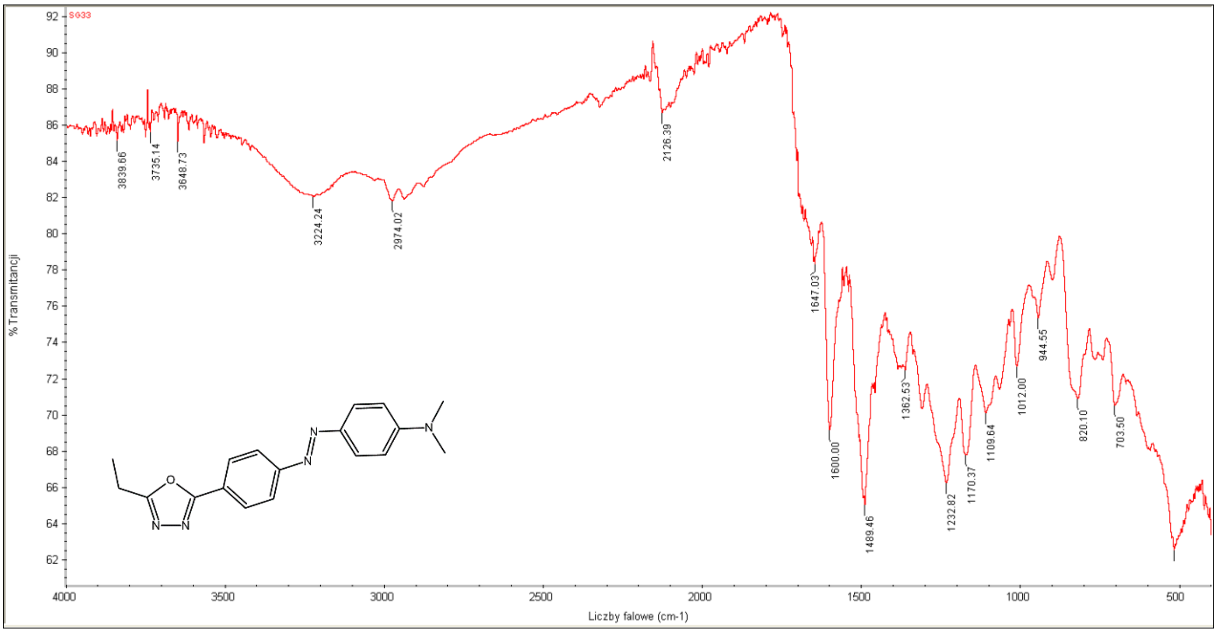

Figure S88. IR spectra of *4-{[4-(5-Ethyl-1,3,4-oxadiazol-2-yl)phenyl]diazenyl}-N,N-dimethylaniline* **(10d)**

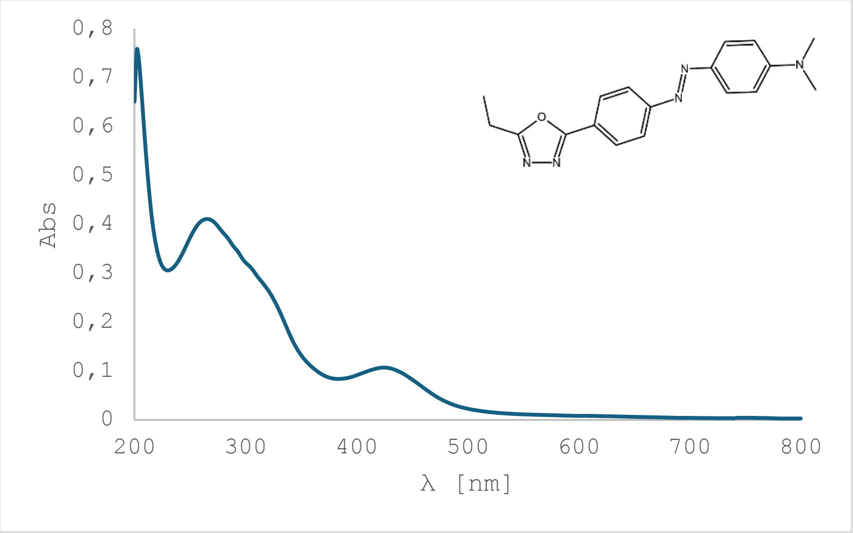

Figure S89. UV-Vis spectra (CH_3OH) of *4-{[4-(5-Ethyl-1,3,4-oxadiazol-2-yl)phenyl]diazenyl}-N,N-dimethylaniline* **(10d)**

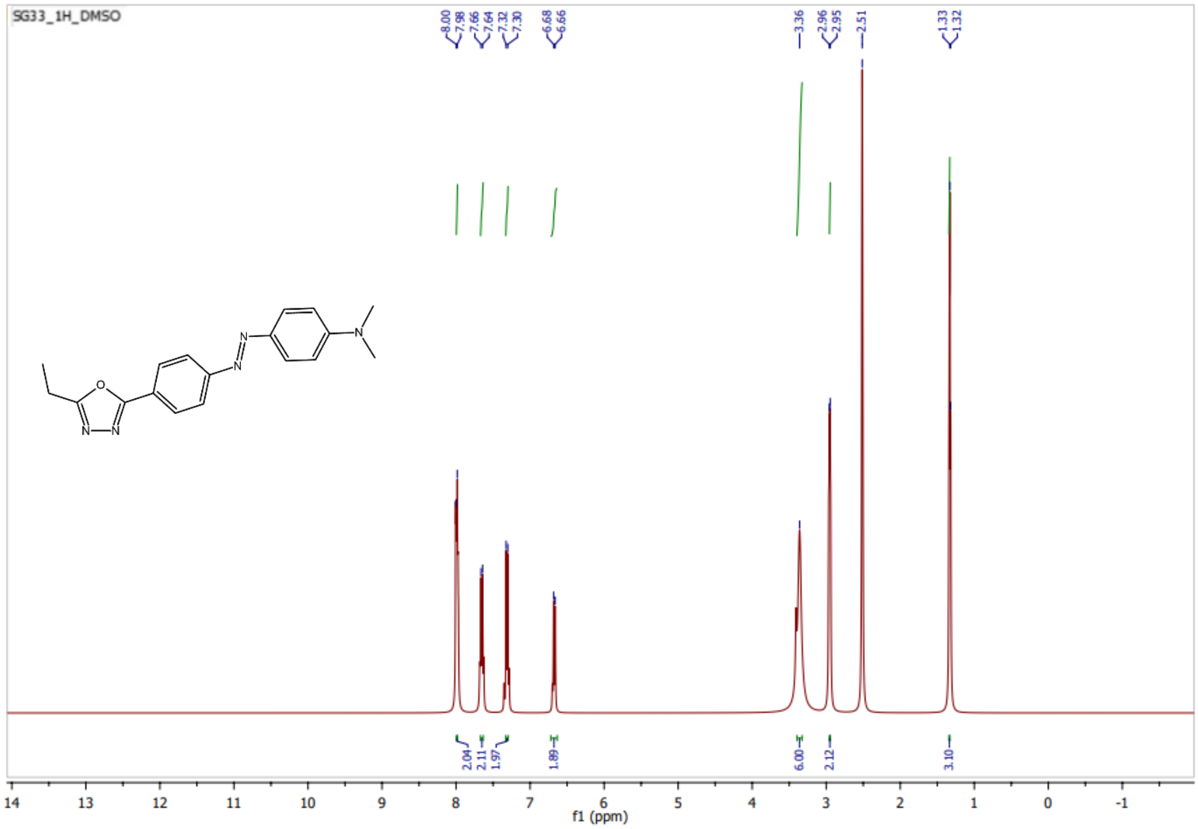

Figure S90 ^{1}H-NMR spectra (400 MHz, DMSO) of *4-{[4-(5-Ethyl-1,3,4-oxadiazol-2-yl)phenyl]diazenyl}-N,N-dimethylaniline* **(10d)**

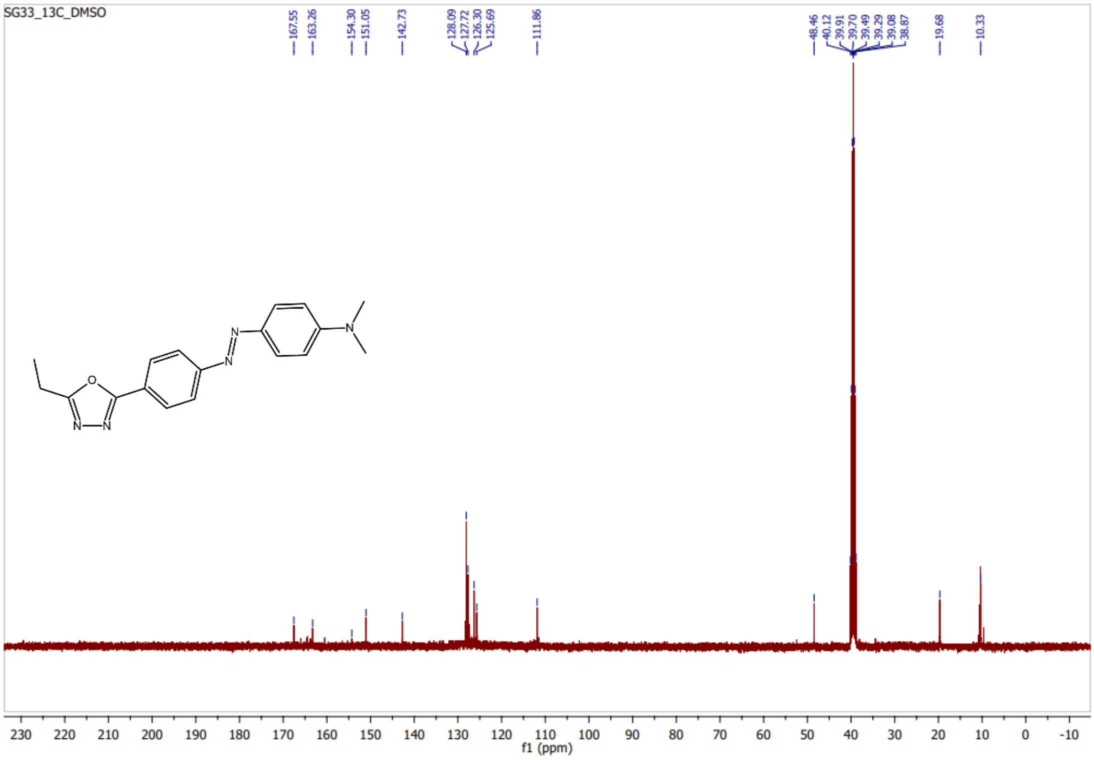

Figure S91. ^{13}C-NMR spectra (100 MHz, DMSO) of *4-{[4-(5-Ethyl-1,3,4-oxadiazol-2-yl)phenyl]diazenyl}-N,N-dimethylaniline* **(10d)**
